# Artificial intelligence−driven analysis identifies arthroscopic shoulder surgery, meniscus injury and treatment, and total knee arthroplasty design biomechanics as the most commonly published topics in *Knee Surgery, Sports Traumatology, Arthroscopy*

**DOI:** 10.1002/jeo2.70341

**Published:** 2025-07-13

**Authors:** Henry Baird, Prudhvi Kodali, Mauricio Gallegos, William Newton, Sarah Jenkins, Harris Slone, Michael Pullen

**Affiliations:** ^1^ Department of Orthopaedic Surgery University of Virginia Health System Charlottesville Virginia USA; ^2^ College of Medicine Medical University of South Carolina Charleston South Carolina USA; ^3^ Department of Orthopaedics and Physical Medicine Medical University of South Carolina Charleston South Carolina USA

**Keywords:** artificial intelligence, orthopaedic research, publications, topics, trends

## Abstract

**Purpose:**

To utilize advanced topic modelling through the Bidirectional Encoder Representations from Transformers Topic (BERTopic) model to investigate research topics in the journal *Knee Surgery, Sports Traumatology, Arthroscopy (KSSTA)*.

**Methods:**

Titles and abstracts from 7886 original research articles and reviews published in *KSSTA* between 1993 and 2023 were examined using the BERTopic artificial intelligence (AI) model. BERTopic applies contextual embeddings and clustering algorithms to group large textual data efficiently sets into topics based on semantic similarity. The generated AI topics were assessed by frequency (the total number of articles per topic from 1993 to 2023) and popularity trends (‘hot’ or increasing and ‘cold’ or decreasing trends determined by linear regression analyses of topic frequency from 2020 to 2023).

**Results:**

The BERTopic model organized 7410 publications into 33 distinct topics. From 1993 to 2023, the most frequently reported topics included arthroscopic shoulder surgery, meniscus injury and treatment, and total knee arthroplasty (TKA): design biomechanics. Between 2020 and 2023, arthroscopic shoulder surgery, TKA: design biomechanics, and TKA: alignment & kinematics were identified as increasingly popular (‘hot’) topics. Conversely, ankle instability, non‐anterior cruciate ligament (ACL) knee ligament injuries, and ACL reconstruction: bone tunnels showed declining popularity (‘cold’ topics).

**Conclusions:**

This study demonstrates the efficacy of the BERTopic model in analyzing large textual data sets to identify relevant research patterns within orthopaedic literature. The results highlight BERTopic's ability to summarize thousands of articles from *KSSTA* into 33 central topics, underscoring its utility in accurately and efficiently capturing current trends and future directions in orthopaedic sports medicine research.

**Level of Evidence:**

Level IV, systematic review.

**Abbreviations**
ACI: autologous chondrocyte implantation
ACL: anterior cruciate ligament
ACLR: anterior cruciate ligament reconstruction
AI: artificial intelligence
BERT: bidirectional encoder representations from transformers
BERTopic: bidirectional encoder representations from transformers Topic
DB: double‐bundle
FAI: femoroacetabular impingement
HTO: high tibial osteotomy
KSSTA: *Knee Surgery, Sports Traumatology, Arthroscopy*
MSC: mesenchymal stem cell
NLP: natural language processing
OLT: osteochondral lesion of the talus
PFA: patellofemoral arthroplasty
PJI: periprosthetic joint infection
PRP: platelet‐rich plasma
Q1–Q4: citation quartiles (from lowest to highest)
RTS: return to sport
SB: single‐bundle
TKA: total knee arthroplasty
UKA: unicompartmental knee arthroplasty
UMAP: Uniform Manifold Approximation and Projection.

## INTRODUCTION

The cornerstone of advancements in orthopaedic surgery is innovative research within peer‐reviewed journals [28]. In a bibliometric analysis by Sun et al. [30], there was an exponential increase in orthopaedic publications, with nearly 41,196 publications tallied in 2020 compared to only 29,001 in 2017. At the forefront of evidence‐based medicine in orthopaedics is the journal, *Knee Surgery, Sports Traumatology, Arthroscopy (KSSTA)*. Nevertheless, overall medical knowledge continues to accelerate and double every few months, which is expected to accelerate even further [1, 7, 10, 29]. This rapid expansion of data highlights the necessity for cutting‐edge technologies, such as artificial intelligence (AI), to efficiently process and consolidate vast amounts of information in the field of sports medicine. In the past, manual bibliometric analysis was the typical method for uncovering large patterns within research, but it has become more time and labour‐intensive, compelling new solutions that are more efficient and large‐scale like AI‐driven topic modelling [4, 25].

AI is starting to emerge as a potential answer to this problem as new ways to integrate AI into orthopaedics are being used [2, 26]. Through natural language processing (NLP) using modelling techniques like the Bidirectional Encoder Representations from Transformers Topic (BERTopic) model, large quantities of textual data from medical journals can be analyzed [3, 8, 12, 13, 16, 17, 18, 22, 27, 31]. As an open‐source Python library, BERTopic is a freely available NLP tool. This model can be used with programming environments like Jupyter Notebook. Jupyter Notebook uses contextual information from pretrained models to categorize large data sets into key topics. With a minimal understanding of Python programming and the use of developer‐provided tutorials, users are able to access and use BERTopic easily. Compared to AI conversational tools like ChatGPT, BERTopic emphasizes data organization and topic modelling instead of building conversation responses.

The purpose of this study was to use the BERTopic model for advanced topic modelling of trends within *KSSTA*. We hypothesized that AI‐driven topic modelling would unveil specific themes and changes in direction within *KSSTA*.

## METHODS

This study did not require approval by our Institutional Review Board as there was no direct involvement of human subjects or use of protected health information.

### Data acquisition and document selection

We searched Scopus, a comprehensive multidisciplinary database, for articles published in *KSSTA* from its inception in 1993 through 31 December 2023. This review incorporated all articles for which the title and abstract text data were available within the Scopus database. As Scopus provides metadata for articles published electronically and in print since the journal's inception, all eligible articles with accessible titles and abstract data were included. Articles without available titles or abstract information in Scopus were excluded. Additionally, editorials, commentaries and other non‐research publications were excluded to ensure that the analysis focused exclusively on original research articles and reviews. These exclusions ensured the analysis accurately reflected original author contributions rather than editorial‐generated content. To comprehensively capture relevant publications, unique identifiers specific to *KSSTA* (ISSN‐0942‐2056 and ISSN‐1433‐7347) were utilized. The initial search identified 8565 documents.

**Figure 1:**
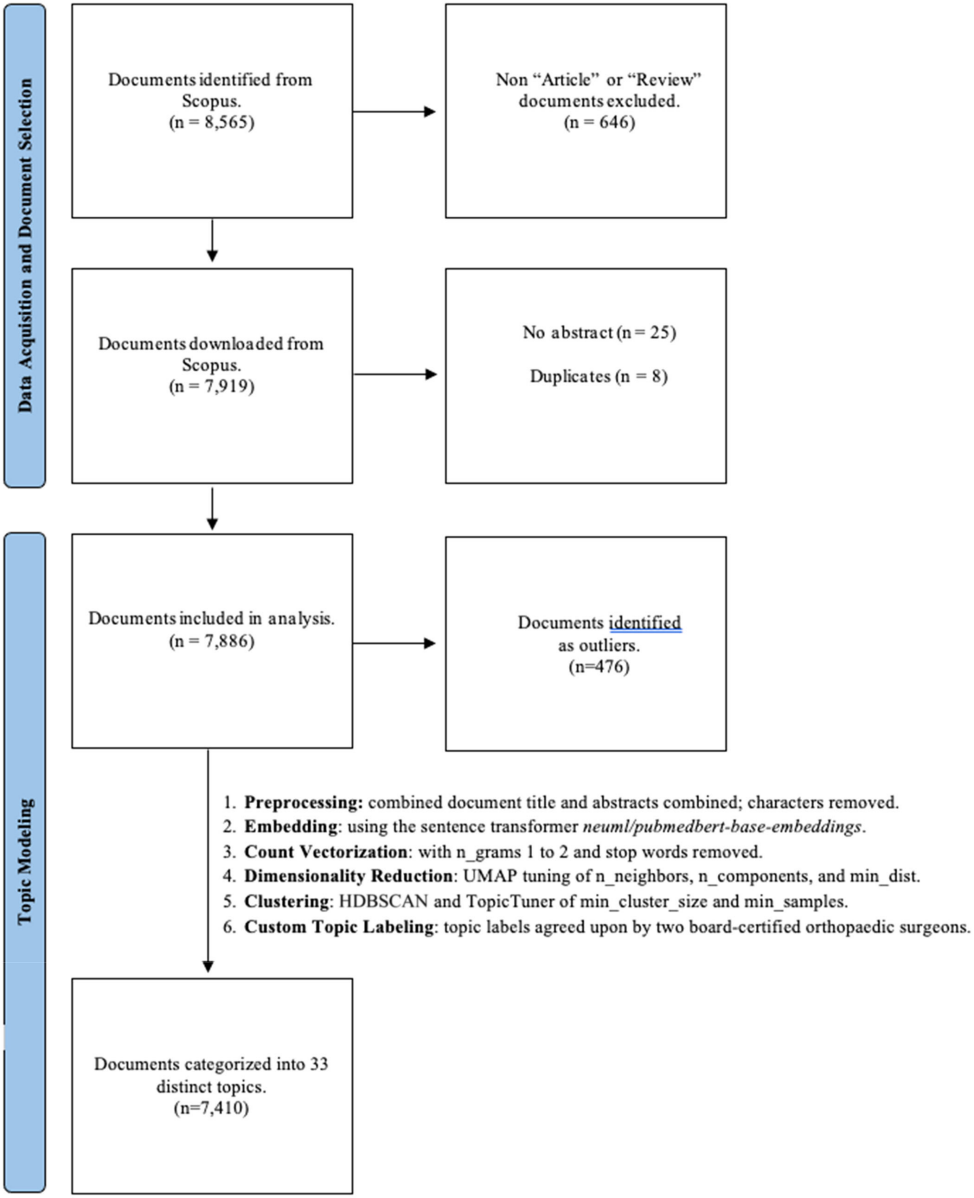
Flowchart depicting the study design, outlining data acquisition, document selection, and the steps involved in topic modelling. It summarizes the process from initial document identification via Scopus to final inclusion in analysis, detailing the exclusion of irrelevant documents and duplicates. HDBSCAN, Hierarchical Density‐Based Spatial Clustering of Applications With Noise; UMAP, Uniform Manifold Approximation and Projection.

Subsequently, we filtered publications categorized as ‘Article’ or ‘Review’, resulting in 7919 documents. Metadata, including document titles, abstracts, authors and affiliations, publication years, and citation counts, were downloaded as comma‐separated value files. Digital Object Identifiers (DOIs) were used to eliminate duplicate entries; documents lacking DOIs were manually reviewed to confirm their uniqueness. Ultimately, 7886 articles were included in the analysis. Figure 1 illustrates the data acquisition and document selection process.

### Topic modelling

We utilized BERTopic, an advanced topic modelling framework that leverages Bidirectional Encoder Representations from Transformers (BERT) embeddings, providing improved topic identification and clustering capabilities compared with conventional methods [8, 12]. Traditional approaches, such as Latent Dirichlet Allocation and Non‐Negative Matrix Factorization, typically rely on bag‐of‐words representations, neglecting semantic relationships between terms and treating documents merely as collections of words [8, 12]. These methods often fail to capture context effectively, resulting in less coherent topic representations. Conversely, BERTopic incorporates contextual embeddings, facilitating more refined and precise clustering of semantically related documents [8, 12]. This approach has significantly advanced the capability of topic modelling for unstructured text data by reducing, although not completely eliminating, assumptions about the underlying corpus. BERTopic's effectiveness has been validated across various domains, including academic, corporate and military contexts [8].

BERTopic assigns documents to topics probabilistically, identifying frequent and specific representative terms. For articles covering multiple topics (e.g., systematic reviews), BERTopic assigns a single topic based on the dominant theme. The model employs contextual embeddings from BERT to evaluate semantic relationships within titles and abstracts, clustering articles into distinct topics based on term density and coherence. When multiple themes existed, the strongest represented topic was selected. Although this enables efficient categorization, it may oversimplify classification for articles substantially overlapping multiple topics. The primary workflow steps included (1) preprocessing data sets for embedding preparation, (2) embedding textual data into multidimensional vectors to capture contextual nuances, (3) count vectorization converting text into token frequency matrices, (4) dimensionality reduction of embedded data to facilitate clustering, (5) clustering to detect dense regions within reduced‐dimensional space and (6) custom naming of topics by two board‐certified orthopaedic sports medicine surgeons. Figure 1 provides an illustration of this workflow. Detailed information can be found in Appendix Tables S1 and S2.

### Statistical analysis and software libraries

To evaluate publication impact and relevance within the field, citation counts per year were calculated and categorized into quartiles (Q1 < Q2 < Q3 < Q4). Citation‐per‐year values were obtained by dividing the total citations for each article by the number of years since publication, using metadata extracted from Scopus. These values were ranked in descending order, and quartiles were established using the inclusive method. Additionally, to examine individual contributions and geographic distribution, senior authors’ names and affiliations were extracted from the metadata. The ten most common journal topics, based on publication volume, were selected for further analysis by publication year, citation quartile and senior authorship. The senior author analysis included only articles where the individual was listed as the senior (last) author. Articles, where the individual was the first or any other author, were excluded to specifically highlight senior author contributions, as the last author typically represents the research team leader responsible for guiding the project's development. For example, articles listing Freddie Fu as the senior author were included; however, if listed elsewhere, the article was excluded from this specific analysis. Focusing on the top ten topics facilitated clear visualization and interpretation, as presenting all topics would be impractical.

Nevertheless, all topics identified through BERTopic were analyzed to capture contemporary topic trends. To identify topic frequency trends from 2020 to 2023, publication counts per topic per year were calculated. Linear regression models were applied, with the year as the independent variable and publication count as the dependent variable. Topics with positive slopes were classified as ‘hot’, indicating increasing frequency, while those with negative slopes were deemed ‘cold’. Topics were then ranked based on regression slopes to highlight the hottest and coldest topics, providing insights into shifting interests within *KSSTA*. The 2020–2023 timeframe offered a contemporary perspective, minimizing biases associated with older data.

All statistical analyses were conducted using Python 3.11 within the Jupyter Notebook environment. The analysis was supported by the following software libraries:

- Data handling and manipulation: pandas, numpy;
- NLP and text analysis: nltk, spacy, scispacy, sentence_transformers, bertopic;
- Data dimension reduction and clustering: sklearn, umap, hdbscan;
- Visualization: topictuner, matplotlib, plotly.

## RESULTS

Of the 7886 documents used to train the BERTopic model, 7410 were grouped into 33 distinct topics, whereas the remaining 476 (6.04%) were categorized as outliers by the model (Figure 1). Table 1 provides a comprehensive overview of the 33 topics, including customized topic names, representative terms, and the total number of associated publications listed by descending frequency.

**Table 1:** Summary of 33 distinct topics with representative terms and number of publications.

Topic	Representative terms	Publications
Arthroscopic shoulder surgery	shoulder, cuff, rotator, rotator cuff, repair, arthroscopic, glenoid, cuff tear, bankart, anchor	870
Meniscus injury and treatment	meniscus, meniscal, tear, meniscectomy, medial meniscus, lateral meniscus, root, extrusion, discoid, medial	570
TKA: Design biomechanics	tka, total knee, knee arthroplasty, arthroplasty, total, design, flexion, prosthesis, gap, insert	502
Patellar instability	mpfl, patellar, patellofemoral, patella, trochlear, patellar instability, tttg, mpfl reconstruction, patellar dislocation, medial patellofemoral	425
ACLR: Single‐ versus double‐bundle	acl, acl reconstruction, reconstruction, doublebundle, graft, anterior cruciate, sb, db, cruciate ligament, cruciate	402
HTO	osteotomy, hto, tibial osteotomy, high tibial, wedge, correction, owhto, high, openwedge, tibial	324
ACI	defect, cartilage, osteochondral, cartilage defect, autologous, chondrocyte, aci, autologous chondrocyte, cartilage repair, chondrocyte implantation	276
Ankle instability	ankle, ankle instability, atfl, lateral ankle, sprain, cfl, chronic ankle, tibiofibular, ankle sprain, syndesmotic	271
ACLR: Graft selection	hamstring, quadriceps, autograft, brace, ht, strength, muscle, graft, qt, hop	267
ACLR: Bone tunnels	tunnel, femoral tunnel, tibial tunnel, femoral, bone tunnel, tunnel position, enlargement, graft, aperture, placement	256
ACLR: Revision	aclr, acl, acl reconstruction, reconstruction, revision, consensus, injury, anterior cruciate, cruciate ligament, cruciate	230
TKA: Outcome measures	expectation, tka, validity, questionnaire, pain, score, hospital, arthroplasty, version, knee arthroplasty	212
Achilles tendon injuries	achilles, achilles tendon, tendon, tendon rupture, rupture, atrs, tendinopathy, achilles tendinopathy, midportion, atr	212
UKA	uka, unicompartmental, unicompartmental knee, arthroplasty, knee arthroplasty, arthroplasty uka, oxford, ukr, revision, medial uka	209
Pivot shift test	pivot shift, pivot, shift, shift test, laxity, test, acceleration, translation, slope, knee laxity	207
TKA: Alignment and kinematics	femoral component, component, alignment, femoral, rotation, axis, aligned, ka, tka, kinematically	206
Hip arthroscopy and FAI	hip, fai, hip arthroscopy, femoroacetabular, femoroacetabular impingement, impingement, labral, acetabular, alpha angle, groin	199
Non‐ACL ligamentous injuries of the knee	pcl, mcl, posterior cruciate, smcl, plc, ligament, posterior, collateral, collateral ligament, pcl reconstruction	158
Athlete injury prevention	player, injury, football, skier, team, season, acl injury, elite, prevention, soccer	157
ACLR: Graft size	notch, bundle, acl, insertion, intercondylar, intercondylar notch, footprint, width, insertion site, pl bundle	147
Graft fixation biomechanics	screw, fixation, interference, interference screw, graft, screw fixation, bioabsorbable, load, pin, device	132
Tibial avulsion fractures	fracture, avulsion fracture, avulsion, plateau fracture, stress fracture, eminence, plateau, tibial plateau, tibial eminence, eminence fracture	119
ACLR: Return to sport	aclr, return, return sport, rts, sport, aclrsi, psychological, preinjury, reconstruction, selfefficacy	119
TKA: Computer‐assisted	navigation, conventional, computerassisted, tka, alignment, conventional tka, approach, ca, computer, navigated	115
Tendon‐bone healing	patellar tendon, tendon, proximal hamstring, hamstring, patellar, rupture, periosteum, healing, tendon rupture, tendonbone	107
TKA: Blood loss	blood, blood loss, tourniquet, transfusion, loss, blood transfusion, txa, tranexamic, tranexamic acid, haemoglobin	101
PJI	infection, septic, septic arthritis, pji, twostage, antibiotic, arthritis, joint infection, vancomycin, periprosthetic	100
Knee phenotyping	varus, alignment, deformity, valgus, phenotype, hka, varus knee, varus deformity, angle, coronal	98
Intra‐articular biologic injections	injection, prp, cell, msc, intraarticular, plasma, stem cell, intraarticular injection, plateletrich plasma, plateletrich	90
Synovial tumours and intra‐articular masses	cyst, ganglion, fat pad, fat, pad, synovial, lipoma, pigmented villonodular, villonodular, pigmented	86
Perioperative pain management	analgesia, analgesic, bupivacaine, mg, morphine, nerve block, consumption, block, postoperative pain, local	86
PFA	resurfacing, patellar resurfacing, pfa, patellar, patellofemoral, patellofemoral arthroplasty, patella, inlay, isolated patellofemoral, patellar thickness	83
OLT	talus, osteochondral, osteochondral lesion, lesion talus, olt, aofas, microfracture, talar, ankle, lesion	74

*Note*: This table details the 33 specific topics identified by the BERTopic model, accompanied by representative terms and the count of associated publications.

Abbreviations: ACI, autologous chondrocyte implantation; ACLR, anterior cruciate ligament reconstruction; FAI, femoroacetabular impingement; HTO, high tibial osteotomy; MSC, mesenchymal stem cell; OLT, osteochondral lesion of the talus; PFA, patellofemoral arthroplasty; PJI, periprosthetic joint infection; TKA, total knee arthroplasty; UKA, unicompartmental knee arthroplasty.

**Figure 2:**
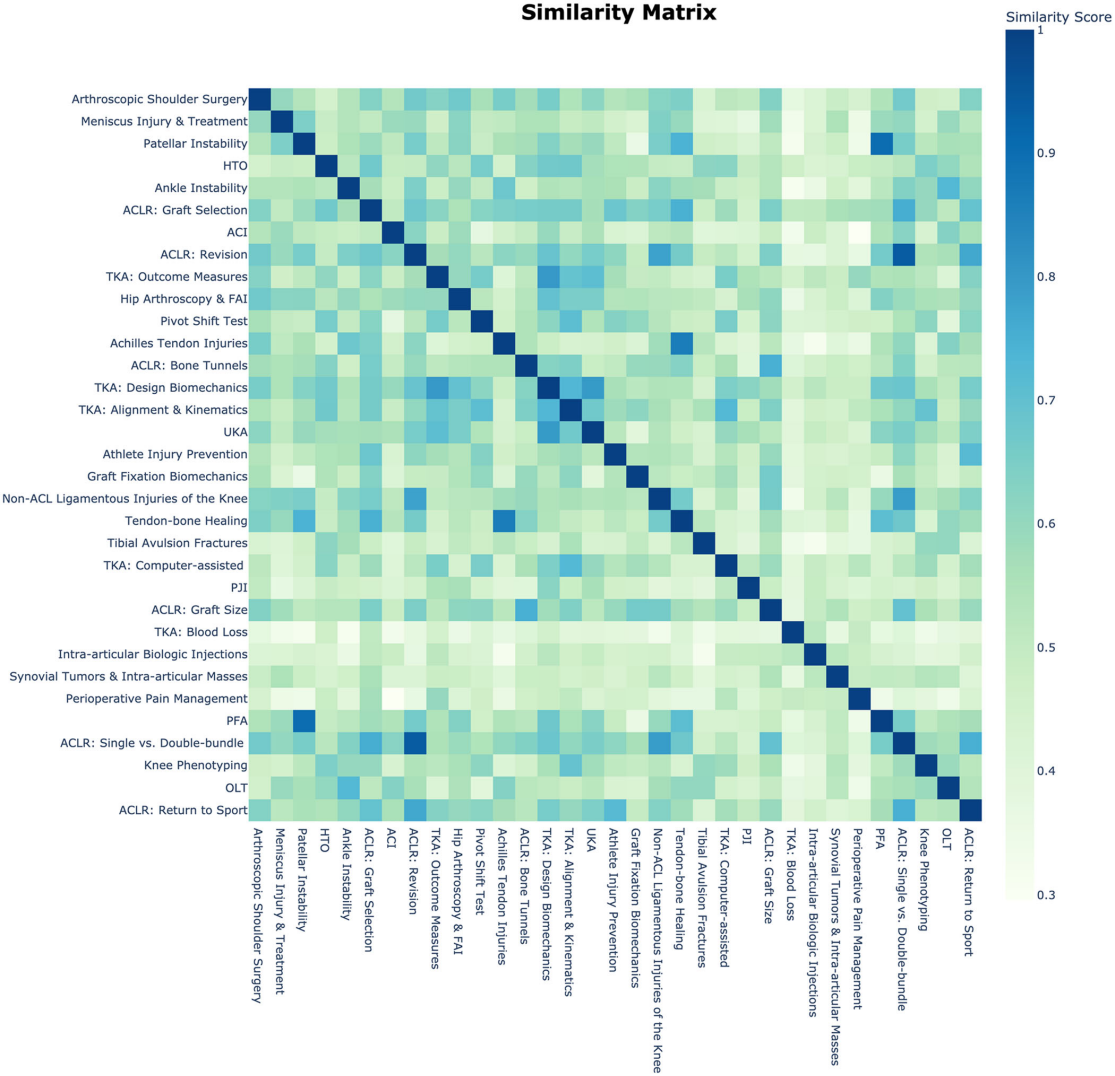
Topic similarity heatmap matrix. The heatmap illustrates the similarity among the 33 topics identified by BERTopic, employing cosine similarity. Darker colours signify greater similarity. ACI, autologous chondrocyte implantation; ACLR, anterior cruciate ligament reconstruction; BERTopic, Bidirectional Encoder Representations from Transformers Topic; FAI, femoroacetabular impingement; HTO, high tibial osteotomy; OLT, osteochondral lesion of the talus; PFA, patellofemoral arthroplasty; PJI, periprosthetic joint infection; TKA, total knee arthroplasty; UKA, unicompartmental knee arthroplasty.

**Figure 3:**
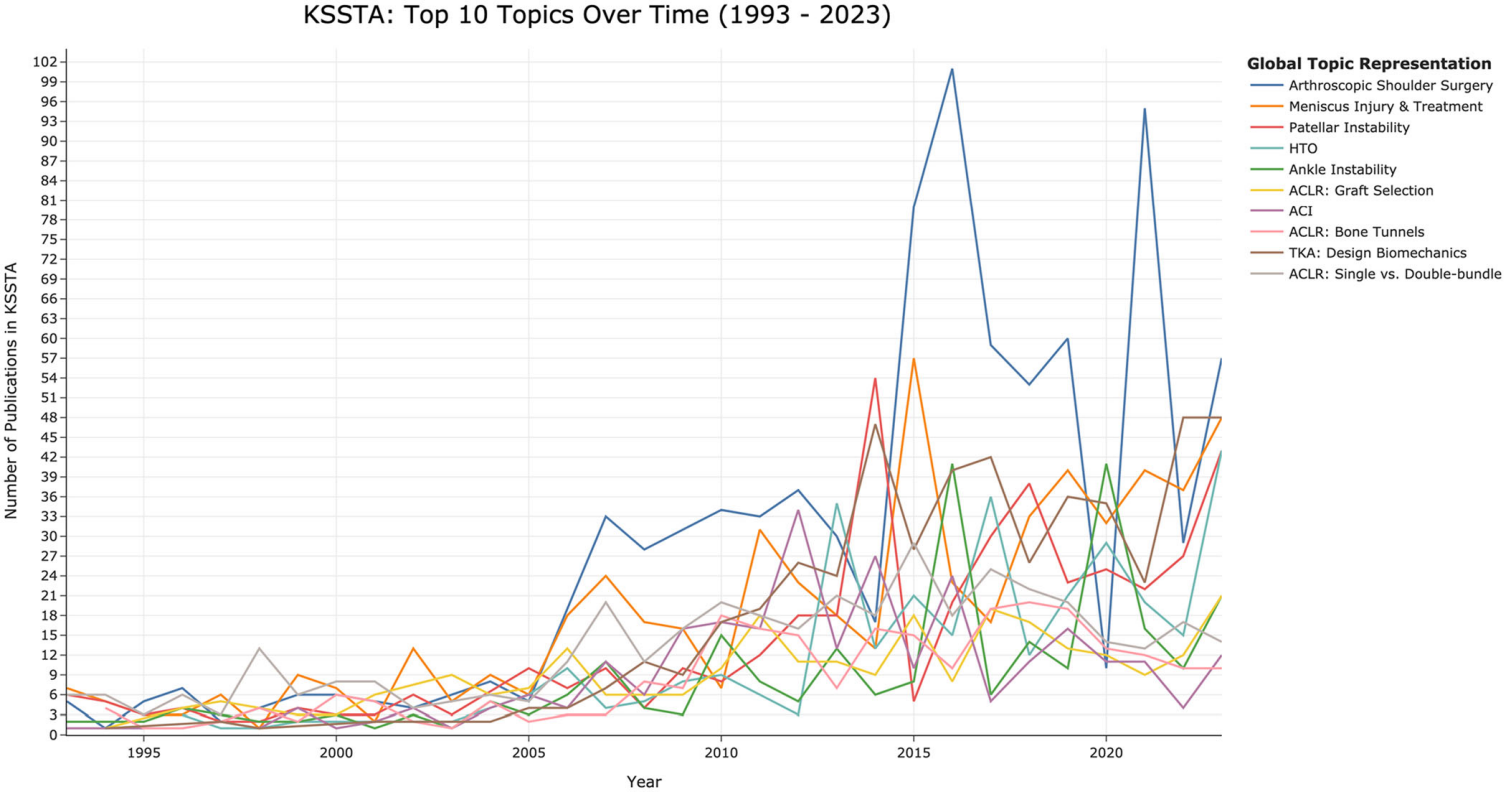
Historical trends of the top 10 topics. This figure illustrates publication trends for the top 10 topics in *KSSTA* from 1993 to 2023. The *x*‐axis represents the publication years, while the *y*‐axis shows the number of publications annually for each topic. ACI, autologous chondrocyte implantation; ACLR, anterior cruciate ligament reconstruction; HTO, high tibial osteotomy; *KSSTA*, *Knee Surgery, Sports Traumatology, Arthroscopy*; TKA, total knee arthroplasty.

**Figure 4:**
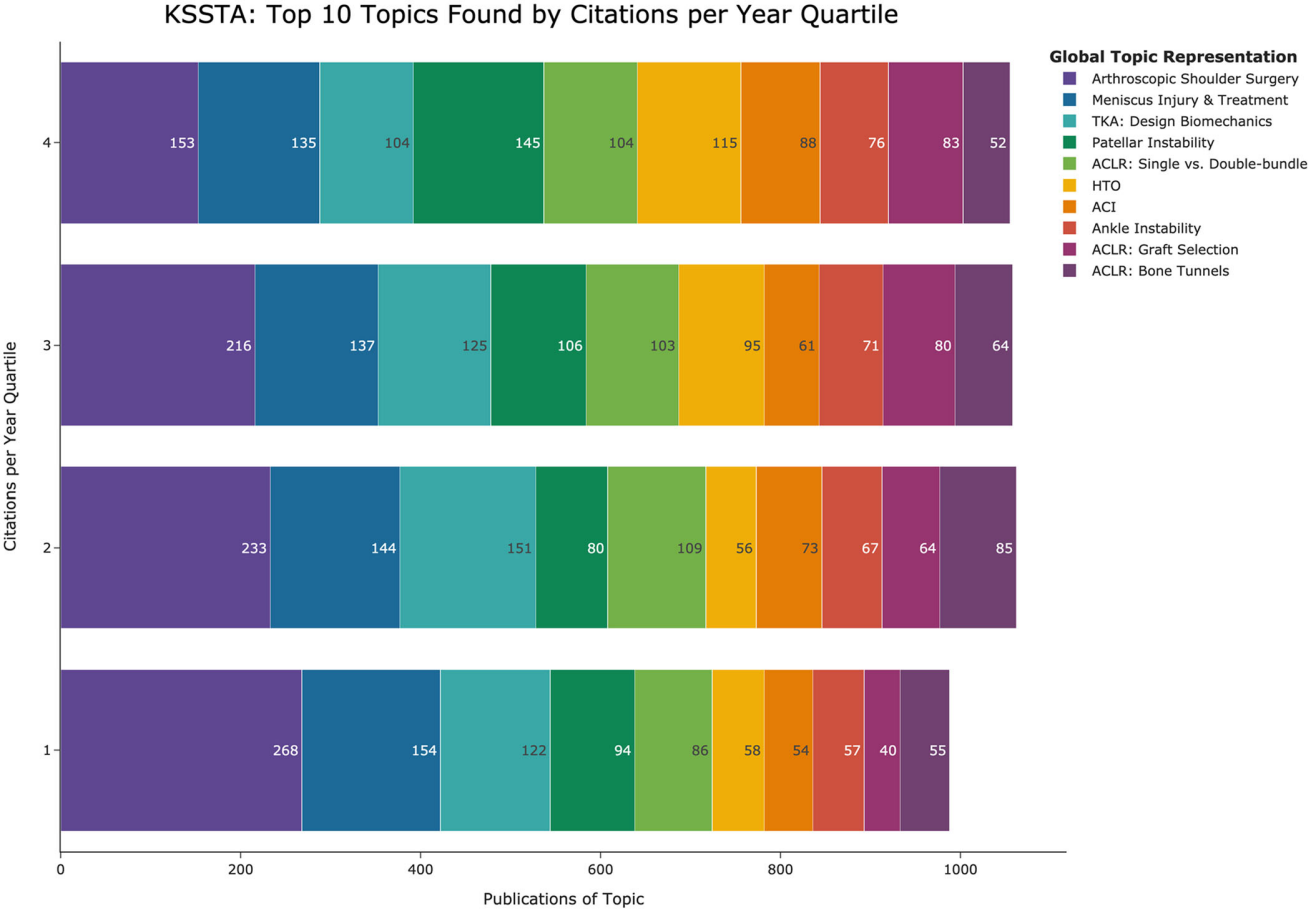
Quartile distribution of citations per year for the top 10 topics. This figure represents citation‐per‐year quartiles for the top 10 topics. The *y*‐axis categorizes citation quartiles from Q1 (lowest) to Q4 (highest), and the *x*‐axis indicates publication counts within each quartile. Purple, arthroscopic shoulder surgery; dark blue, meniscus injury and treatment; light blue, TKA: design biomechanics; dark green, patellar instability; light green, ACLR: single‐ versus double‐bundle; light orange, HTO; dark orange, ACI; red, ankle instability; dark pink, ACLR: graft selection; violet, ACLR: bone tunnels; grey, other topics. ACI, autologous chondrocyte implantation; ACLR, anterior cruciate ligament reconstruction; HTO, high tibial osteotomy; TKA, total knee arthroplasty.

The top 10 topics by frequency included arthroscopic shoulder surgery, meniscus injury and treatment, total knee arthroplasty (TKA): design biomechanics, patellar instability, anterior cruciate ligament reconstruction (ACLR); SB, single‐bundle; DB, double‐bundle; high tibial osteotomy (HTO), autologous chondrocyte implantation (ACI), ankle instability, ACLR: graft selection, and ACLR: bone tunnels. Topic similarities and differences are depicted in a heatmap based on cosine similarities of topic embeddings produced by BERTopic (Figure 2). The heatmap demonstrates relationships among closely related topics, such as patellofemoral arthroplasty and patellar instability. Figure 3 illustrates historical publication data (1993‐2023) for these top 10 topics. Figure 4 shows citation distributions by quartile. HTO publications were frequently cited, with 65% appearing in the highest two citation quartiles (Q4 and Q3), while arthroscopic shoulder surgery publications were less cited, with 58% falling into the lower two citation quartiles (Q2 and Q1).

**Figure 5:**
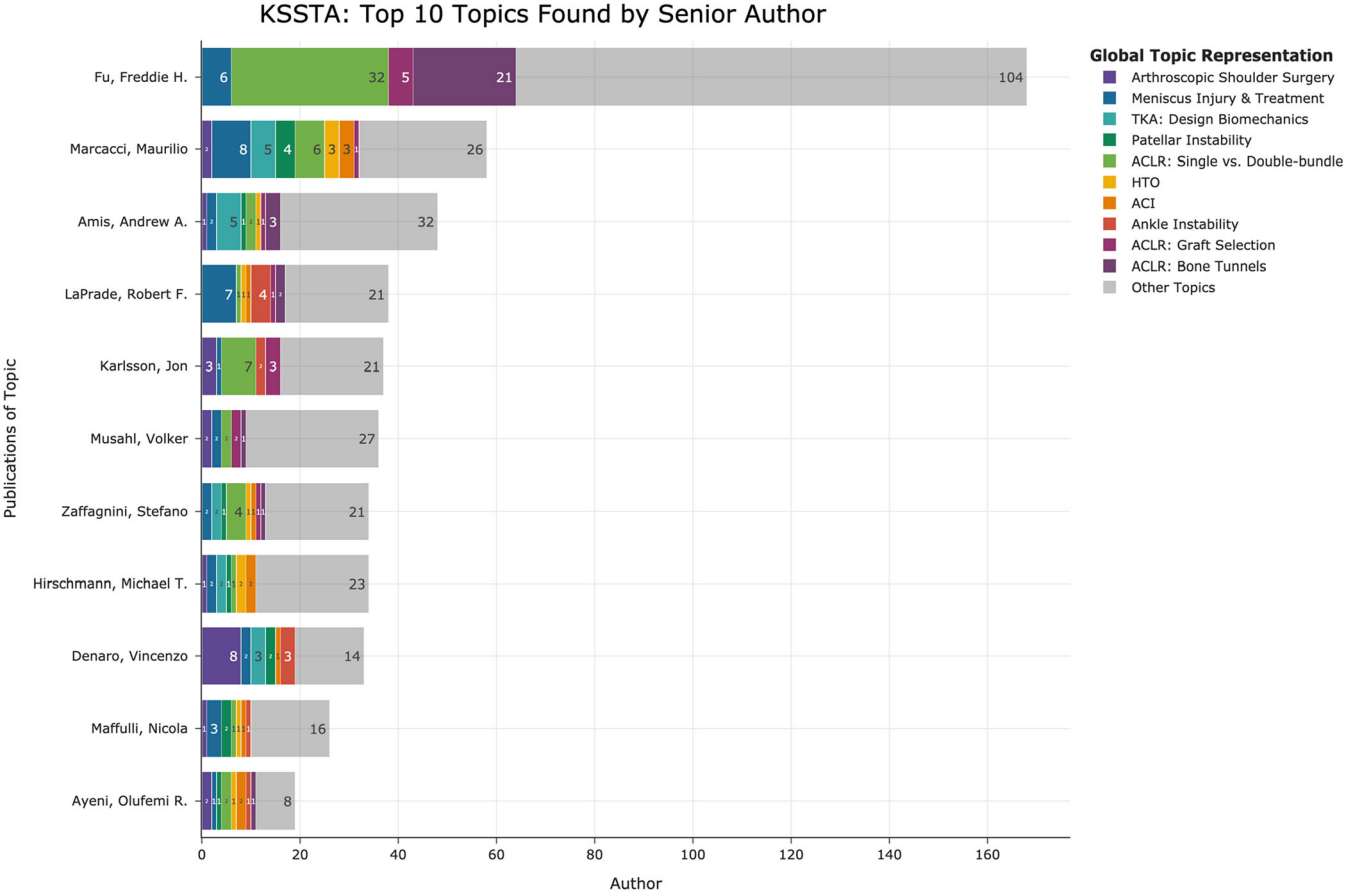
Distribution of top 10 topics among top 11 senior authors. The *y*‐axis shows the top 11 senior authors by total publications, and the *x*‐axis indicates publication counts for each senior author within the top 10 topics (coloured) and those outside these topics (grey). Purple, arthroscopic shoulder surgery; dark blue, meniscus injury and treatment; light blue, TKA: design biomechanics; dark green, patellar instability; light green, ACLR: single‐ versus double‐bundle; light orange, HTO; dark orange, ACI; red, ankle instability; dark pink, ACLR: graft selection; violet, ACLR: bone tunnels; grey, other topics. ACI, autologous chondrocyte implantation; ACLR, anterior cruciate ligament reconstruction; HTO, high tibial osteotomy; TKA, total knee arthroplasty.

**Figure 6:**
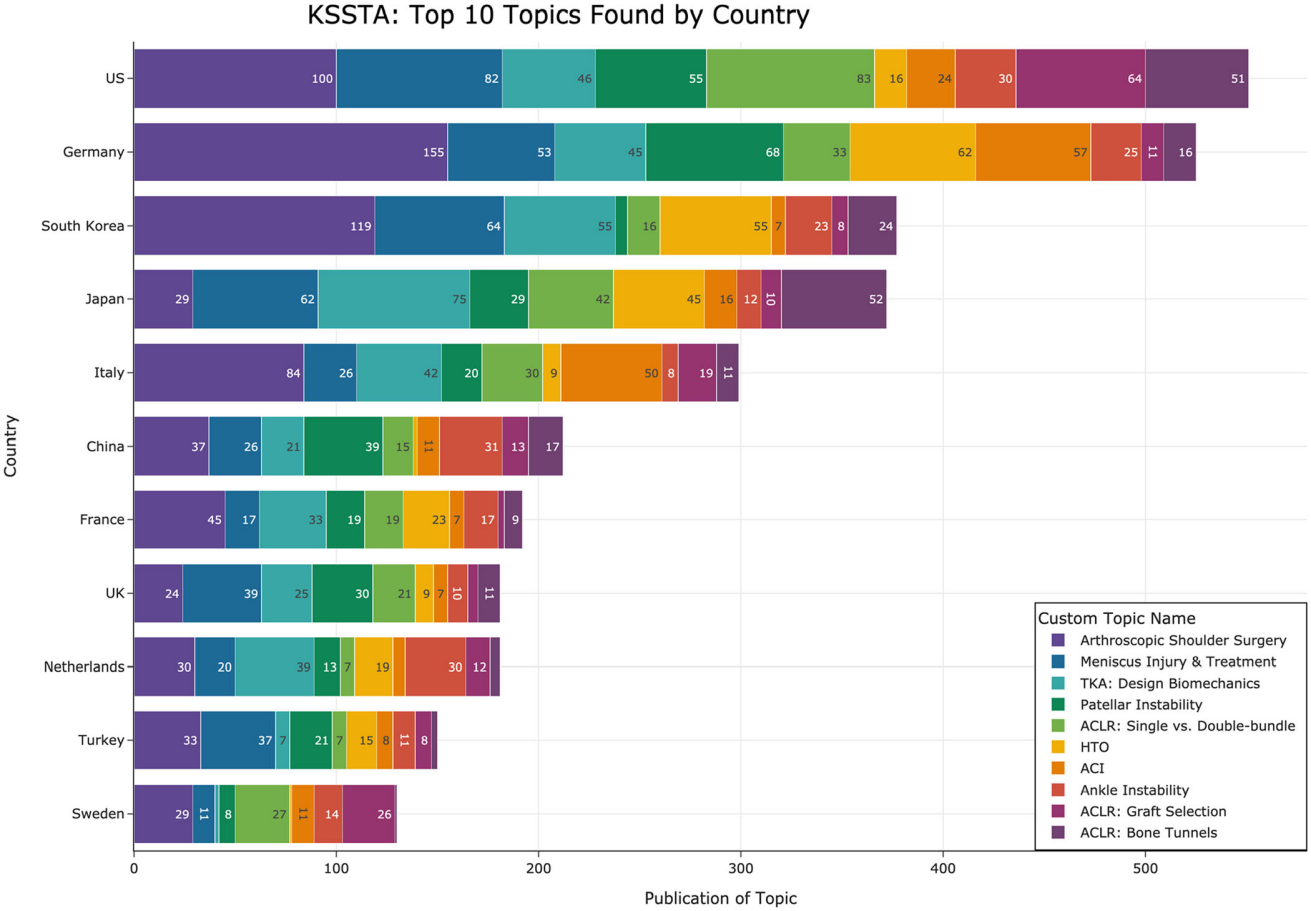
Distribution of top 10 topics by the top 11 affiliated countries of senior authors. The *y*‐axis lists the top 11 affiliated countries of senior authors, and the *x*‐axis shows publication counts. Purple, arthroscopic shoulder surgery; dark blue, meniscus injury and treatment; light blue, TKA: design biomechanics; dark green, patellar instability; light green, ACLR: single‐ versus double‐bundle; light orange, HTO; dark orange, ACI; red, ankle instability; dark pink, ACLR: graft selection; violet, ACLR: bone tunnels. ACI, autologous chondrocyte implantation; ACLR, anterior cruciate ligament reconstruction; HTO, high tibial osteotomy; TKA, total knee arthroplasty.

Figure 5 shows the distribution of the top 10 topics by the 11 most prolific senior authors (ranked by total publication count). Freddie H. Fu was the leading senior author (168 publications), while Olufemi R. Ayeni had the highest proportion (58%) of publications within the top 10 topics. Figure 6 illustrates the distribution of the 11 most common affiliated countries of senior authors across the top 10 topics, highlighting both absolute and relative proportions geographically.

**Figure 7:**
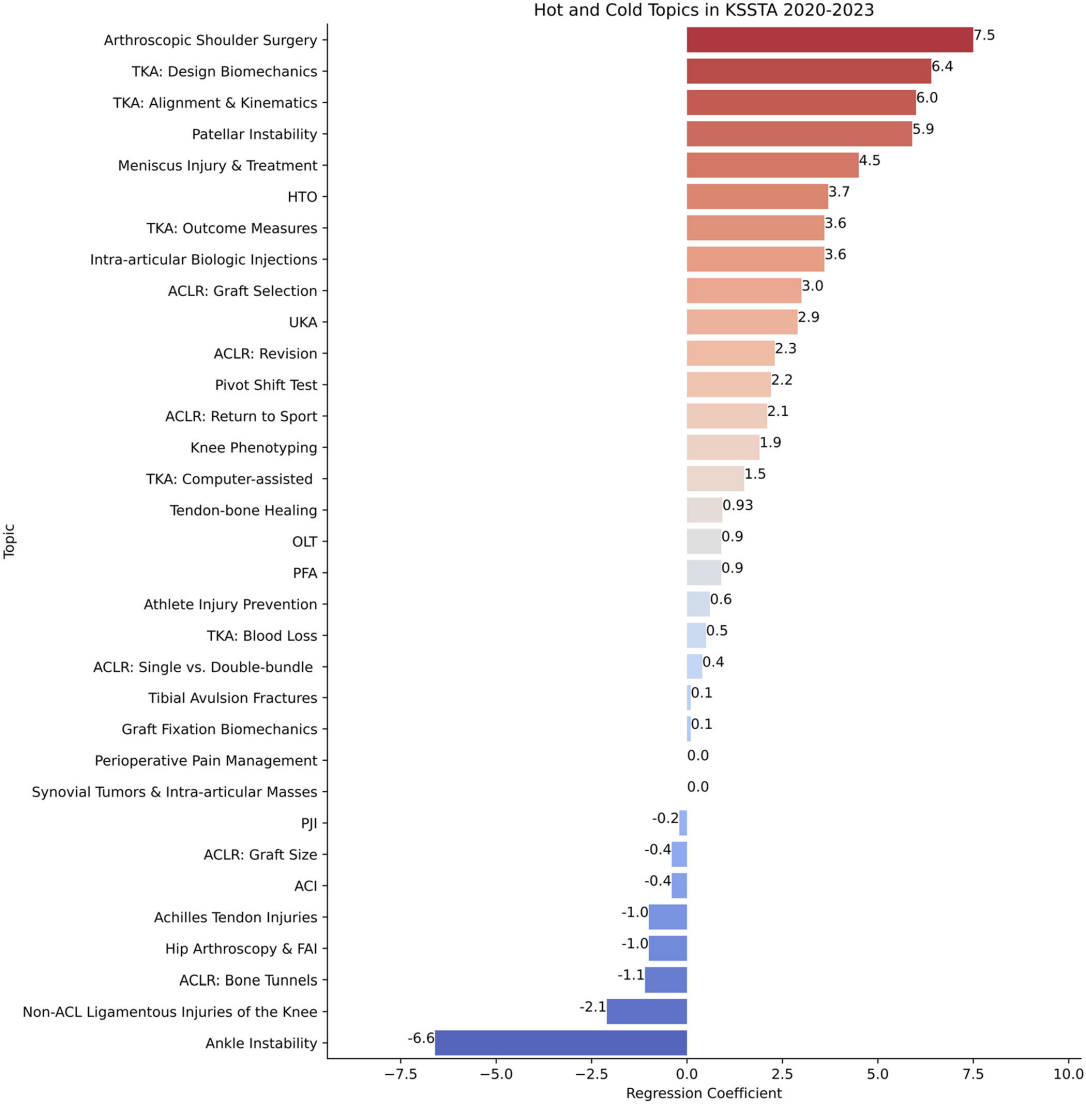
Hot and cold topics (2020–2023). The figure identifies the top 10 ‘hot’ and top 10 ‘cold’ topics from 2020 to 2023 in *KSSTA*. The *y*‐axis lists topics, while the *x*‐axis indicates the magnitude of linear regression slopes reflecting publication frequency trends. Positive slopes (red) signify increased popularity (‘hot’ topics), whereas negative slopes (blue) indicate reduced popularity (‘cold’ topics). ACI, autologous chondrocyte implantation; ACLR, anterior cruciate ligament reconstruction; FAI, femoroacetabular impingement; HTO, high tibial osteotomy; *KSSTA*, *Knee Surgery, Sports Traumatology, Arthroscopy*; OLT, osteochondral lesion of the talus; PFA, patellofemoral arthroplasty; PJI, periprosthetic joint infection; TKA, total knee arthroplasty; UKA, unicompartmental knee arthroplasty.

Figure 7 presents the linear regression results for topics. Bar lengths reflect regression slope magnitudes, and colours indicate slope direction—red for increasing (‘hot’) topics and blue for decreasing (‘cold’) topics. Between 2020 and 2023, the hottest topics were arthroscopic shoulder surgery, TKA: design biomechanics, and TKA: alignment & kinematics. Conversely, ankle instability, non‐ACL ligamentous injuries of the knee, and ACLR: bone tunnels were the coldest topics.

## DISCUSSION

Using BERTopic, this study identified 33 distinct topics within *KSSTA* and analyzed their evolution over time. Our findings affirmed the hypothesis that AI‐driven topic modelling could uncover key research trends, including increasingly popular (‘hot’) topics like arthroscopic shoulder surgery, TKA: design biomechanics, and TKA: alignment and kinematics, and declining (‘cold’) topics such as ankle instability, non‐ACL ligamentous injuries of the knee, and ACLR: bone tunnels. While bibliometric analyses offer value, our primary goal was to evaluate the feasibility and utility of BERTopic in synthesizing large volumes of orthopaedic research data.

Though NLP‐based tools have shown promise in neurosurgery for identifying evolving research themes, their application in orthopaedics remains limited. Only one prior study has used AI‐driven topic modelling in Arthroscopy [3, 10, 16, 17, 18]. In contrast to manual bibliometric reviews, which may take months or years [7, 19], BERTopic processed nearly 8000 documents in minutes. Its use of contextual language modelling—rather than simple word counts—improves clustering and thematic precision [7, 12, 14, 15, 16].

This proof‐of‐concept aligns with prior findings. For example, Khambhampati et al. [15] identified shoulder arthroscopy, biomechanics and outcome measures as leading themes post‐2014, paralleling our results. Arthroscopic shoulder surgery remains *KSSTA*'s most‐published topic, with ongoing research into rotator cuff repair, revisions, rehabilitation and return to sport [15, 23, 24, 32].

Notably, our analysis revealed TKA: design biomechanics and alignment/kinematics as up‐trending subtopics. TKA is effective for advanced osteoarthritis but has a ~20% dissatisfaction rate [19, 20]. Its rising utilization—especially among younger patients—outpaces obesity trends in the United States [21], and the global literature on TKA has surged from 483 articles in 2010 to 1083 in 2019 [11]. Prior bibliometric studies found a growing interest in perioperative management and outcome measures, with *KSSTA* playing a central role [5, 9, 11]. Emerging techniques in TKA biomechanics and alignment underscore the need for ongoing evidence‐based refinement [19, 30]. The identification of these research shifts highlights BERTopic's utility in capturing clinically relevant, evolving subthemes.

In contrast, ankle instability, non‐ACL ligamentous injuries of the knee, and ACLR: bone tunnels emerged as declining research areas. Many bone tunnel innovations occurred in the early 2000s–2010s, leading to established surgical standards and less recent investigations [6, 33]. Similarly, ankle instability treatment has seen minimal innovation [14], and research on non‐ACL knee ligament injuries remains relatively stagnant. Recognizing these ‘cold’ topics offers insight into areas of maturation and may help guide future research direction.

Ultimately, this study illustrates the potential of BERTopic to support journals and researchers by rapidly synthesizing research trends, identifying emerging areas, and uncovering knowledge gaps. As AI methods continue to evolve, their integration into orthopaedic literature surveillance may improve how we track, understand, and shape scientific progress. The findings of this study may guide the editorial strategy of *KSSTA* by highlighting which areas are receiving increasing attention, such as TKA biomechanics and alignment, and which areas—like ankle instability and ACLR bone tunnels—have seen a decline. These insights can inform future calls for submissions, shape special issue themes and help identify gaps where additional research may be warranted. Furthermore, this analysis establishes a proof‐of‐concept for using AI‐based topic modelling to periodically audit the journal's content, ensuring alignment with emerging clinical priorities and research needs.

### Limitations

This study has several limitations. First, although BERTopic effectively categorized over 7000 articles, its output was not validated against a full manual systematic review. Such a comparison would enhance the study but would defeat the purpose of using a scalable AI model. To mitigate this, two board‐certified orthopaedic surgeons independently reviewed representative titles and abstracts for each topic to ensure clinical relevance, as outlined in Appendix Table S1.

Second, AI models like BERTopic rely on the quality and scope of input data and pretrained embeddings, which may introduce biases favouring certain research styles or areas. Our reliance on Scopus title and abstract metadata—rather than full‐text content—was a practical decision, though it may have limited thematic depth in some cases. Lastly, each article was assigned a single dominant topic, which may oversimplify those with a broader scope. Future models should incorporate multi‐label classification to capture multidimensional themes more accurately. As AI‐based methods evolve, future studies should focus on cross‐journal synthesis, deeper validation frameworks, and improved interpretability to enhance the robustness of topic modelling in orthopaedic literature.

## CONCLUSIONS

This study demonstrates the efficacy of the BERTopic model in analyzing large textual data sets to identify relevant research patterns within orthopaedic literature. The results highlight BERTopic's ability to summarize thousands of articles from *KSSTA* into 33 central topics, underscoring its utility in accurately and efficiently capturing current trends and future directions in orthopaedic sports medicine research.

## AUTHOR CONTRIBUTIONS

*Conceptualization, study design and methodology*: Henry Baird and Mauricio Gallegos. *Data acquisition and curation*: Henry Baird and Mauricio Gallegos. *Data analysis and interpretation*: Henry Baird, Harris Slone and Michael Pullen. *Visualization and software application*: Henry Baird and Mauricio Gallegos. *Writing—original draft preparation*: Henry Baird, Prudhvi Kodali, Sarah Jenkins, William Newton, Harris Slone and Michael Pullen. *Writing—review and editing*: Henry Baird, Prudhvi Kodali, Sarah Jenkins, William Newton, Harris Slone and Michael Pullen. All authors have read and agreed to the published version of the manuscript.

## CONFLICT OF INTEREST STATEMENT

Harris Slone reports a relationship with Arthrex Inc., including consulting or advisory, speaking and lecture fees, and travel reimbursement. The remaining authors declare no conflicts of interest.

## ETHICS STATEMENT

IRB approval is not required as no human subjects or protected health information were involved.

## Supporting information


**Supplemental Table** 1**. Detailed Breakdown of Key Steps of BERTopic Workflow. Supplemental Table 2. Summary of 33 Distinct Topics with Top 10 Representative Terms and Three Representative Abstracts**.

## Data Availability

Data are available upon reasonable request.
