# Supplementary material for: Artificial intelligence−driven analysis identifies arthroscopic shoulder surgery, meniscus injury and treatment, and total knee arthroplasty design biomechanics as the most commonly published topics in Knee Surgery, Sports Traumatology, Arthroscopy
Source: J Exp Orthop. 2025 Jul 13;12(3):e70341. doi: 10.1002/jeo2.70341 (PMC12255955; doi:10.1002/jeo2.70341)
Supplement: Supplementary file 1 — Supplemental Table 1. Detailed Breakdown of Key Steps of BERTopic Workflow. Supplemental Table 2. Summary of 33 Distinct Topics with Top 10 Representative Terms and Three Representative Abstracts. [file JEO2-12-e70341-s001.docx]

**Supplemental Table 1. Detailed Breakdown of Key Steps of BERTopic Workflow.**

| **Step** | **Detailed Breakdown** |
| --- | --- |
| 1. Preprocessing of Datasets | A series of preprocessing steps to clean and prepare the data for further analysis were performed. Titles and abstracts for each publication were then combined into single string objects, with numbers, except those attached to strings (e.g. C5 esterase), and non-alphanumeric characters removed or converted to a Unicode equivalents (e.g. 𝛃 to B). Strings objects were lemmatized to their root form to optimize keywords relative to their topic (fractured, fractures, and fracturing become fracture). This process removes redundancy in generated representative terms for each topic while increasing specificity of each term to its respective topic. |
| 1. Embedding | String objects for each document were embedded using the sentence transformer neuml/pubmedbert-base-embeddings^1^, chosen for its superior Pearson Coefficient when analyzing biomedical documents. This process converts string object text sentences into multidimensional vectors that encapsulate the semantic information of the corresponding text in a high-dimensional space.^2^ These mathematic representations of the textual data allow for computational models to capture and use the semantic relationships between sentences using linear algebra techniques. |
| 1. Count Vectorization | Count vectorization, which converts text into a frequency matrix of tokens (i.e. words or word phrases), was then performed. The count vectorizer was modified to include tokens with n_grams of 1 to 2, allowing for one and two-word phrases like “hip” and “hip arthroscopy,” respectively, to be identified. Stop words, or tokens to be ignored during modeling, were also passed to the count vectorizer and included a standard list of 318 English words from <https://github.com/scikit-learn/scikit-learn/blob/main/sklearn/feature_extraction/_stop_words.py> that have little value for meaningful topic generation. |
| 1. Dimensionality Reduction | An important aspect of BERTopic is the dimensionality reduction of embeddings to a workable dimensional space for clustering algorithms to work with. Uniform Manifold Approximation and Projection (UMAP)^3^, a dimensionality reduction technique that is part of the standard BERTopic pipeline, was utilized. Parameter selection was based on a subjective relational analysis of clusters. Parameters of n_neightbors, n_components, and min_dist were cycled using a proprietary algorithm, and 15, 5, and 0.0 were respectively chosen for each. Because UMAP is an inherently stochastic process, a ‘fixed’ random_state of 42 was included as standard practice to generate reproducible results. |
| 1. Clustering | After reducing the dimensionality of our input embeddings, we then proceeded to cluster them into groups of similar embeddings to extract our topics. Hierarchical Density-Based Spatial Clustering of Applications with Noise (HDBSCAN)^4^, a density-based clustering algorithm that provides superior results when used for unsupervised learning, was utilized for clustering. The repository TopicTuner (<https://github.com/drob-xx/TopicTuner>) was leveraged to cycle through clustering parameters min_cluster_size and min_samples with the following goals: (1) Reduce total outliers and (2) Generate a topic number appropriate for the corpus of texts used. For final modeling, min_cluster_size of 59 and min_samples of 29 were chosen. After parameter optimization, the model was trained and saved locally. After training the model, we obtained a list of generic topics names with corresponding representative terms and abstracts, as well as the number of publications assigned to each topic. Representative terms epitomize the topic, while representative documents are the best examples of each topic. A probability threshold of 5% was utilized to identify documents that did not have at least a 5% probability of belonging to any of the identified topics. The documents that did not fit well into any of the topics identified by the model were then labeled as outliers. |
| 1. Custom Topic Naming | By default, the generic topic names generated by BERTopic include the topic number and four most representative terms concatenated together into a single string (e.g. “2_ meniscus_meniscal_tear_meniscectomy,”). To provide a more meaningful representation, custom topic names were agreed upon by two board-certified orthopaedic surgeons at our institution (e.g. “Meniscus Injury & Treatment”). For each topic, the top ten representative terms and representative abstracts were assessed to generate custom topic names (Supplemental Table 2). Custom topic names were subsequently passed back to the model for visualizations. |

1. Wolf T, Debut L, Sanh, V, et al. HuggingFace's Transformers: State-of-the-art Natural Language Processing. CoRR. 2019;abs/1910.03771. [doi:10.48550/arXiv.1910.03771](https://doi.org/10.48550/arXiv.1910.03771)
2. Basile P, Caputo A, Semeraro G. A Study on Compositional Semantics of Words in Distributional Spaces. 2012 IEEE Sixth International Conference on Semantic Computing 2012;154-161doi:[10.1109/ICSC.2012.55](https://doi.org/10.1109/ICSC.2012.55)
3. McInnes, L., Healy, J., & Melville, J. UMAP: Uniform Manifold Approximation and Projection For Dimension Reduction. arXiv. 2018;1802.03426. doi:[10.48550/arXiv.1802.03426](https://doi.org/10.48550/arXiv.1802.03426)
4. McInnes, L, Healy, J, Astels S. HDBSCAN: Heirarchical density based clustering. Journal of Open Source Software. 2017; 2(11), 205, doi:10.21105/joss.00205

**Supplemental Table 2. Summary of 33 Distinct Topics with Top 10 Representative Terms and Three Representative Abstracts**

| **Topic** | **Representative Terms** | **Abstract 1** | **Abstract 2** | **Abstract 3** |
| --- | --- | --- | --- | --- |
| Arthroscopic Shoulder Surgery | shoulder, cuff, rotator, rotator cuff, repair, arthroscopic, glenoid, cuff tear, bankart, anchor | surgical stabilization of pediatric anterior shoulder instability yield high recurrence rate a systematic review purpose the purpose of this systematic review wa to ass the surgical technique indication outcome and complication for pediatric patient year old undergoing shoulder stabilization procedure for anterior shoulder instability method the electronic database medline embase cinahl and web of science were searched from data inception to march for article addressing surgery for pediatric patient with anterior shoulder instability the methodological index for nonrandomized study minor tool wa used to ass the quality of included study result overall study with a total of patient shoulder and a mean age of year met inclusion criterion mean followup wa month the majority of study only offered shoulder stabilization procedure to patient with more than one shoulder dislocation however three study reported operating on pediatric patient after first time dislocation of the included patient had arthroscopic bankart repair had open bankart repair had modified bristow and had latarjet procedure the overall complication rate wa patient undergoing arthroscopic bankart repair experienced the highest recurrence rate of there were no significant difference in recurrent instability n or loss of external rotation n in pediatric patient treated with arthroscopic bankart repair compared to open latarjet patient had a rate of return to sport at any level ie preinjury level or any level of play postoperatively conclusion pediatric patient are at high risk of recurrent instability after surgical stabilization the majority of pediatric patient with anterior shoulder instability were treated with arthroscopic bankart repair most study recommend surgical stabilization only after more than one dislocation however given the high rate of recurrence with nonoperative management it may be reasonable to perform surgery at a firsttime dislocation particularly in those with other risk factor for recurrence with the current evidence and limited sample size it is difficult to directly compare the surgical intervention and their postoperative efficacy ie redislocation rate or range of motion there wa an overall high rate of return to sport after surgical stabilization at final followup level of evidence iv european society of sport traumatology knee surgery arthroscopy esska | arthroscopic treatment for intratendinous rotator cuff tear result in satisfactory clinical outcome and structural integrity purpose this study aimed to evaluate the clinical outcome and structural integrity of arthroscopic repair of intratendinous rotator cuff tear method patient who were diagnosed with an intratendinous tear but in whom conservative treatment failed were selected and underwent arthroscopic repair between and a total of patient men woman mean age year met the inclusion criterion and were followed up the mean followup period wa month the result were evaluated using the university of california at los angeles ucla score the society of the american shoulder and elbow surgeon rating scale as questionnaire and the visual analog scale va and range of motion rom were measured preoperatively and at final followup magnetic resonance imaging mri wa performed preoperatively and at month postoperatively postoperative mri wa performed on out of patient and analysed using the sugaya classification result corresponding to the preoperative mri finding arthroscopic finding of intratendinous tear were observed in all patient the mean active forward elevation rom wa before surgery and at the final followup the internal and external rotation at abduction were and before surgery respectively and and after surgery respectively the ucla score improved from of point preoperative to point at the final followup the as score improved from point preoperative to point postoperatively the va for pain score decreased from point preoperative to point postoperative satisfactory outcome excellentgood in term of ucla and as score were observed in of patient based on sugaya classification grade i ii and iii structural integrity were observed in and patient respectively conclusion successful clinical outcome and structural integrity can be achieved with arthroscopic repair of intratendinous rotator cuff tear involving more than half thickness therefore arthroscopic repair is a practical next treatment option for patient with intratendinous rotator cuff tear in whom conservative treatment fails level of evidence iv european society of sport traumatology knee surgery arthroscopy esska | anterior rotator cable disruption doe not affect outcome in rotator cuff tear with subscapularis involvement purpose the purpose of this study wa to compare clinical and radiological outcome after arthroscopic repair of two different rotator cuff tear configuration anterosuperior rotator cuff tear and rotator cuff tear with subscapularis involvement it wa hypothesized that although both tear configuration would show significant improvement in clinical outcome after arthroscopic repair the rotator cuff tear with subscapularis involvement where the anterior rotator cable maintains it integrity would have better clinical outcome and structural integrity method this study included patient who underwent arthroscopic repair of anterosuperior rotator cuff tear n group a and rotator cuff tear with subscapularis involvement n group b the visual analog scale va pain score subjective shoulder value ssv american shoulder and elbow surgeon as score university of california at los angeles ucla shoulder score and active range of motion rom were assessed modified belly press test wa performed to ass the strength of the subscapularis muscle cuff integrity wa evaluated using magnetic resonance arthrography or computed tomographic arthrography at month after operation result at year followup the va score ssvs as score ucla shoulder score active rom and modified belly press test showed significant improvement in both group p however these improvement showed no statistical significance between the two group on followup radiologic evaluation no significant difference in retear rate between group a of and group b of wa observed conclusion the presence of anterior cable involvement of the anterosuperior rotator cuff tear did not affect postoperative clinical outcome and retear rate compared to rotator cuff tear with subscapularis involvement where the anterior cable integrity wa maintained although the anterosuperior rotator cuff tear wa associated with more significant preoperative supraspinatus fatty infiltration therefore the present study determined that it would not be necessary to differentiate treatment protocol between these pattern level of evidence level iii european society of sport traumatology knee surgery arthroscopy esska |
| Meniscus Injury & Treatment | meniscus, meniscal, tear, meniscectomy, medial meniscus, lateral meniscus, root, extrusion, discoid, medial | nineteen percent of meniscus repair are being revised and failure frequently occur after the second postoperative year a systematic review and metaanalysis with a minimum followup of year purpose meniscus repair ha gained increasing interest over the last two decade a loss of meniscus tissue predisposes to early onset knee arthritis although there are many report of meniscus repair outcome in shortterm study data on the longterm outcome of meniscus repair are still scarce the purpose of this metaanalysis wa to evaluate the overall failure rate of meniscus repair with a minimum followup of year additionally possible factor influencing meniscus repair outcome were assessed method pubmed and scopus were searched for study of the last year reporting on meniscus repair outcome with a minimum followup of year the study wa performed following the preferred reporting item for systematic review and metaanalyses guideline the search term used for this study were meniscus or meniscal and repair title and abstract were evaluated by two author independently using meta package of r version randomeffect model were performed to pool failure rate subgroup analysis were performed and effect estimate in form of an odds ratio with ci were established result in total study with patient were included degenerative tear were excluded in two study and one study only included traumatic meniscus tear other study did not state whether the cause of meniscus tear wa degenerative or traumatic study reporting meniscus repair outcome on root repair revision anterior cruciate ligament reconstruction discoid meniscus or ramp lesion were excluded revision surgery wa used a failure definition in all included study the overall failure rate of meniscal repair at a mean followup of month wa there wa no significant difference in meniscus repair outcome when performed in combination with anterior cruciate ligament reconstruction compared to isolated meniscus repair v n or when performed on the lateral meniscus compared to the medial meniscus v n there wa no significant difference of meniscus repair outcome between verticallongitudinal tear and buckethandle tear n thirtysix percent of meniscus repair failure occur after the second postoperative year the only significant finding wa that insideout repair result in a lower failure rate compared to allinside repair v p at year conclusion the overall meniscus repair failure rate remains nineteen percent in longterm study the cause of failure is poorly documented and it remains unclear whether failure of the meniscus repair itself or additional adjacent tear lead to revision surgery despite the given technical advantage of allinside repair device this metaanalysis can not demonstrate superior outcome compared to insideout or outsidein repair at year level of evidence iv the author | nonanatomic repair of medial meniscus posterior root tear to the posterior capsule provided favourable outcome in middleaged and older patient purpose to describe a nonanatomic arthroscopic allinside repair technique for middleaged and older patient with medial meniscus posterior root tear mmprts and to evaluate the short to midterm clinical and radiologic result the hypothesis wa that this procedure would yield good clinical outcome result and structural healing in middle and olderaged patient method this wa a retrospective study evaluating patient who had undergone mmprt repair by suturing the meniscal root directly to the capsule rather than by the transtibial technique between and this allinside repair technique wa performed for patient with type ii mmprts who were over year old exclusion criterion included tibial osteotomy due to malalignment concomitant multipleligament injury and followup time le than year the lysholm score tegner activity score and international knee documentation committee ikdc score were evaluated preoperatively and at the final followup medial meniscal extrusion the international cartilage repair society icrs grade of the medial compartment and the healing status of the medial meniscus root were assessed on magnetic resonance imaging preoperatively and at the final followup result twentynine patient mean age were included the mean followup duration wa month the mean lysholm score significantly improved from preoperatively to at the final followup p the median tegner activity score improved from range to range p and the mean ikdc score improved from to p on mri case had complete healing had partial healing and had failed healing icc mean meniscal extrusion significantly increased from mm preoperatively to mm postoperatively p icc conclusion nonanatomic arthroscopic allinside repair of mmprts to the posterior capsule yielded good to excellent clinical result and a high rate of healing in the medial meniscus root on mri in middleaged and older patient at short to midterm followup despite increased meniscal extrusion this method is an alternative to the transtibial pullout repair technique for treating mmprts in middle and olderaged patient level of evidence level iv european society of sport traumatology knee surgery arthroscopy esska | icrs score worsen between year short term and year midterm followup after transtibial medial meniscus root repair despite maintained functional outcome purpose the purpose of this study wa to evaluate the midterm result of posterior medial meniscal root tear pmmrt repair through assessment of functional outcome score and magnetic resonance imaging mri method this wa a singlecenter retrospective study evaluating patient that had undergone a pmmrt this wa a followup to a previously published year outcome study all original patient were invited to participate clinical outcome included pre and postoperative international knee documentation committee ikdc and lysholm score root healing meniscal extrusion and cartilage degeneration via international cartilage repair society scale icrs grade were assessed on mri by two musculoskeletal fellowshiptrained radiologist result of the original study patient were able to participate mean age and bmi wa year and respectively with mean followup month range female the ikdc significantly increased from preoperatively to at year followup p there wa no significant change in ikdc score between year and year followup v n the lysholm also significantly increased between preoperative and year followup v p there wa no significant change between lysholm score at year and year followup v n mean extrusion did not significantly change from the preoperative state to year followup mm v mm n extrusion also did not significantly change between nd year followup mm v mm n no patient with mm of extrusion on preoperative mri had mm of extrusion on postoperative mri both medial femoral condyle and medial tibial plateau icrs grade significantly increased from preoperative to year followup p p respectively medial femoral condyle and medial tibial plateau icrs grade again significantly increased between year and year followup p p conclusion patient treated with the transtibial suture pullout technique with two locking cinch suture had maintenance of clinical outcome improvement at year followup however extrusion wa widely prevalent with worsening progression of femoral and tibial chondral disease level of evidence level european society of sport traumatology knee surgery arthroscopy esska |
| TKA: Design Biomechanics | tka, total knee, knee arthroplasty, arthroplasty, total, design, flexion, prosthesis, gap, insert | jcurve design total knee arthroplasty the posterior stabilized show wider medial pivot compared to the cruciate retaining during chair raising purpose which total knee arthroplasty tka design represents the better solution to restore a correct knee biomechanics is still debated the aim of this study wa to compare posterior stabilized p and cruciate retaining cr version of the same tka design femoral component with an anatomic sagittal radiusjcurve design by the use of dynamic roentgen stereophotogrammetric analysis rsa the hypothesis wa that the two model influence differently in vivo knee kinematic method a cohort of randomly selected patient wa evaluated month after surgery zimmer persona wa implanted eight with cr design and eight with p design the kinematic evaluation were performed using a dynamic rsa bistand drx developed in our institute during the execution of the sittostand motor task the motion parameter were obtained using the grood and suntay decomposition and the lowpoint kinematics method result p tka lateral femoral compartment had a wider anterior translation mm than the medial one mm while the two compartment of cr tka showed a similar anterior translation medial mmlateral mm t test for comparison between cr and p tka of anteroposterior translation showed a statistically significant difference p in the flexion range between and the cr prosthesis did not anteriorly translate during flexion the p design translated anteriorly showing a rollforward mechanism during extension from to of flexion and a posterior translation from to the same significant difference p between the p and cr group were found comparing the lowpoint position of the femoral condyle in the range of flexion between and for the medial compartment and between and for the lateral compartment conclusion dynamic rsa wa able to investigate for the first time in vivo the kinematic behaviour of p and cr version of the same tka jcurve design p type showed a medial pivot during sittostand motion task while the cr type showed a cylindrical movement further study are needed to evaluate the impact of different tka design on clinical result level of evidence iv european society of sport traumatology knee surgery arthroscopy esska | increased posterior translation but similar clinical outcome using ultracongruent instead of posterior stabilized total knee arthroplasty in a prospective randomized trial purpose the aim of this study wa to compare the posterior tibial translation after ultracongruent uc and posteriorstabilized p total knee arthroplasty tka with two different uc with different height in the anterior lip and two different p design this study also aimed to compare the range of motion rom and outcome score after the use of these tka model it wa hypothesised le posterior tibial translation after p than after uc tka and le posterior tibial translation with a higher anterior lip in the uc insert than with a lower one method it wa designed a a prospective randomized study of a group of patient operated with a cemented tka to clarify the main purpose of the study four group were analysed using different polyethylene design triathlon p insert in group one triathlon uc insert in group two p insert in group three and uc insert in group four one year after surgery a forced posterior drawer with a telos stress applying kg of force posteriorly on the proximal tibia at of knee flexion wa analysed in the lateral radiograph limb alignment tibial posterior slope and posterior condylar offset were also studied result patient were included in each group the average age wa year there were female and male patient there were no significant difference in any demographic or radiographic studied variable preoperative range of motion rom or preoperative knee society score ks among the different group one year after surgery the average postoperative rom and the postoperative ks knee and ks function score improved in respect of the preoperative value in all the group there were no significant difference in the postoperative outcome score among the different group p n postoperative alignment of the limb tibial posterior slope and posterior condylar offset were similar in the study group p n the postoperative posterior tibial translation wa different between group the p group group and showed significant inferior value p in respect of the uc group group and there were no difference between both group of p model but there wa a significant increase in the posterior tibial translation of the triathlon uc insert mm sd in respect of the uc insert mm sd p conclusion uc insert restrict the posterior tibial translation after tka le than p insert but a design with a high anterior lip in the polyethylene uc insert can better control the posterior tibial translation than an insert with a small anterior lip level of evidence level i randomised controlled trial european society of sport traumatology knee surgery arthroscopy esska | wider translation and rotation in posteriorstabilised mobilebearing total knee arthroplasty compared to fixedbearing both implanted with mechanical alignment a dynamic rsa study purpose the purpose of this study wa to investigate the in vivo kinematics of the same femoral design mechanically aligned posteriorstabilised p total knee arthroplasty tka with either fixedbearing fb or mobilebearing mb inlay implanted by the same surgeon using modelbased dynamic radiostereometric analysis rsa the hypothesis of the present study wa that the mb design would show wider axial rotation than the fb design without affecting the clinical outcome material and method a cohort of nonrandomised patient depuy attune psfb wa evaluated by dynamic rsa analysis at a minimum month followup while performing differently demanding daily living activity such a sit to stand sts and deep knee lunge dkl kinematic data were compared with those of a cohort of patient implanted with the same prosthetic design but with mb inlay anteriorposterior ap translation varusvalgus vv and internalexternal ie rotation of the femoral component with respect to the tibial baseplate were investigated translation of medial and lateral compartment wa analysed using the low point method according to freeman et al questionnaire to calculate objective and subjective clinical score were administered preoperatively and during followup visit by the same investigator result the fb tka design showed lower ap translation during sts mm in fb v mm in mb p lower vv rotation in fb v in mb p and lower ie rotation in fb v in mb p during dkl than the mobilebearing tka design posteriorstabilised fb group showed significant lower translation of the low point of the medial compartment than the mb group p the percentage of patient performing medial pivot in the fb group wa higher compared to mb group in the examined motor task no significant difference in postoperative range of motion for fb group and for mb group and in clinical outcome emerged between the two cohort conclusion the fb and mb design differed in ap translation vv rotation and ie rotation of the femoral component with respect to the tibial component in sts and dkl furthermore fb cohort reported a significant higher percentage of medial pivot with respect to mb cohort despite this no difference in clinical outcome were detected between group both design showed stable kinematics and represent a viable option in primary tka level of evidence prospective cohort study ii the author |
| Patellar Instability | mpfl, patellar, patellofemoral, patella, trochlear, patellar instability, tttg, mpfl reconstruction, patellar dislocation, medial patellofemoral | reconstruction of the medial patellofemoral ligament and reinforcement of the medial patellotibial ligament is an effective treatment for patellofemoral instability with patella alta purpose to evaluate the clinical outcome of the combined reconstruction of the medial patellofemoral ligament mpfl and medial patellotibial ligament mptl for patellar instability with patella alta method a total of patient underwent a combined reconstruction of the mpfl and mptl and patient were included in this study the clinical result were evaluated and compared using the international knee documentation committee ikdc score kujala score and visual analogue scale va score the tibial tuberositytrochlear groove tttg distance three index of patellar height insallsalvati ratio modified insallsalvati ratio and catondeschamps index and patellar shift and tilt were defined preoperatively and at the and month followup visit result at the and month followup visit and of the subjective outcome were excellent and were good and were fair and and were poor there were significant improvement in the ikdc score from preoperatively to p at month and p at month kujala score from preoperatively to p at month and p at month and va score from preoperatively to p at month and p at month the patellar tilt patellar shift insallsalvati ratio modified insallsalvati ratio catondeschamps index and tttg distance all decreased significantly compared with the preoperative value and there were no significant difference between the value at the and month followup conclusion the result of this study show that a combined reconstruction of the mpfl and mptl is an effective treatment for patellar instability with patella alta this article emphasizes the combined effect of mpfl and mptl instead of mpfl alone and provides an effective option for the treatment of recurrent patellar dislocation with patella alta level of evidence case series level iv european society of sport traumatology knee surgery arthroscopy esska | derotational distal femur osteotomy combined with medial patellofemoral ligament reconstruction yield satisfactory result in recurrent patellar dislocation with excessive femoral anteversion angle and trochlear dysplasia purpose the purpose of this study wa to evaluate the clinical outcome of medial patellofemoral ligament mpfl reconstruction combined with derotational distal femur osteotomy in patient with recurrent patellar dislocation who had excessive femoral anteversion angle and trochlear dysplasia method between and patient knee with recurrent patellar dislocation who had excessive femoral anteversion angle and trochlear dysplasia and were surgically treated using derotational distal femur osteotomy and mpfl reconstruction were eligible for this retrospective study these patient were assigned to two group according to the grade of trochlear dysplasia group a type a trochlear dysplasia n and group b type b c d trochlear dysplasia n preoperative and postoperative patellar tilt angle pta catondeschamps index cdi tibial tubercletrochlear groove tttg distance and femoral anteversion angle were evaluated patient outcome were assessed using the preoperative and postoperative international knee documentation committee ikdc score kujala score lysholm score tegner score and visual analog scale va score result a total of patient knee were evaluated in this study with a mean followup period of month there were no case of wound infection osteotomy site fracture deep venous thrombosis of the lower extremity or redislocation in the two group during the postoperative followup period all patient returned to full extension and flexion the postoperative tegner score lysholm score kujala score ikdc score va score pta cdi tttg distance and femoral anteversion angle were significantly improved compared with the preoperative status p there wa no significant difference between the two group n conclusion mpfl reconstruction combined with derotational distal femur osteotomy showed satisfactory clinical outcome during followup in patient with recurrent patellar dislocation who had excessive femoral anteversion angle and trochlear dysplasia even patient with highgrade trochlear dysplasia showed satisfactory result for those patient additional surgery is not necessary level of evidence level iii the author under exclusive licence to european society of sport traumatology knee surgery arthroscopy esska | anatomic reconstruction of the medial patellofemoral ligament in child and adolescent using a pedicled quadriceps tendon graft show favourable result at a minimum of year followup purpose in adult reconstruction of the medial patellofemoral ligament mpfl ha shown good result treatment for recurrent patellar instability in child and adolescent with open growth plate however requires alternative mpfl reconstruction technique this study present the outcome of a minimally invasive technique for anatomic reconstruction of the mpfl in child using a pedicled superficial quadriceps tendon graft hardwarefree patellar graft attachment and anatomic femoral fixation that spare the distal femoral physis method twentyfive consecutive patient with patellofemoral instability and open growth plate underwent anatomic reconstruction of the mpfl using a pedicled superficial quadriceps tendon graft preoperative radiographic examination included ap and lateral view to ass patella alta and limb alignment magnetic resonance imaging wa performed to evaluate trochlear dysplasia and tibial tubercletrochlear groove distance evaluation included pre and postoperative physical examination kujala score visual analog scale va and tegner activity score result the average age at the time of operation wa year the average followup after operation wa year no recurrent dislocation occurred twenty patient were very satisfied four patient were satisfied and one patient wa partially satisfied with the surgical procedure no patient wa dissatisfied the median kujala score significantly improved from preoperatively to postoperatively p and the median va score improved significantly from to p the tegner activity score increased but not significantly from preoperatively to postoperatively nonsignificant conclusion the described technique for mpfl reconstruction with a pedicled quadriceps tendon is a safe and effective technique with good clinical result and allows patient to return to sport without redislocation of the patella it might therefore be a valuable alternative to more extensive procedure in paediatric and adolescent patient level of evidence prospective study level iii european society of sport traumatology knee surgery arthroscopy esska |
| ACLR: Single vs. Double-bundle | acl, acl reconstruction, reconstruction, doublebundle, graft, anterior cruciate, sb, db, cruciate ligament, cruciate | prospective randomized comparison of anatomic single and doublebundle anterior cruciate ligament reconstruction purpose to determine if anatomic doublebundle anterior cruciate ligament acl reconstruction is superior to anatomic singlebundle reconstruction in restoring the stability and function of the knee joint method a prospective randomized clinical study wa done to compare the result of case of anatomic singlebundle acl reconstruction and case of anatomic doublebundle acl reconstruction with average followup of month tunnel placement of all the case were measured on d ct clinical result were collected after reconstruction graft appearance meniscus status and cartilage state under arthroscopy were compared and analysed too result tunnel placement confirmed with d ct were in the anatomic position a described in literature both in sb and db group no difference were found between sb and db group in clinical outcome score pivot shift test and kt measurement average sidetoside difference for anterior tibial translation wa mm in sb group and mm in db group more than of the singlebundle graft and am bundle graft in db group appeared excellent but only of pl bundle graft in db group were excellent and were in poor state no new meniscus tear wa found either in sb or db group however in db group cartilage damage in medial patellafemoral joint occurred in case this rate wa significantly higher than in the sb group which is only conclusion both single and doublebundle anatomic acl reconstruction can restore the knee stability and function very well however more incidence of poor pl status and medial patellarfemoral cartilage damage may occur in doublebundle acl reconstruction level of evidence randomized controlled trial level i springerverlag berlin heidelberg | doublebundle anterior cruciate ligament reconstruction is better than singlebundle reconstruction in term of objective assessment but not in term of subjective score purpose a comparison of clinical outcome between doublebundle db and singlebundle sb anterior cruciate ligament acl reconstruction for patient with acl injury method sixty patient were treated with either sb n or db n acl reconstruction between and the hamstring tendon were autografted with suspensory fixation on the femoral side while a bioabsorbable interference screw wa used for fixation on the tibial side these patient were evaluated using lysholm score international knee documentation committee ikdc form both objective and subjective lachman test pivot shift test and kt arthrometer result after a median followup duration of month ranging between and month the frequency of patient who had high objective ikdc score wa significantly higher in the db group than those in the sb group in term of db the lachman test wa normal in patient nearly normal in three patient and abnormal in one patient comparatively in term of sb the lachman test wa normal in patient nearly normal in eight patient and abnormal in two patient the pivot shift test wa negative in patient and patient for db and sb respectively the average k sidetoside difference wa mm for db and mm for sb the subjective ikdc and lysholm score showed nonsignificant difference between both technique conclusion doublebundle acl reconstruction wa found to have a significant advantage in anterior and rotational stability a well a objective ikdc than that of sb reconstruction however subjective measurement showed no statistical difference between the technique level of evidence ii european society of sport traumatology knee surgery arthroscopy esska | intraoperative comparison of knee kinematics of doublebundle versus singlebundle anterior cruciate ligament reconstruction purpose based on biomechanical anatomical study doublebundle reconstruction of the anterior cruciate ligament acl wa introduced to achieve better stability in the knee particularly in respect of rotatory load an in vivo computerassisted doublebundle db acl reconstruction is superior to a singlebundle sb acl reconstruction at reducing rotatory and ap laxity of the tibia at degree of knee flexion and also during the pivot shift test method the data of patient who had acl reconstruction were prospectively collected thirtytwo patient had singlebundle reconstruction sb group and received doublebundle reconstruction db group the peroperative navigation system praxim acl surgetics system helped to search for a minimal anisometry profile of the graft which wa favorable graft loosened with flexion in the anatomic area of acl insertion and preventing any conflict between the graft and the femoral notch the system also evaluated anteroposterior ap rotational stability and pivot shift the value of the pivot shift wa calculated from the value of the maximum rotation and ap translation obtained when performing the manoeuver before and after acl reconstruction comparing sb and db reconstruction result the postoperative ap displacement of the lateral compartment during the lachman test wa statistically reduced in db group in comparison with sb group mm v mm p whereas the ap displacement of the medial compartment were also reduced mm v mm p but with no statistical significance internal and external rotation at of knee flexion were lower in the db group than in sb group with statistical significance respectively v p and v p during the pivot shift test the postoperative ap maximal translation wa statistically different in both group mm in db group and mm in sb group p whereas the maximal rotation wa not statistically different in db group and in sb group n s therefore colombets index wa similar in db group and sb group respectively and n s conclusion this study show a significant intraoperative advantage in anterior and rotational stability for fourtunnel db acl reconstruction compared with sb acl reconstruction level of evidence ii springerverlag |
| HTO | osteotomy, hto, tibial osteotomy, high tibial, wedge, correction, owhto, high, openwedge, tibial | satisfactory functional and radiological outcome can be expected in young patient under year old after open wedge high tibial osteotomy in a longterm followup purpose to report the longterm outcome of medial open wedge high tibial osteotomy mowhto for the treatment of medial compartment knee osteoarthritis in patient younger than year old it wa hypothesized that the correction of knee alignment would result in preservation of knee function in a longterm followup method patient under year old who underwent mowhto for symptomatic medial compartment knee osteoarthritis between and were retrospectively reviewed after a minimum of year the osteotomy wa performed utilizing a locking plate without the use of bone graft patient were evaluated pre and postoperatively using the international knee documentation committee score the oxford knee score the knee injury osteoarthritis outcome score and the short for score standardized standing wholelimb radiograph were also obtained to ass mechanical tibiofemoral angle mtfa and the grade of osteoarthritis result a total of patient male female mean age year with a mean followup of year were included in the study during the followup period one patient required conversion to total knee replacement survival rate all clinical outcome score ikdc koos ok and s significantly improved postoperatively p with no significant deterioration over time preoperative varus alignment with an mtfa of wa corrected to immediately after surgery p and remained at the last followup furthermore no significant radiographic progression of osteoarthritis wa observed conclusion mwohto with a locking plate is an effective joint preservation method to treat medial compartment oa in active patient le than year clinical and radiological result are satisfactory and the survival rate is year after the procedure level of evidence level iv therapeutic retrospective cohort study european society of sport traumatology knee surgery arthroscopy esska | increased preoperative medial and lateral laxity is a predictor of overcorrection in open wedge high tibial osteotomy purpose this study aimed at determining whether overcorrection after open wedge high tibial osteotomy owhto would be predicted by the magnitude of preoperative medial and lateral coronal soft tissue laxity around the knee joint method overall knee of patient who underwent owhto for primary medial osteoarthritis were retrospectively reviewed the mechanical hipkneeankle hka axis weightbearing line wbl ratio medial proximal tibial angle mpta joint line obliquity coronal subluxation and joint line convergence angle jlca were measured on fullweightbearing longstanding hka radiograph preoperatively and at year postoperatively the varus valgus stress angle wa measured on preoperative radiograph the correction amount due to soft tissue factor wa calculated a the difference between the wbl ratio on postoperative year radiograph and that on virtually corrected preoperative radiograph with the same amount of mpta at year postoperatively the patient were grouped according to the presence or absence of a overcorrection of wbl ratio overcorrection or expected correction multiple logistic regression analysis wa performed to identify the preoperative risk factor of overcorrection result the average wbl ratio wa corrected from preoperatively to postoperatively p the average mpta changed from preoperatively to postoperatively resulting in an average tibia correction angle of the average estimated correction from soft tissue factor wa of the wbl ratio soft tissue correction of the wbl ratio wa confirmed in patient the preoperative jlca and valgus stress angle were significantly greater in the overcorrection group than in the expected correction group v p and v p respectively among the radiologic parameter the presence of both jlca and valgus stress angle wa the only significant risk factor for overcorrection from soft tissue factor p odds ratio conclusion the magnitude of both medial and lateral coronal soft tissue laxity wa a predictor of overcorrection from soft tissue factor after owhto overcorrection wa more likely to occur in case with both jlca and valgus stress angle level of evidence iii european society of sport traumatology knee surgery arthroscopy esska | a significant rate of tibial overcorrection with an increased jlo occurred after isolated high tibial osteotomy without considering international consensus purpose the recent esska consensus recommendation defined indication and outlined parameter for osteotomy around a degenerative varus knee the consensus collated these guideline based on the published literature available to answer commonly asked question including the importance of identifying the site and degree of the lower limb deformity in the consensus the author suggest that a knee joint line obliquity jlo greater than or a planned medial proximal tibial angle mpta preferentially indicates a double level osteotomy dlo compared to an isolated opening wedge high tibial osteotomy owhto this study aimed to analyze the correction performed on a cohort of isolated opening wedge high tibial osteotomy owhtos prior to the recent esska recommendation with a focus on the impact of knee joint line obliquity jlo and medial proximal tibial angle mpta on the choice of osteotomy procedure method this monocentric retrospective study included patient undergoing medial owhto for symptomatic isolated medial knee osteoarthritis ahlback grade i or ii and a global varus malalignment hipkneeankle angle an automated software trained to automatically detect lower limb deformity wa implemented using patient preoperative long leg alignment xrays to identify suitability for an isolated hto in knee varus deformity based on the esska recommendation the site of the osteotomy wa identified a well a the degree of correction required the esska consensus considers avoiding an isolated high tibial osteotomy if the planned resultant knee joint line orientation exceeds or mpta exceeds a preoperative abnormal mpta wa defined by a value lower than and a preoperative abnormal ldfa by a value greater than the case of dlo or dfo suggested by the software and the number of extratibial anomaly were collected multiple linear regression model were developed to establish a relationship between preoperative value and the risk of being outside of esska recommendation postoperatively result based on esska recommendation and on threshold value considered abnormal the software suggested a dlo in n of case a distal femoral osteotomy in n of case and advised against an osteotomy procedure in n of case the software detected a femoral anomaly in n of case and an jlca in n postoperatively the mpta exceeds in n and the jlo exceeds in n on multivariate analysis a high preoperative mpta wa associated with higher risk of postoperative mpta r p similarly the probability of the software advising a dlo or dfo wa associated with the presence of an normal preoperative mpta r p or an abnormal preoperative ldfa r p or a planned jlo r p conclusion analysis of patient who underwent an isolated owhto prior to the esska guideline demonstrated a significant rate of postoperative tibial overcorrection and a resultant increased jlo preoperative planning that considers the esska guideline allows for better identification of those patient requiring a dfo or dlo and avoidance of resultant postoperative deformity level of evidence iv caseseries the author under exclusive licence to european society of sport traumatology knee surgery arthroscopy esska |
| ACI | defect, cartilage, osteochondral, cartilage defect, autologous, chondrocyte, aci, autologous chondrocyte, cartilage repair, chondrocyte implantation | sustained superiority in koos subscores after matrixassociated chondrocyte implantation using spheroid compared to microfracture purpose to evaluate the safety and efficacy of matrixassociated autologous chondrocyte implantation aci using spheroid in comparison to arthroscopic microfracture for the treatment of symptomatic cartilage defect of the knee method in a prospective multicentercontrolled trial patient aged between and year with single symptomatic focal cartilage defect between and c mean median range in the knee were randomized to treatment with aci with spheroid n or microfracture n primary clinical outcome wa assessed by the knee injury and osteoarthritis outcome score koos analysis were performed in a defined hierarchical manner where outcome of aci were first compared to baseline value followed by a comparison to the microfracture group with repeatedmeasures ancova with a noninferiority approach subgroup analysis were performed to investigate the influence of age and defect size on the overall koos secondary clinical outcome were the magnetic resonance observation of cartilage repair tissue mocart modified lysholm score and international knee documentation committee ikdc examination form safety data focused on adverse event here the year result are presented at which there were observed case in the aci group and in the microfracture group result the overall koos and it five subscores were significantly improved compared to baseline for both the aci and microfracture group noninferiority of aci to microfracture wa confirmed for the overall koos and the subscores while for the subscores activity of daily living quality of life and sport and recreation of the threshold for superiority wa passed in the aci group a notably more rapid initial improvement of the koos wa found at three month for the older age group compared to the younger age group and the microfracture group no other difference were found based on age or defect size in addition clinical improvement wa found for the mocart modified lysholm and ikdc examination form both the aci and microfracture group no safety concern related to either treatment wa observed conclusion this study confirms the safety and efficacy of matrixassociated aci with spheroid at a mid to longterm followup noninferiority of aci to microfracture wa confirmed for the overall koos and all subscores while superiority wa reached for the subscores activity of daily living quality of life and sport and recreation in the aci group this underline the importance of aci for the young and active patient level of evidence i the author under exclusive licence to european society of sport traumatology knee surgery arthroscopy esska | cellfree collagen type i matrix for repair of cartilage defectsclinical and magnetic resonance imaging result purpose several welldescribed technique are available for the treatment of chondral and osteochondral defect the aim of the study wa to ass the efficacy of a singlestage procedure incorporating a new cellfree collagen type i gel for the treatment of small chondral and osteochondral defect in the knee evaluated at year followup method fifteen patient were treated with a cellfree collagen type i gel matrix of mm diameter the graft were implanted in the debrided cartilage defect and fixed by pressfit only the clinical outcome wa assessed preoperatively and at week and and month after surgery using the international knee documentation committee ikdc score tegner activity scale and visual analogue scale va graft attachment rate wa assessed week postoperatively using magnetic resonance imaging mri cartilage regeneration wa evaluated using the magnetic observation of cartilage repair tissue mocart score at and month after implantation clinical result were correlated with mri finding result six male and nine female patient were included in this study with a mean age of range no complication were reported the mean va value after week and the mean ikdc patient value after month were significantly improved from the preoperative value p and p respectively this improvement remained up to the latest followup there were no significant difference between the median preoperative and postoperative tegner value n s significant improvement of the mean mocart score wa observed after month and remained by month p mr image showed that in of the patient the graft wa completely attached by week postoperatively at month after implantation mri demonstrated complete filling in all case with a mainly smooth surface complete integration of the border zone homogenous structure of the repaired tissue and nearly normal signal intensity no correlation between any variable of the mocart score and the clinical score wa observed conclusion the present study reveals that the new method produce both good clinical and magnetic resonance imaging result use of pressfit only implanted graft of a smaller diameter lead to a high attachment rate at month followup level of evidence iv springerverlag | arthroscopic geltype autologous chondrocyte implantation present histologic evidence of regenerating hyalinelike cartilage in the knee with articular cartilage defect purpose to investigate the clinical radiological and histological result of arthroscopic geltype autologous chondrocyte implantation gaci in treating chondral defect of the knee method this study prospectively examined five male and five female with a mean age of year who underwent arthroscopic gaci between march and february the gel comprised a mixture of ml of fibrinogen plus ml of thrombin the mean size of chondral defect wa c range c international knee documentation committee ikdc subjective score knee injury and osteoarthritis outcome score koos knee society score and visual analog scale va for pain were assessed preoperatively and during regular followup examination performed for up to year postoperatively serial magnetic resonance imaging wa performed for up to year after the surgery to observe healing using the modified magnetic resonance observation of cartilage repair tissue mocart score in eight patient secondlook arthroscopy wa performed at year after the implantation to ass the status of treated cartilage and a portion of regenerated cartilage wa harvested for histologic evaluation result the mean va score p ikdc subjective score p koos pain p koos activity of daily living p and koos quality of life p showed significant improvement at year after the surgery the modified mocart evaluation showed that the score were and at week and year after the operation respectively histologic examination demonstrated a mean regenerated cartilage thickness of mm and a mean oswestry score of immunohistochemistry analysis showed that the expression of collagen type ii wa more evident and more evenly distributed than collagen type i in regenerated cartilage there wa a significant correlation between oswestry score and change in va scale from postoperative year conclusion arthroscopic gaci produce satisfactory clinical and radiologic outcome and histologic evaluation confirms sufficient regeneration of hyalinelike cartilage that correlate with improved symptom therefore it is an acceptable minimally invasive and technically simple option for the restoration of cartilage defect of the knee level of evidence iv european society of sport traumatology knee surgery arthroscopy esska |
| Ankle Instability | ankle, ankle instability, atfl, lateral ankle, sprain, cfl, chronic ankle, tibiofibular, ankle sprain, syndesmotic | anatomy of anterior talofibular ligament and calcaneofibular ligament for minimally invasive surgery a systematic review purpose to gain a better understanding of the precise anatomy of the lateral ligament of the ankle through a systematic review of published cadaveric study in order to improve anatomical minimally invasive surgery mi for treatment of chronic ankle instability cai method a systematic review of the literature wa performed using the pubmed embase cochrane database and web of science on june with the two search concept lateral ligament of the ankle and anatomy anatomical study that reported gross anatomy of the anterior talar fibular ligament atfl and calcaneal fibular ligament cfl in english were included to ass the morphology and origin and insertion of the ligament all record found in the literature search were screened by title and abstract potentially relevant article were selected for fulltext review each of the identified article wa reviewed and included in qualitative synthesis the following data were abstracted from the included article author date of publication sample size mean age the length and the width of the each ligament number of bundle of the atfl and the location and the footprint of the origin and insertion for the atfl and cfl result sixteen study were identified indicating the length of the atfl and cfl wa and mm respectively while the width wa and mm respectively fibular origin of the atfl and cfl were located on the anterior border of distal fibula at a distance of and mm proximal to the tip of the fibula respectively the talar insertion of the atfl wa located mm to the subtalar joint or mm to the anterolateral corner of the talar body the calcaneal insertion of the cfl wa located mm to the subtalar joint or mm to the peroneal tubercle on the lateral wall of calcaneus conclusion systematic review of the literature of the research for the atfl and cfl ha identified the morphology of the ligament and their location of origin and insertion this is the best available data about the atfl and cfl which will facilitate more precise anatomical mi for treatment of cai level of evidence systematic review level iv european society of sport traumatology knee surgery arthroscopy esska | arthroscopic allinside atfl and cfl repair is feasible and provides excellent result in patient with chronic ankle instability purpose chronic ankle instability ha been described a presenting with complete tear of both the anterior talofibular ligament atfl and calcaneofibular ligament cfl in of case arthroscopic technique to treat chronic ankle instability are increasingly being reported and in some instance they can be technically demanding the aim of this study wa to describe an arthroscopic allinside repair of both the atfl and cfl and to report the outcome of a group of patient with chronic ankle instability that underwent the technique method twentyfour patient male and female median age range year with chronic ankle instability and torn atfl and cfl were treated arthroscopically after failing nonoperative management median followup wa mean and range month through an arthroscopic allinside technique and using a suture passer and two knotless anchor both fascicle of the atfl and the cfl were repaired result arthroscopic examination demonstrated atfl and cfl injury in all patient subjective improvement in their ankle instability wa observed postoperatively the anterior drawer and the talar tilt test were negative at followup the median aofas score increased from mean range preoperatively to mean range at final followup conclusion chronic ankle instability with concomitant injury of both the atfl and cfl can be successfully treated by an arthroscopic allinside repair the clinical relevance of the study is the description of the first arthroscopic allinside atfl and cfl anatomic repair technique which offer excellent clinical result and the inherent benefit from minimally invasive surgery level of evidence iv retrospective case series european society of sport traumatology knee surgery arthroscopy esska | isolated injury to the lateral ankle ligament have no direct effect on syndesmotic stability purpose this study aim wa to detect the impact of lateral ankle ligament injury on syndesmotic laxity when evaluated arthroscopically in a cadaveric model the null hypothesis wa that lateral ankle ligament injury doe not affect the stability of syndesmosis method sixteen freshfrozen aboveknee amputated cadaveric specimen were divided into two group of eight specimen that underwent arthroscopic evaluation of the distal tibiofibular joint in both the group the assessment wa first done with all syndesmotic and ankle ligament intact thereafter group underwent sequential transection of the three lateral ankle ligament first to identify the effect of lateral ligament injury anterior talofibular ligament atfl calcaneofibular ligament cfl posterior talofibular ligament ptfl then followed by the syndesmotic ligament aitfl interosseous ligament iol and pitfl group underwent sequential transection of the aitfl atfl cfl iol ptfl and pitfl which represent the most commonly injured pattern in ankle sprain in all scenario four loading condition were considered under n of direct force unstressed a lateral fibular hook test anterior to posterior ap fibular translation test and posterior to anterior pa fibular translation test distal tibiofibular coronal plane diastasis at the anterior and posterior third of syndesmosis a well a ap and pa sagittal plane translation were arthroscopically measured result the distal tibiofibular joint remained stable after transection of all lateral ankle ligament atfl cfl and ptfl a well a the aitfl however after additional transection of the iol the syndesmosis became unstable in both the coronal and sagittal plane syndesmosis laxity in the coronal plane wa also observed after transection of the atfl cfl aitfl and iol subsequent transection of the pitfl precipitated syndesmosis laxity in the sagittal plane a well conclusion the finding from the present study suggest that lateral ankle ligament injury itself do not directly affect the stability of syndesmosis however if it combine with iol injury even partial injury cause syndesmotic laxity a a clinical relevance accurate diagnosis is the key for surgeon to determine syndesmosis fixation whether there is only aitfl injury or combined iol injury in concomitant acute syndesmotic and lateral ligament injury the author under exclusive licence to european society of sport traumatology knee surgery arthroscopy esska |
| ACLR: Graft Selection | hamstring, quadriceps, autograft, brace, ht, strength, muscle, graft, qt, hop | increased knee laxity with hamstring tendon autograft compared to patellar tendon autograft a cohort study of patient with primary anterior cruciate ligament reconstruction purpose to compare anterior knee laxity and patientreported outcome measure prom between anterior cruciate ligament reconstruction aclr performed with bonepatellar tendonbone bptb and hamstring tendon ht autograft and moreover to study any correlation between postoperative anterior knee laxity and prom method patient who underwent primary aclr at capio artro clinic stockholm sweden from january to october were identified in our local database instrumented laxity measurement and prom were reviewed the k arthrometer with an anterior tibial load of n wa used to evaluate knee laxity preoperatively and at the month followup the lysholm score wa collected preoperatively and at month postoperatively the knee injury and osteoarthritis outcome score koos wa collected preoperatively and at the year followup result a total of primary aclrs bptbs and ht autograft were included in the study all the patient showed a significant reduction in knee laxity from preoperatively to postoperatively bptb group from to mm ht group from to mm p for both the ht group showed a significantly increased postoperative knee laxity compared with the bptb group v mm p the mean anterior tibial translation att reduction from preoperative to postoperative wa significantly larger for the bptb graft compared with the ht graft v mm p a significantly higher rate of surgical failure defined a a postoperative sidetoside sts difference mm wa found in the ht group compared with the bptb group at followup v p a significantly larger improvement wa found in the ht group compared with the bptb group for the koos pain v p activity of daily living v p sport v p and quality of life v p subscales no significant difference regarding the mean improvement in the lysholm knee score wa found between the two graft bptb group ht group n no correlation between postoperative anterior knee laxity and prom wa found in either graft group conclusion primary aclr performed with ht autograft resulted in greater postoperative anterior knee laxity and significantly more surgical failure sts mm compared with bptb autograft the bptb autograft showed a larger anterior knee laxity reduction att reduction in conjunction with primary aclr the ht autograft led to a significantly larger improvement in four of five koos subscales from preoperatively to the year followup compared with bptb autograft there wa no association between postoperative anterior knee laxity and prom for either graft the finding of the present study provide clinician with valuable information regarding difference in knee laxity and subjective knee function between bptb and ht autograft after primary aclr the use of bptb autograft should be considered for patient with high knee stability demand level of evidence retrospective cohort study level iii the author | knee muscle strength after quadriceps tendon autograft anterior cruciate ligament reconstruction systematic review and metaanalysis purpose restoration of knee muscle strength is associated with better outcome following anterior cruciate ligament acl reconstruction but little is known about the outcome of strength following quadriceps tendon autograft qt acl reconstruction in relation to other graft type the aim of this review wa to evaluate strength outcome of the knee extensor and knee flexor following qt acl reconstruction compared to the nonreconstructed contralateral limb and alternative acl graft type method four electronic database were searched up until st february summary metaanalyses were performed comparing knee strength outcome following qt acl reconstruction to the contralateral limb by way of limb symmetry index lsi comparative metaanalyses were performed comparing qt acl reconstruction to alternative acl graft for the two most frequently reported strength outcome measure which were peak knee extensor torque lsi and peak knee flexor torque lsi at the following postoperative period month result in total study met the inclusion criterion knee strength outcome of qt acl reconstruction were included and compared to either the contralateral limb or of alternative acl graft type hamstring tendon autograft ht patellar tendon autograft pt quadriceps tendon allograft and tibialis anterior allograft knee extensor strength lsi following qt acl reconstruction did not reach even at month postoperatively conversely knee flexor strength lsi following qt acl reconstruction exceeded at the month postoperative period knee extensor strength at month following qt acl reconstruction appears similar to pt but weaker than ht acl reconstruction in addition peak knee flexor lsi wa significantly greater at month in qt acl reconstruction patient compared to ht patient conclusion the decision to utilize a qt graft for acl reconstruction should include consideration of strength outcome knee extensor strength recovery following qt acl reconstruction appears not to be restored before month level of evidence level iv european society of sport traumatology knee surgery arthroscopy esska | knee strength deficit following anterior cruciate ligament reconstruction differ between quadriceps and hamstring tendon autograft purpose to compare patient reported outcome and functional knee recovery following anterior cruciate ligament acl reconstruction using either a quadriceps tendon qt or hamstring tendon ht autograft method thirtyfive qt patient age range year participated in this study and were matched for gender age and preinjury activity level to ht age range year patient the following assessment were performed at and month postoperatively standardized patientreported outcome measure ikdc koosqol aclrsi marx activity anterior knee pain knee range of motion passive and active anterior knee laxity hop test single and triple crossover hop for distance and isokinetic strength of the knee extensor and flexor all dependent variable were analysed using a twoway mixed anova model with within time and month and betweensubject graft qt and ht factor result patient reported outcome measure and hop performance improved between and month p however no significant difference in either patientreported outcome or hop performance were found between the two graft isokinetic strength testing showed both group improved their peak knee extensor strength in the operated limb between and month p but the qt group had significantly lower knee extensor strength symmetry at both time point compared to ht at degs p and degs p in contrast the qt group had significantly greater knee flexor strength symmetry at both time point compared to ht at degs p and degs p but knee flexor strength limb symmetry did not significantly improve over time in either group conclusion recovery of knee function following either qt or ht acl reconstruction continues between and month after surgery however knee extensor strength deficit in the qt group and knee flexor strength deficit in the ht persisted at month this may have implication for decision regarding return to sport level of evidence iii european society of sport traumatology knee surgery arthroscopy esska |
| ACLR: Bone Tunnels | tunnel, femoral tunnel, tibial tunnel, femoral, bone tunnel, tunnel position, enlargement, graft, aperture, placement | no difference in graft healing or clinical outcome between transportal and outsidein technique after anterior cruciate ligament reconstruction purpose the purpose of this study wa to compare femoral tunnel geometry including tunnel position length and graft bending angle between transportal and outsidein technique in anterior cruciate ligament acl reconstruction and discover whether such difference in tunnel geometry could influence graft healing or clinical outcome method sixtyfour patient with anatomical singlebundle acl reconstruction performed with either transportal technique patient one centre or outsidein technique patient the other centre were included in this retrospective study femoral tunnel location and length and graft bending angle at the femoral tunnel were analysed on d ct knee model the location and length of the femoral tunnel and graft bending angle were compared between the two technique all patient underwent mri scan at around year following acl reconstruction it wa found that all patient had intact acl graft on mri image on oblique axial image taken after acl reconstruction to determine graft healing at femoral and tibial tunnel and the intraarticular portion graft signal intensity ratio wa calculated by dividing signal intensity si of the reconstructed acl by that of posterior cruciate ligament pcl in the region of interest selected with marosis software clinical outcome regarding tegner activity score the international knee documentation committee ikdc evaluation score lachman test and pivot shift test result were also compared between the two group result while the location of femoral tunnel wa similar to each other in both group the femoral tunnel length wa longer in the outsidein technique v mm p meanwhile the outsidein technique showed significantly more acute graft tunnel angle than the transportal technique v p however signal intensity ratio of graft compared with si of pcl were similar in femoral and tibial tunnel and intraarticular portion moreover there were no statistically significant difference in term of ikdc score v n or tegner activity score v n between the two group there wa no significant difference in measurement of lachman or pivot shift test either between the two group conclusion even though the outsidein technique in acl reconstruction created a more acute femoral graft bending angle and a longer femoral tunnel length than the transportal technique these had no negative effect on graft healing in addition transportal and outsidein technique in acl reconstruction showed similar femoral tunnel position and clinical outcome acceptable graft healing and clinical outcome can be obtained for both transportal and outsidein technique in acl reconstruction level of evidence iii european society of sport traumatology knee surgery arthroscopy esska | sagittal femoral condyle morphology correlate with femoral tunnel length in anatomical single bundle acl reconstruction purpose the purpose of this study wa to reveal the correlation between femoral tunnel length and the morphology of the femoral intercondylar notch in anatomical single bundle anterior cruciate ligament acl reconstruction using threedimensional computed tomography dct method thirty subject undergoing anatomical single bundle acl reconstruction were included in this study female male average age in the anatomical single bundle acl reconstruction the femoral and tibial tunnel were created close to the anteromedial bundle insertion site with transportal technique using postoperative threedimensional computed tomography dct accurate axial and lateral view of the femoral condyle were evaluated the correlation of femoral tunnel length which wa measured intraoperatively with the transepicondylar length tel notch width index notch outlet length the notch area axial length of blumensaats line and the height and area of the lateral wall of the femoral intercondylar notch wa statistically analyzed tunnel placement wa also evaluated using a quadrant method result the average femoral tunnel length wa mm the average tel nwi notch outlet length and the axial notch area were mm mm and m respectively the length of blumensaats line and the height and area of the lateral wall of the femoral intercondylar notch were mm mm and m respectively the length of blumensaats line the height and the area of the lateral wall of the femoral intercondylar notch were significantly correlated with femoral tunnel length femoral tunnel placement wa in a shallowdeep direction and in a highlow direction conclusion the length of blumensaats line height and area of the lateral wall of the femoral intercondylar notch are correlated with femoral tunnel length in anatomical single bundle acl reconstruction for clinical relevance these parameter are useful in predicting the length of the femoral tunnel in anatomical single bundle acl reconstruction for the prevention of extremely short femoral tunnel creation level of evidence case controlled study level iii european society of sport traumatology knee surgery arthroscopy esska | the correlation of femoral tunnel length with the height and area of the lateral wall of the femoral intercondylar notch in anatomical singlebundle acl reconstruction purpose the purpose of this study wa to reveal the correlation between femoral tunnel length and the height and area of the lateral wall of the femoral intercondylar notch in anatomical singlebundle anterior cruciate ligament acl reconstruction method twentyfour subject undergoing anatomical singlebundle acl reconstruction were included in this study female and male average age in the anatomical singlebundle acl reconstruction the femoral and tibial tunnel were created close to the anteromedial bundle insertion site using postoperative threedimensional computed tomography dct an accurate lateral view of the femoral condyle wa evaluated the correlation of femoral tunnel length which wa measured intraoperatively with the length of blumensaats line and the height and area of the lateral wall of the femoral intercondylar notch wa statistically analysed tunnel placement wa also evaluated using dct quadrant method result the average femoral tunnel length wa mm the length of blumensaats line and the height and area of the lateral wall of the femoral intercondylar notch were and m respectively both the height and the area of the lateral wall of the femoral intercondylar notch were significantly correlated with femoral tunnel length femoral tunnel placement wa in a shallowdeep direction and in a highlow direction conclusion the height and area of the lateral wall of the femoral intercondylar notch are correlated with femoral tunnel length in anatomical singlebundle acl reconstruction for clinical relevance surgeon should be careful not to make the femoral tunnel too short in knee in which the femoral intercondylar notch is low in height or small in size level of evidence casecontrolled study level iii european society of sport traumatology knee surgery arthroscopy esska |
| ACLR: Revision | aclr, acl, acl reconstruction, reconstruction, revision, consensus, injury, anterior cruciate, cruciate ligament, cruciate | management of traumatic meniscus tear the esska meniscus consensus purpose the importance of meniscus integrity in the prevention of early osteoarthritis is well known and preservation is accepted a the primary goal the purpose of the esska european society for sport traumatology knee surgery and arthroscopy european consensus on traumatic meniscus tear wa to provide recommendation for the treatment of meniscus tear based on both scientific evidence and the clinical experience of knee expert method three group of surgeon and scientist elaborated and ratified the socalled formal consensus process to define the recommendation for the management of traumatic meniscus tear a traumatic meniscus tear wa defined a a tear with an acute onset of symptom caused by a sufficient trauma the expert group included a steering group of eight european surgeon and scientist a rating group of another nineteen european surgeon and a peer review group the steering group prepared twentyseven question and answer set based on the scientific literature the quality of the answer received grade of a a high level of scientific support b scientific presumption c a low level of scientific support or d expert opinion these question and answer set were then submitted to and evaluated by the rating group all answer were scored from totally inappropriate to totally appropriate point thereafter the comment of the member of the rating group were incorporated by the steering group and the consensus wa submitted to the rating group a second time once a general consensus wa reached between the steering and rating group the finalized question and answer set were submitted for final review by the peer review group composed of representative of the esskaaffiliated national society eighteen representative replied result the review of the literature revealed a rather low scientific quality of study examining the treatment of traumatic meniscus tear of the question only one question received a grade of a a high level of scientific support and another received a grade of b scientific presumption the remaining question received grade of c and d the mean rating of all question by the rating group wa confidence interval a general agreement that mri should be performed on a systematic basis wa not achieved however mri wa recommended when arthroscopy would be considered to identify concomitant pathology in this case the indication for mri should be determined by a musculoskeletal specialist based on our data stable left in situ lateral meniscus tear appear to show a better prognosis than medial tear when repair is required surgery should be performed a early a possible evidence that biological enhancement such a needling or the application of plateletrich plasma would improve healing wa not identified preservation of the meniscus should be considered a the first line of treatment because of an inferior clinical and radiological longterm outcome after partial meniscectomy compared to meniscus repair discussion the consensus wa generated to present the best possible recommendation for the treatment of traumatic meniscus tear and provides some groundwork for a clinical decisionmaking process regarding the treatment of meniscus tear preservation of the meniscus should be the first line of treatment when possible because the clinical and radiological longterm outcome are worse after partial meniscectomy than after meniscus preservation the consensus clearly state that numerous meniscus tear that were considered irreparable should be repaired eg older tear tear in obese patient long tear etc level of evidence ii the author | revision anterior cruciate ligament reconstruction restores knee laxity but show inferior functional knee outcome compared with primary reconstruction purpose to evaluate and compare knee laxity and functional knee outcome between primary and revision anterior cruciate ligament acl reconstruction in the same cohort of patient method patient who underwent primary and revision acl reconstruction aclr at capio artro clinic stockholm sweden from to were identified in our local database inclusion criterion were same patient who underwent primary hamstring tendon ht and revision bonepatellar tendonbone bptb autograft aclr no associated ligament injury and no contralateral acl injuriesreconstructions the cause of revision aclr wa graft rupture for all patient the k arthrometer with an anterior tibial load of n wa used to evaluate knee laxity preoperatively and month postoperatively the knee injury and osteoarthritis outcome score koos wa collected preoperatively and at the year followup result a total of patient with primary and revision aclr arthrometric laxity measurement were available male mean age at primary aclr year and revision aclr year the mean preoperative and postoperative anterior sidetoside sts difference value were not significantly different between primary and revision aclr however primary aclr showed a significantly higher frequency of postoperative anterior sts difference mm compared with revision aclr v p the koos wa available for primary and revision aclr for patient male mean age at primary aclr year and revision aclr year preoperatively revision aclr showed significantly higher score in all koos subscales except for the activity of daily living adl subscale for the primary aclr the improvement from preoperatively to the year followup wa significantly greater in all koos subscales and the postoperative score were superior for pain adl and sport subscales compared with revision aclr conclusion the finding of this study showed that anterior knee laxity is restored with revision bptb autograft aclr after failed primary ht autograft aclr in the same cohort of patient however revision aclr showed a significantly inferior functional knee outcome compared with primary aclr it is important for clinician to inform and set realistic expectation for patient undergoing revision aclr patient must be aware of the fact that having revision aclr their knee function will not improve a much a with primary aclr and the final postoperative functional outcome is inferior level of evidence retrospective cohort study level iii the author | age time from injury to surgery and quadriceps strength affect the risk of revision surgery after primary acl reconstruction purpose to identify preoperative intraoperative and postoperative factor associated with revision anterior cruciate ligament reconstruction aclr within year of primary aclr method patient who underwent primary aclr at our institution from january to march were identified the primary outcome wa the occurrence of revision aclr within year of primary aclr univariate and multivariate logistic regression analysis were used to evaluate preoperative age gender body mass index bmi time from injury to surgery preinjury tegner activity level intraoperative graft type graft diameter medial meniscus mm and lateral meniscus lm resection or repair cartilage injury and postoperative sidetoside sts anterior laxity limb symmetry index lsi for quadriceps and hamstring strength and singleleghop test performance at month risk factor for revision aclr result a total of primary aclrs were included the overall incidence of revision aclr within year wa univariate analysis showed that age year bmi kg time from injury to surgery month preinjury tegner activity level lm repair sts laxity mm quadriceps strength and singleleghop test lsi of increased the odds whereas mm resection and the presence of a cartilage injury reduced the odds of revision aclr multivariate analysis revealed that revision aclr wa significantly related only to age year or ci p time from injury to surgery month or ci p and quadriceps strength lsi of or ci p conclusion age year time from injury to surgery month and month quadriceps strength lsi of increased the odds of revision aclr within year of primary aclr understanding the risk factor for revision aclr ha important implication when it come to the appropriate counseling for primary aclr in this study a large spectrum of potential risk factor for revision aclr wa analyzed in a large cohort advising patient regarding the result of an aclr should also include potential risk factor for revision surgery level of evidence iii the author |
| TKA: Outcome Measures | expectation, tka, validity, questionnaire, pain, score, hospital, arthroplasty, version, knee arthroplasty | validity and reliability of a dutch version of the foot and ankle ability measure purpose the aim of the study wa to develop a dutch language version of the foot and ankle ability measure faam and evaluate it measurement property according to the consensusbased standard for the selection of health measurement instrument cosmin definition method a forwardbackward translation procedure wa performed and subsequently the dutch version of the faam wa evaluated for it reliability and validity in patient with a variety of foot and ankle complaint the reliability wa assessed by calculating the intraclass correlation coefficient icc testretest reliability cronbachs alpha internal consistency the standard error of measurement and the minimal detectable change mdc additionally this wa done for athlete the construct validity wa assessed by the use of spearmans correlation coefficient between faam domain and similar and contradictory domain of the foot and ankle outcome score short form and the numeric rating scale for pain result the icc of the subscales ranged from to cronbachs alpha minimum wa at individual level the mdc ranged from to and at group level from to in the subgroup of athlete the reliability wa higher the hypothesized correlation of the construct validity were supported by an confirmation rate conclusion the dutch version of the faam met adequate measurement property although the reliability is not optimal the faamsport subscale is more useful in athlete and the faamsport seems not to contribute in athlete with various foot and ankle symptom the faam can be used for functional assessment and followup at group level for the general population the faam is le appropriate level of evidence diagnostic study level i european society of sport traumatology knee surgery arthroscopy esska | the german version of the highactivity arthroplasty score is valid and reliable for patient after total knee arthroplasty purpose the indication for a total knee arthroplasty tka broadened to younger and more active patient the highactivity arthroplasty score haas is a selfadministered instrument focussing on the wider range of functional ability of more active patient the haas wa developed in english and is not available in german yet this study aim to translate crosscultural adapt and ass the psychometric property of the german haas in patient month after primary tka method after forward and backward translation we examined the final version regarding it psychometric property in patient month after primary tka the haas wa sent out to patient together with routine questionnaire comprising the knee injury and osteoarthritis outcome score koos the forgotten joint score fj the euroqol eql and numerical pain rating scale acceptability reliability responsiveness content and construct validity a well a floor and ceiling effect were evaluated result fiftytwo patient were recruited the haas wa well accepted with a mean time to completion of min cronbachs alpha for internal consistency wa testretest reliability wa excellent with an intraclass correlation coefficient icc of the smallest detectable change wa good content validity wa confirmed a strong correlation wa found between the haas and koos sport r and a medium correlation for all other koos subscales r to the fj r the eql r and pain r to the haas showed no floor and ceiling effect conclusion the german version of the haas provides good validity and reliability it can be easily selfadministered and is recommended to capture highintensity activity in patient after tka level of evidence diagnostic study level i european society of sport traumatology knee surgery arthroscopy esska | unicompartmental knee arthroplasty the italian version of the forgotten joint scor is valid and reliable to ass prosthesis awareness purpose unicompartmental knee arthroplasty uka recorded an increased incidence of around per year in the united state patient experience and satisfaction after surgery were traditionally assessed by pre and postsurgical score and patientreported outcome measure prom scale traditional scale a western ontario and mcmaster university osteoarthritis index womac and oxford knee score ok reported high ceiling effect patient treated by uka usually perform well therefore it is necessary to have a prom scale with a low ceiling effect a the forgotten joint scor fj prom have to be validated in the local language to be used this study aim to perform a psychometric validation of the italian version of fj for uka for the first time method between january and october patient were included each patient completed both the fj italian version and the womac italian version in preoperative followup after week and month onth and month postoperative followup cronbachs a intraclass correlation coefficient icc standard error of measurement sem and minimal detectable change mdc were calculated to evaluate the reliability the pearson coefficient wa used to ass validity the effect size e wa used to test the responsiveness result a range of cronbachs a between and indicated good internal consistency for the fj the testretest reliability wa acceptable ie the icc wa higher than at each followup the pearson correlation coefficient between the fj and womac wa n at preoperative followup r p at month r p at month and r p at month therefore except for the preoperative period the validity of the fj score wa assessed conclusion the fj represents a valid and reliable tool with a low ceiling effect to ass the outcome improvement in uka patient therefore validating and translating this score in different language could help perform more accurate study on outcome after uka level of evidence level iii diagnostic study european society of sport traumatology knee surgery arthroscopy esska |
| Achilles Tendon Injuries | achilles, achilles tendon, tendon, tendon rupture, rupture, atrs, tendinopathy, achilles tendinopathy, midportion, atr | longer duration of operative time enhances healing metabolite and improves patient outcome after achilles tendon rupture surgery purpose the relationship between the duration of operative time dot healing response and patient outcome ha not been previously investigated an enhanced healing response related to dot may potentiate repair process especially in hypovascular and sparsely metabolized musculoskeletal tissue such a tendon this study aimed to investigate the association between dot and the metabolic healing response patientreported outcome and the rate of postoperative complication after acute achilles tendon injury method observational cohort crosssectional study with observer blinded to patient grouping a total of twohundred and fiftysix prospectively randomized patient men woman mean age year with an acute total achilles tendon rupture all operated on with uniform anaesthetic and surgical technique were retrospectively assessed at week postoperatively six metabolite were quantified using microdialysis at and month patientreported pain walking ability and physical activity were examined using selfreported questionnaire achilles tendon total rupture score foot and ankle outcome score and physical activity scale at month functional outcome wa assessed using the heelrise test complication such a deep venous thrombosis infection and reoperations were recorded throughout the study result patient who underwent longer dot exhibited higher level of glutamate p and glycerol p at week at the year followup longer dot wa associated with significantly le loss in physical activity p le pain p le walking limitation p and better functional outcome p dot did not significantly correlate with the rate of adverse event such a deep venous thrombosis infection or reruptures higher glutamate level were associated with le loss in physical activity p all correlation were confirmed by multiple linear regression taking confounding factor into consideration conclusion the result from this study suggest a previously unknown mechanism increased metabolic response associated with longer dot which may improve patient outcome after achilles tendon rupture surgery allowing for a higher amount of traumatized tissue a reflected by upregulation of glycerol in patient with longer dot may prove to be an important surgical tip for stimulation of repair of hypometabolic soft tissue injury such a achilles tendon rupture level of evidence ii the author | sclerosing injection to area of neovascularisation reduce pain in chronic achilles tendinopathy a doubleblind randomised controlled trial local injection of the sclerosing substance polidocanol ha been demonstrated to give good clinical result in a pilot study on patient with chronic achilles tendinopathy in this study consecutive patient men and woman mean age year with chronic painful midportion achilles tendinopathy were randomised to injection treatment with either polidocanol mgml group a or lidocaine hydrochloride mgml adrenaline mgml group b both substance have a local anaesthetic effect but polidocanol also ha a sclerosing effect the patient and the treating physician were blinded to the substance injected the shortterm effect were evaluated after a maximum of two treatment week apart before treatment all patient had structural tendon change and neovascularisation demonstrated with u and colour doppler under u and colour dopplerguidance the injection targeted the area of neovascularisation just outside the ventral part of the tendon for evaluation the patient recorded the severity of achilles tendon pain during tendon loading activity before and after treatment on a va patient satisfaction with treatment wa also assessed at followup mean month after a maximum of two treatment patient in group a were satisfied with the treatment and had a significantly reduced level of tendon pain p in group b no patient wa satisfied with treatment in the painfree tendon but not in the painful tendon neovascularisation wa absent after treatment after completion of the study treatment with polidocanol injection crossover in group b and additional treatment in group a resulted in and satisfied patient in group a and b respectively in summary injection with the sclerosing substance polidocanol have the potential to reduce tendon pain during activity in patient with chronic painful midportion achilles tendinopathy springerverlag | primary augmentation of percutaneous repair with flexor hallucis longus tendon for achilles tendon rupture reduces tendon elongation and may improve functional outcome purpose achilles tendon rupture atr result in loss of strength and function of the gastrosoleusachilles tendon complex probably because of gradual tendon elongation and calf muscle atrophy even after surgical repair flexor hallucis longus fhl augmentation not only reinforces the repair and provides new blood supply to the tendon but also protects the repair internally splinting the repaired achilles tendon maintaining optimal tension we prospectively compared the clinical outcome of patient with acute atr managed with either percutaneous repair only or percutaneous repair and fhl augmentation method patient with acute atr undergoing operative management were divided into two group thirty patient underwent percutaneous repair under local anesthesia and patient underwent percutaneous repair augmented by fhl tendon harvested through a cm longitudinal posteromedial incision and transferred to the calcaneus under epidural anesthesia all patient were treated by a single surgeon between and and were followed prospectively for month result the percutaneous only group wa younger than the augmented one v year p in the augmented group patient stayed overnight and only were day case whereas in the percutaneous only group patient stayed overnight and of them were day case p the duration of the procedure wa significantly longer in the augmented group v min p at month after repair the achilles tendon resting angle atra wa better in the augmented group v p a wa achilles tendon rupture score atrs v p calf circumference of the injured and the noninjured leg did not differ between the group a did the time interval to single toe raise and the time interval to walking in tiptoe although plantarflexion strength of the operated leg wa significantly weaker than the nonoperated leg in both group the difference in isometric strength of the operated leg between the group wa not significant at month v n n conclusion percutaneous repair and fhl tendon augmentation may have a place in the management of acute achilles tendon rupture reducing tendon elongation and improving functional outcome level of evidence level ii the author under exclusive licence to european society of sport traumatology knee surgery arthroscopy esska |
| UKA | uka, unicompartmental, unicompartmental knee, arthroplasty, knee arthroplasty, arthroplasty uka, oxford, ukr, revision, medial uka | a bmi above result in satisfying outcome in patient undergoing fixedbearing lateral unicompartmental knee arthroplasty purpose the purpose of this study is to analyse the effect of bmi on clinical outcome of cemented fixedbearing lateral unicompartmental knee arthroplasty uka on a to year followup method between january and january a total of lateral ukas were implanted the oxford knee score ok and the western ontario and mcmaster university osteoarthritis index for pain stiffness function and total score were administered to estimate patient overall health status pre and postoperatively result were considered good or excellent for womac value point and ok point survivorship described with kaplanmeier method wa defined a the lack of revision at the latest followup complication or further operation were recorded p value of were considered significant result one hundred one lateral ukas were assessed at a mean followup of month no patient underwent revision but patient developed aseptic loosening of the implant and year after surgery but for clinical reason neither undergo revision year survivor overall satisfaction wa generally high with excellent score in all womac subscales and ok for all bmi group considering the pain subscale womac pain patient with normal weight and overweight achieve excellent result more frequently v p than obese patient n on the other hand considering the quality of life womac qol obese patient most frequently reach excellent value even statistically significant n p conclusion although obesity ha historically been described a a contraindication to uka improved outcome with modern uka implant design have challenged this perception therefore the classic contraindication of ukas in patient with bmi kg may not be justified according to the present study lateral uka patient with bmi kg had satisfactory patientreported outcome measure compared to nonobese patient on a long term with survival rate comparable to medial uka obese patient should not be excluded from the benefit of lateral uka surgery the author under exclusive licence to european society of sport traumatology knee surgery arthroscopy esska | kinematic alignment of medial uka is safe a systematic review purpose owing to the improved understanding of knee kinematics and the successful introduction of the kinematic alignment ka technique for implanting total knee arthroplasty tka it wa recently understood that the cartier angle technique corresponds to a kinematic implantation of the unicompartmental knee arthroplasty uka component when compared to the universally spread mechanical alignment ma technique for implanting uka the ka method generates a more anatomic prosthetic knee that may be clinically advantageous the aim of this study are to determine if ka ukas are associated with acceptable functional performance and patient satisfaction question rate of residual pain and tibia plateau fracture question and rate of reoperation and revision question and to define the component orientation and limb alignment a measured on radiograph question and the stress shielding related bone loss in the proximal tibia question with ka uka and where possible to compare with ma uka study hypothesis ka uka generates good clinical outcome similar or superior to the one of ma uka method systematic review of literature database were primarily searched using healthcare database advanced search hdas two primary search were conducted using the electronic database medline embase and pubmed and a secondary search wa conducted using review article and bibliography of obtained paper in order to ascertain more material result nine eligible noncomparative prospective or retrospective cohort study which cumulated ka ukas with followup between and year fulfilled the inclusion criterion for this systematic review the finding demonstrated high knee society score ks from to and function score from to above in addition to patient satisfaction score of there wa no revision for tibia plateau fracture case for unexplained pain tibia case for component loosening and case for any cause of aseptic failure reported for ka uka the prosthetic lower limb and tibia implant alignment were both found to be in slight varus mean between and and the postoperative joint line and tibia component wa shown to be parallel to the floor when standing the ka uka component migration a measured on radiostereometry wa acceptable discussionconclusion the ka technique is an alternative personalised more physiological method for implanting uka which could be clinically advantageous when compared to the ma technique the literature support the good mid to longterm clinical safety and good efficacy of ka uka however comparison between ka and ma technique for uka wa not performed due to limited literature further investigation are needed to better define the clinical impact of ka uka and the acceptable limit for ka of the uka tibial component level of evidence level systematic review of level study european society of sport traumatology knee surgery arthroscopy esska | minimally invasive oxford medial unicompartmental knee arthroplasty in young patient purpose advanced knee arthritis in young patient is a challenging problem that may necessitate surgical treatment there are few published study of mobilebearing unicompartmental knee arthroplasty uka in young patient while indication have expanded to it use in this demanding patient group method the clinical and radiographic result of the first consecutive oxford medial ukas ouka using a minimally invasive technique phase in patient year of age or younger at the time of surgery were evaluated median age at surgery wa year kaplanmeier survivorship analysis wa used to estimate implant survival result mean time of followup evaluation wa five sd year at final followup three patient three knee had died and two patient three knee were lost to followup five knee were revised three for unexplained pain one for early infection and one for bearing fracture there wa one impending revision for progression of osteoarthritis in the lateral compartment the radiographic review demonstrated that of the knee had progressive arthritis in the lateral knee compartment of those with full joint space loss and pain the kaplanmeier survival analysis using revision for any reason a the endpoint estimated the fiveyear survival rate at ci ninetysix per cent of the nonrevised patient were satisfied with the outcome and were dissatisfied the mean oxford knee score wa sd with of the knee having a poor result the mean ak wa sd mean flexion wa sd and the mean ucla score wa sd conclusion minimally invasive oxford medial uka wa reliable and effective in this young and active patient cohort providing high patient satisfaction at midterm followup progressive arthritis in the lateral knee compartment wa a relevant failure mode in this age group most revision were performed for unexplained pain while we did not find loosening or wear in any patient level of evidence iv european society of sport traumatology knee surgery arthroscopy esska |
| Pivot Shift Test | pivot shift, pivot, shift, shift test, laxity, test, acceleration, translation, slope, knee laxity | posterior tibial plateau impaction fracture are not associated with increased knee instability a quantitative pivot shift analysis purpose this study aimed to evaluate posterolateral tibial plateau impaction fracture and how they contribute to rotatory knee laxity using quantitative pivot shift analysis it wa hypothesised that neither the presence of nor the degree of involvement of the plateau would affect rotatory knee laxity in the acldeficient knee method a retrospective review of prospectively collected data on patient with complete anterior cruciate ligament acl injury wa conducted posterolateral tibial plateau impaction fracture were identified on preoperative mri the patient were divided into two cohort fracture or no fracture the cohort with fracture wa further categorised based on fracture morphology extraarticular articularimpaction or displacedarticular fragment all data were collected during examination under anaesthesia performed immediately prior to acl reconstruction this included a standard pivot shift test graded by the examiner and quantitative data including anterior tibial translation mm via rolimeter quantitative pivot shift qps examination mm via pivot tablet technology and acceleration mse during the pivot shift test via accelerometer quantitative examination were compared with the contralateral knee result there were patient with posterolateral tibial plateau impaction fracture of these were extraarticular articularimpaction and displacedarticular regarding the two group with or without fracture there wa no difference in subjective pivot shift v respectively n qps mm v mm respectively n anterior tibial translation measurement mm v mm respectively n or acceleration of the knee during the pivot m v m respectively n when the fracture were further subdivided subgroup analysis revealed no significant difference noted in any of the measured examination between the fracture subtypes conclusion this study showed that the posterolateral tibial plateau impaction fracture are commonly encountered in the setting of acl tear however contrary to previous report they do not significantly increase rotatory knee laxity this suggests that this type of concomitant injury may not need to be addressed at the time of acl reconstruction level of evidence level iii the author under exclusive licence to european society of sport traumatology knee surgery arthroscopy esska | what it take to have a highgrade pivot shiftfocus on bony morphology purpose variation in femoral and tibial bony morphology have been associated with higher clinical grading and increased quantitative tibial translation but not tibial acceleration during the pivot shift test following anterior cruciate ligament acl injury the purpose of this study wa to determine the impact of femoral and tibial bony morphology including a measurement influenced by both parameter the lateral tibiofemoral articular distance ltad on the degree of quantitative tibial acceleration during the pivot shift test and rate of future acl injury method all patient who underwent primary acl reconstruction from to by a senior orthopedic surgeon with available quantitative tibial acceleration data were retrospectively reviewed all patient underwent a pivot shift examination under anesthesia with a triaxial accelerometer measurement of femoral and tibial bony morphology were performed by two fellowshiptrained orthopedic surgeon using preoperative magnetic resonance imaging and lateral radiograph result fiftyone patient were included at a mean followup of year the mean quantitative tibial acceleration during the pivot shift wa m range m a larger posterior condylar offset ratio r p smaller medialtolateral width of the medial tibial plateau r p lateral tibial plateau r p and lateral femoral condyle r p and a decreased ltad r p significantly correlated with increased tibial acceleration during the pivot shift linear regression analysis demonstrated an increase in tibial acceleration of m for every mm decrease in ltad nine patient sustained ipsilateral graft rupture and patient sustained contralateral acl rupture no morphologic measurement were associated with rate of future acl injury conclusion increased convexity and smaller bony morphology of the lateral femur and tibia were significantly associated with increased tibial acceleration during the pivot shift additionally a measurement termed the ltad wa found to have the strongest association with increased tibial acceleration based on the result of this study surgeon can utilize these measurement to preoperatively identify patient at risk of increased rotatory knee instability level of evidence level iv the author under exclusive licence to european society of sport traumatology knee surgery arthroscopy esska | female sex is associated with greater rotatory knee laxity in collegiate athlete purposehypothesis the purpose of this observational study wa to determine which factor including sex are associated with increased rotatory knee laxity in collegiate athlete with no history of knee injury it wa hypothesized that increased rotatory knee laxity measured by a quantitative pivot shift test would correlate with female sex increased anterior translation during the lachman test generalized ligamentous laxity and knee hyperextension method ninetyeight collegiate athlete with a median age of range year with no history of knee injury were tested ikdc and marx activity score were obtained and subject underwent measurement of anterior translation during the lachman test with a rolimeter and measurement of knee hyperextension with a goniometer for both knee a standardized pivot shift test wa performed in both knee and quantified using image analysis technology generalized ligamentous laxity wa assessed using the modified beighton score result the average anterior translation of the lateral compartment during the pivot shift test wa mm range with a mean sidetoside difference of mm range the average anterior translation during the lachman test wa range the anterior translation of the lateral compartment during the pivot shift test wa significantly higher in female median range than in male mm p anterior translation of the lateral compartment during the pivot shift test wa significantly correlated with anterior translation during the lachman test r p there wa no significant correlation between anterior translation of the lateral compartment during the pivot shift test and knee hyperextension or modified beighton score n conclusion the data from this study show that female sex is associated with increased rotatory knee laxity measured during the pivot shift test and anterior translation during the lachman test in collegiate athlete in the future these data may be helpful in diagnosing and managing acl injury in athlete and could be used in the clinic a a baseline by which to compare and identify patient who might exhibit increased rotatory laxity level of evidence diagnostic level ii european society of sport traumatology knee surgery arthroscopy esska |
| TKA: Alignment & Kinematics | femoral component, component, alignment, femoral, rotation, axis, aligned, ka, tka, kinematically | functional alignment in total knee arthroplasty best achieves balanced gap and minimal bone resection an analysis comparing mechanical kinematic and functional alignment strategy purpose key concept in total knee arthroplasty include restoration of limb alignment and softtissue balance although difference in balance have been reported amongst mechanical alignment ma kinematic alignment ka and functional alignment fa technique it remains unclear whether there are difference in gap imbalance or resection thickness when comparing different constitutional alignment subgroup method ma measured resection technique ka matched resection technique and fa technique based on the restricted ka boundary were compared in consecutive patient undergoing roboticassisted cruciateretaining total knee arthroplasty the primary outcome wa the proportion of balanced gap differential laxity mm for extension flexion medial and lateral gap measurement manual preresection laxity measurement were obtained for ma and ka and manual postresection measurement were obtained for fa in and in of knee flexion secondary outcome were resection depth and implant alignment all outcome were analysed per constitutional coronal alignment and joint line obliquity subgroup result the proportion of balance in all four gap measurement were and with ma ka and fa respectively across all constitutional alignment type fa achieved the highest proportion of balance ma resected the least amount of bone from the medial tibial plateau ka had femoral component in most valgus and most internally rotated tibial component in most varus and wa the most bonepreserving for the posteromedial femoral condyle fa had the most externally rotated femoral component and wa most bonepreserving for the distal femoral resection conclusion the study show that implant alignment to the mechanical axis or joint line anatomy equal resection alone doe not guarantee a balanced total knee arthroplasty fa resulted in the highest proportion of balanced knee across all analysed subgroup future research will consider whether one alignment philosophy lead to superior outcome for different constitutional alignment subgroup level of evidence level ii the author under exclusive licence to european society of sport traumatology knee surgery arthroscopy esska | increase in tibial force imbalance but not change in tibiofemoral laxity are caused by varusvalgus malalignment of the femoral component in kinematically aligned tka purpose the purpose of this study were to quantify the increase in tibial force imbalance ie magnitude of difference between medial and lateral tibial force and change in laxity caused by and of varusvalgus vv malalignment of the femoral component in kinematically aligned total knee arthroplasty tka and use the result to detemine sensitivity to error in making the distal femoral resection because vv malalignment would introduce the greatest change in the alignment of the articular surface at flexion the hypothesis were that the greatest increase in tibial force imbalance would occur at flexion that primarily vv laxity would significantly change at this flexion angle and that the tibial force imbalance would increase and laxity would change in proportion to the degree of vv malalignment method kinematically aligned tka wa performed on ten human cadaveric knee specimen using disposable manual instrument without soft tissue release one dprinted reference femoral component with unmodified geometry wa aligned to restore the native distal and posterior femoral joint line four dprinted femoral component with modified geometry introduced vv malalignments of and from the reference component medial and lateral tibial force were measured during passive knee flexionextension between to using a custom tibial force sensor eight laxity were measured from to flexion using a six degreeoffreedom load application system result with the tibial component kinematically aligned the increase in the tibial force imbalance from that of the reference component at of flexion wa sensitive to the degree of vv malalignment of the femoral component sensitivity were ndeg medial tibial force increasing lateral tibial force p and ndeg lateral tibial force increasing medial tibial force p for varus and valgus malalignments respectively varusvalgus malalignment did not significantly change varus internalexternal rotation anteriorposterior and compressiondistraction laxity from to flexion at only of flexion of varus malalignment increased valgus laxity p conclusion at flexion vv malalignment of the femoral component caused the tibial force imbalance to increase significantly whereas the laxity were relatively unaffected because tibial force imbalance ha the potential to adversely affect patientreported outcome and satisfaction surgeon should strive to limit error in resecting the distal femoral condyle to within mm which in turn limit the average increase in tibial force imbalance to n because laxity were generally unaffected instability resulting from large increase in laxity is not a clinical concern within the range tested level of evidence therapeutic level ii european society of sport traumatology knee surgery arthroscopy esska | deviation in femoral joint line using calipered kinematically aligned tka from virtually planned joint line are small and do not affect clinical outcome purpose kinematically aligned total knee arthroplasty ka tka strives to restore the native distal and posterior joint line of the femur because the joint line of a virtually planned femoral component on the native femur can serve a surrogate of those of the native femur the present study determined position and orientation deviation of the femoral joint line following calipered ka tka from virtually planned joint line and whether these alignment deviation affect clinical outcome our hypothesis were that the alignment deviation for most knee would be le than mm andor and that larger alignment deviation would not be associated with lower clinical outcome score method a review of lower extremity ct scanograms and ct scan of the knee identified patient treated with calipered ka tka in one limb and no other skeletal deformity in either limb d model of the operated femur with the implanted femoral component and the native femur were created the articular surface of a d model of the implanted femoral component in the tka knee were shapematched to the condyle of the native femur to create a virtual plan the shapematched femoral component served a a reference from which to determine alignment deviation of the femoral component implanted in the ipsilateral femur the forgotten joint score fjs and oxford knee score ok were obtained at an average of month result for proximaldistal and anteriorposterior position and varusvalgus and internalexternal orientation of the femoral component the root mean square deviation from the planned joint line ranged from to mm or degree the mean difference ranged from to mm or degree indicating an absence of systematic alignment deviation the proportion of knee with joint line within mm and of the joint line of virtually planned knee ranged from to for the fjs and ok the median value were out of and out of respectively and there were no significant correlation between deviation in the position and orientation and either the fjs or the ok conclusion alignment deviation were bounded by mm and for most knee which previous biomechanical study have shown reduce the risk of stiffness loss of extension loss of flexion and tibial compartment force higher than those of the native knee moreover because median fjs and ok were relatively high and because larger alignment deviation did not correlate with lower outcome score deviation did not affect clinical outcome these result validate calipered ka tka a a surgical technique which closely restores the distal and posterior femoral joint line to those planned and achieves concomitant high patientreported outcome score thus surgeon can use the calipered ka tka technique with confidence that the surgical alignment goal will be satisfied with sufficient accuracy that high patientreported outcome are achieved level of evidence iii european society of sport traumatology knee surgery arthroscopy esska |
| Hip Arthroscopy & FAI | hip, fai, hip arthroscopy, femoroacetabular, femoroacetabular impingement, impingement, labral, acetabular, alpha angle, groin | clinical outcome after revision hip arthroscopy in patient with femoroacetabular impingement syndrome fais are inferior compared to primary procedure result from the danish hip arthroscopy registry dhar purpose a many a of primary hip arthroscopy end up with a revision arthroscopy procedure when treating patient suffering from femoroacetabular impingement syndrome fais in general revision procedure are indicated because of residual impingement but only a few study present outcome data from revision hip arthroscopy after failed fais surgical treatment the purpose of this study wa to evaluate clinical outcome after revision hip arthroscopy in a fais cohort and compare outcome with a primary fais hip arthroscopy cohort and describe potential cause of failure after the primary hip arthroscopy it wa hypothesized that subjective outcome improve after revision hip arthroscopy although outcome were expected to be inferior to primary hip arthroscopic outcome method threehundred and thirtyone arthroscopic revision hip fais patient were included from the danish hip arthroscopy registry dhar patientrelated outcome measure prom copenhagen hip and groin outcome score hagos hip sport activity scale hsas eqd and numeric rating scale nrs pain were assessed in the study cohort prior to the primary procedure and at revision and at followup one year after the revision procedure these data were compared with primary hip arthroscopic fais patient result oneyear after revision surgery mean followup in month sd significant improvement p in all prom wa demonstrated but fais patient in the primary hip arthroscopic cohort demonstrated significantly higher outcome in all prom when compared at oneyear followup scar tissue residual osseous impingement and insufficient healing of the labral repair were reported a the main reason for revision surgery the conversion to total hip arthroplasty wa low conclusion revision hip arthroscopy in fais patient improves subjective outcome significantly although they are poorer than after primary fais hip arthroscopy main reason for revision arthroscopy wa scar tissue residual femoroacetabular impingement and insufficient healing of labral repair level of evidence level iii european society of sport traumatology knee surgery arthroscopy esska | clinical and radiographic predictor of failed hip arthroscopy in the management of dysplasia a systematic review and proposal for classification purpose a indication for hip arthroscopy continue to expand it efficacy in patient with more complex deformity of the hip such a those with acetabular dysplasia remains controversial the purpose of this systematic review is to identify the predictor of failed hip arthroscopy in dysplastic hip and to propose a standardize prognostic subclassification of dysplasia method this systematic review wa performed in accordance with the preferred reporting item for systematic review and metaanalyses prisma guideline three database embase pubmed and ovid medline were searched using term including hip arthroscopy and dysplasia study were screened and data extracted in duplicate study quality wa assessed using the methodological index for nonrandomized study criterion due to the nonuniform nature of study data finding were presented in descriptive summary form result thirteen study were included in this systematic review comprising dysplastic patient hip with mean age year and female most study defined hip dysplasia by a lateral centreedge angle lcea of borderline or moderate failure wa defined a progression to revision arthroscopy periacetabular osteotomy or total hip arthroplasty overall failure rate wa at an average of month following index arthroscopy smaller lcea larger tonnis angle broken shenton line and decreased joint space mm were radiographic predictor of failure severe cartilage lesion to the femoral head or acetabulum were associated with failure in five study labral debridement led to more failure than labral repair conclusion overall hip arthroscopy yielded good outcome in mildly dysplastic hip without severe chondral damage hip arthroscopy is expected to result in a failed outcome in individual with moderatetosevere hip dysplasia lcea severe cartilage lesion larger tonnis angle broken shenton line and decreased joint space mm arthroscopic surgery may be more effective in individual with borderlinetomild lcea acetabular dysplasia in the absence of severe cartilaginous lesion year survival a standardized prognostic classification of hip dysplasia based on the lcea and tonnis angle is proposed level of evidence systematic review of nonrandomized study level iv european society of sport traumatology knee surgery arthroscopy esska | modest midterm outcome after isolated arthroscopic debridement of acetabular labral tear purpose currently there is a paucity of literature regarding outcome after isolated labral debridement the purpose of this study wa to report the reoperation rate following isolated labral debridement report clinical and functional outcome after labral debridement with a minimum year followup and identify risk factor for worse clinical and functional outcome it wa hypothesized that inferior outcome are associated with an increasing tonnis grade and those with untreated femoral acetabular impingement fai method the record of patient undergoing hip arthroscopy between and were reviewed patient with labral tear who underwent isolated arthroscopic labral debridement were identified kaplanmeier estimate of failure defined a subsequent surgery wa performed for all patient patient with minimum year followup were assessed with modified harris hip score mhhs and hip outcome score ho univariate analysis wa then performed to ass which factor were associated with worse clinical and functional outcome result fiftynine hip in patient met our inclusion criterion female male with a mean age of year and mean followup of range year overall of the hip failed for repeat surgery or rating for hip function a abnormal or severely abnormal twelve hip required subsequent surgical intervention at a mean range month total hip arthroplasty open revision arthroscopic revision of the remaining hip mean mhhs wa mean ho adl score wa and mean ho sport score wa with of reporting normal or nearly normal current level of function univariate analysis revealed that hip with untreated bony impingement p or requiring concomitant chondroplasty p had inferior clinical outcome score conclusion isolated arthroscopic labral debridement for hip labral tear had combined poor result when strictly defining failure a repeat surgery or abnormal hip rating untreated fai and concomitant chondroplasty were risk factor for inferior outcome we recommend concomitant treatment for bony impingement lesion and preservation of the labrum whenever possible level of evidence retrospective case series level iv springerverlag berlin heidelberg |
| Non-ACL Ligamentous Injuries of the Knee | pcl, mcl, posterior cruciate, smcl, plc, ligament, posterior, collateral, collateral ligament, pcl reconstruction | a comparison of modified larson and anatomic posterolateral corner reconstruction in knee with combined pcl and posterolateral corner deficiency different method to reconstruct damaged posterolateral structure are available but there ha been little work studying their relative performance in combined pcl plus posterolateral corner plc deficiency we hypothesized that an anatomic reconstruction with three graft bundle crossing the joint line would restore knee laxity closer to normal than a modified twobundle larson reconstruction in a controlled laboratory study the kinematics of cadaveric knee were measured electromagnetically with posterior drawer external rotation or varus rotation load applied with the knee at sequential stage intact pcldeficient pcl plus plcdeficient modified larson reconstruction anatomic plc reconstruction the graft bundle were tensioned sequentially to restore specific degree of freedom to intact value of laxity at specific angle of knee flexion a significant difference wa not found between the two reconstruction both reconstruction restored external rotation and varus laxity to normal both restored posterior drawer to that caused by isolated pcl deficiency but did not restore posterior laxity to normal it wa concluded that with appropriate graft tensioning both plc reconstruction could restore both external rotation and varus laxity to normal but not posterior drawer the threestranded anatomical reconstruction did not perform better than the modified twostrand larson technique both of these isolated plc reconstruction in knee with combined pcl plus plc deficiency restored the knee to the laxity condition of an isolated pcldeficiency they could not reduce posterior drawer to normal springerverlag | medial collateral ligament reconstruction graft isometry is effected by femoral position more than tibial position purpose the purpose of this study wa to examine the length change pattern of the native medial structure of the knee and determine the effect on graft length change pattern for different tibial and femoral attachment point for previously described medial reconstruction method eight cadaveric knee specimen were prepared by removing the skin and subcutaneous fat the sartorius fascia wa divided to allow clear identification of the medial ligamentous structure knee were then mounted in a custommade rig and the quadriceps muscle and the iliotibial tract were loaded using cable and hanging weight thread were mounted between tibial and femoral pin positioned in the anterior middle and posterior part of the attachment site of the native superficial medial collateral ligament smcl and posterior oblique ligament pol pin were also placed at the attachment site relating to two commonly used medial reconstruction bosworthlind and laprade length change between the tibiofemoral pin combination were measured using a rotary encoder a the knee wa flexed through an arc of result with knee flexion the anterior fibre of the smcl tightened increased in length whilst the posterior fibre slackened decreased in length all fibre region of the pol displayed a uniform lengthening of approximately between and knee flexion the most isometric tibiofemoral combination wa between pin placed representing the middle fibre of the smcl length change with knee flexion the simulated smcl reconstruction that produced the least length change wa the lindbosworth reconstruction with the tibial attachment at the insertion of the semitendinosus and the femoral attachment in the posterior part of the native smcl attachment side this appeared more isometric than using the attachment position described for the laprade reconstruction conclusion the complex behaviour of the native mcl could not be imitated by a single pointtopoint combination and surgeon should be aware that small change in the femoral mcl graft attachment position will significantly effect graft length change pattern reconstructing the smcl with a semitendinosus autograft left attached distally to it tibial insertion would appear to have a minimal effect on length change compared to detaching it and using the native tibial attachment site a pol graft must always be tensioned near extension to avoid capturing the knee or graft failure the author | the medial structure of the knee have a significant contribution to posteromedial rotational laxity control in the pcldeficient knee purpose various reconstruction technique have been employed to restore normal kinematics to pcldeficient knee however study show that failure rate are still high damage to secondary ligamentous stabilizer of the joint which commonly occurs concurrently with pcl injury may contribute to these failure the main objective of this study wa to quantify the biomechanical contribution of the deep medial collateral ligament dmcl and posterior oblique ligament pol in stabilizing the pcldeficient knee using a joint motion simulator method eight cadaveric knee underwent biomechanical analysis of posteromedial stability and rotatory laxity using an amti vivo joint motion simulator combined posterior force n and internal torque nm load followed by pure internalexternal torque nm were applied at and of flexion the specimen were tested in the intact state followed by sequential sectioning of the pcl dmcl pol and smcl the order of sectioning of the dmcl and pol wa randomized providing n for each cutting sequence change in posteromedial displacement and rotatory laxity were measured a were the biomechanical contribution of the dmcl pol and smcl in resisting these load in a pcldeficient knee result overall it wa observed that pol transection caused increased posteromedial displacement and internal rotation in extension whereas dmcl transection had le of an effect in extension and more of an effect in flexion although statistically significant difference were identified during most loading scenario the increase in posteromedial displacement and rotatory laxity due to transection of the pol or dmcl were usually small however when internal torque wa applied to the pcldeficient knee the combined torque contribution of the dmcl and pol towards resisting rotation wa similar to that of the smcl conclusion the dmcl and pol are both important secondary stabilizer to posteromedial translation in the pcldeficient knee with alternating role depending on flexion angle thus in a pcldeficient knee concomitant injury to either the pol or dmcl should be addressed with the aim of reducing the risk of pcl reconstruction failure european society of sport traumatology knee surgery arthroscopy esska |
| Athlete Injury Prevention | player, injury, football, skier, team, season, acl injury, elite, prevention, soccer | injury in norwegian female elite soccer a prospective oneseason cohort study female soccer ha become increasingly popular during the last two decade according to the international football association fifa there are approximately million registered female soccer player in the world three study in elite soccer have shown an injury incidence during game ranging from to injury per h a very high incidence of aclinjuries ranging from to per game hour ha also been shown we followed the norwegian female elite series during the season to estimate the incidence and characteristic of injury a total of female soccer player on ten team were followed during the elite season in norway we recorded baseline data match and training exposure and injury data a type of injury location and severity of injury the mean age of the player wa year range a total of injury were recorded and of these were overuse injury occurred during game and during training session the incidence of acute injury wa per game hour and per training hour the majority of the injury occurred in the lower extremity but there were also a significant number of head injury the most common injury type wa ankle sprain half of the injury were minor with training or game absence of le than day midfielders sustained the most injury with an incidence of per game hour we recorded two aclinjuries and two pclinjuries during the season they all occurred during game and the incidence wa therefore calculated to per game hour for both injury type the incidence of injury reported for female soccer varies considerably with the highest number reported from germany and the present study these study have also the highest incidence of minor injury registered the location of the injury is quite similar compared to other report but the number of ankle sprain seems to be higher in our study whereas the number of knee and thigh injury is lower there ha been much attention to acl injury in team handball and hamstring injury in soccer in norway and this could have influenced the team preseason training resulting in a reduction in the incidence of these injury type the high number of ankle injury ha to be addressed to see whether this is a result of inadequate rehabilitation routine leading to reinjuries or other factor the high number of aclinjuries in these report is alarming and need special attention in the future springerverlag | prevention of noncontact anterior cruciate ligament injury in soccer player part a review of prevention program aimed to modify risk factor and to reduce injury rate soccer is the most commonly played sport in the world with an estimated million active soccer player participating in the game a on inherent to this sport is the higher risk of injury to the anterior cruciate ligament acl relative to other sport acl injury cause a significant loss of time from competition in soccer which ha served a the strong impetus to conduct research that focus to determine the risk factor for injury and more importantly to identify and teach technique to reduce this injury in the sport this research emphasis ha afforded a rapid influx of literature aimed to report the effect of neuromuscular training on the risk factor and the incidence of noncontact acl injury in highrisk soccer population the purpose of the current review is to sequence the most recent literature relating the effect of prevention program that were developed to alter risk factor associated with noncontact acl injury and to reduce the rate of noncontact acl injury in soccer player to date there is no standardized intervention program established for soccer to prevent noncontact acl injury multicomponent program show better result than singlecomponent preventive program to reduce the risk and incidence of noncontact acl injury in soccer player lower extremity plyometrics dynamic balance and strength stretching body awareness and decisionmaking and targeted core and trunk control appear to be successful training component to reduce noncontact acl injury risk factor decrease landing force decrease varusvalgus moment and increase effective muscle activation and prevent noncontact acl injury in soccer player especially in female athlete preseason injury prevention combined with an inseason maintenance program may be advocated to prevent injury compliance may in fact be the limiting factor to the overall success of acl injury intervention targeted to soccer player regardless of gender thus interventional research must also consider technique to improve compliance especially at the elite level which will likely influence trickle down effect to subelite level future research is also needed for male soccer athlete to help determine the most effective intervention to reduce the noncontact acl injury risk factor and to prevent noncontact acl injury springerverlag | prevention of noncontact anterior cruciate ligament injury in soccer player part mechanism of injury and underlying risk factor soccer is the most commonly played sport in the world with an estimated million active soccer player by inherent to this sport is the higher risk of injury to the anterior cruciate ligament acl relative to other sport acl injury cause the most time lost from competition in soccer which ha influenced a strong research focus to determine the risk factor for injury this research emphasis ha afforded a rapid influx of literature defining potential modifiable and nonmodifiable risk factor that increase the risk of injury the purpose of the current review is to sequence the most recent literature that report potential mechanism and risk factor for noncontact acl injury in soccer player most acl tear in soccer player are noncontact in nature common playing situation precluding a noncontact acl injury include change of direction or cutting maneuver combined with deceleration landing from a jump in or near full extension and pivoting with knee near full extension and a planted foot the most common noncontact acl injury mechanism include a deceleration task with high knee internal extension torque with or without perturbation combined with dynamic valgus rotation with the body weight shifted over the injured leg and the plantar surface of the foot fixed flat on the playing surface potential extrinsic noncontact acl injury risk factor include dry weather and surface and artificial surface instead of natural grass commonly purported intrinsic risk factor include generalized and specific knee joint laxity small and narrow intercondylar notch width ratio of notch width to the diameter and cross sectional area of the acl preovulatory phase of menstrual cycle in female not using oral contraceptive decreased relative to quadriceps hamstring strength and recruitment muscular fatigue by altering neuromuscular control decreased core strength and proprioception low trunk hip and knee flexion angle and high dorsiflexion of the ankle when performing sport task lateral trunk displacement and hip adduction combined with increased knee abduction moment dynamic knee valgus and increased hip internal rotation and tibial external rotation with or without foot pronation the identified mechanism and risk factor for noncontact acl injury have been mainly studied in female soccer player thus further research in male player is warranted noncontact acl injury in soccer player likely ha a multifactorial etiology the identification of those athlete at increased risk may be a salient first step before designing and implementing specific preseason and inseason training program aimed to modify the identified risk factor and to decrease acl injury rate current evidence indicates that this crucial step to prevent acl injury is the only option to effectively prevent the sequela of osteoarthritis associated with this traumatic injury springerverlag |
| ACLR: Graft Size | notch, bundle, acl, insertion, intercondylar, intercondylar notch, footprint, width, insertion site, pl bundle | size correlation between the tibial anterior cruciate ligament footprint and the tibia plateau purpose the purpose of this study wa to reveal the correlation between the size of the native anterior cruciate ligament acl footprint and the size of the tibia plateau method twentyfour nonpaired human cadaver knee were used all soft tissue around the knee were resected except the acl the acl wa cut in the middle and the femoral bone wa cut at the most proximal point of the femoral notch the acl wa carefully dissected and the periphery of the acl insertion site wa outlined on both the femoral and tibial side an accurate lateral view of the femoral condyle and the tibial plateau wa photographed with a digital camera and the image were downloaded to a personal computer the size of the femoral and tibial acl footprint and anteriorposterior ap and mediallateral ml length of the tibia plateau and area of tibia plateau were measured with image j software national institution of health result the size of the native femoral and tibial acl footprint were and m respectively the ap length of the whole medial and lateral facet of the tibia plateau were a follows and mm respectively the ml length of the tibia plateau wa mm total area of tibia plateau wa m the ap length of the lateral facet of the tibia plateau pearsons correlation coefficient p and the total area of tibia plateau pearsons correlation coefficient p were significantly correlated with the size of the tibial acl footprint conclusion for clinical relevance the ap length of lateral facet of the tibia plateau and total area of tibia plateau are significantly correlated with the size of the tibial acl footprint it might be possible to predict the size of the acl measuring these parameter springerverlag berlin heidelberg | acl footprint size is correlated with the height and area of the lateral wall of femoral intercondylar notch purpose the purpose of this study wa to reveal the correlation between the size of the native anterior cruciate ligament acl footprint and the size of the lateral wall of femoral intercondylar notch method eighteen nonpaired human cadaver knee were used all soft tissue around the knee were resected except the acl the acl wa cut in the middle and the femoral bone wa cut at the most proximal point of the femoral notch the acl wa carefully dissected and the periphery of the acl insertion site wa outlined on both the femoral and tibial side an accurate lateral view of the femoral condyle and the tibial plateau wa photographed with a digital camera and the image were downloaded to a personal computer the size of the femoral and tibial acl footprint length of blumensaats line and the height and area of the lateral wall of femoral intercondylar notch were measured with image j software national institution of health result the size of the native femoral and tibial acl footprint were and m respectively the length of blumensaats line and the height and area of the lateral wall of femoral intercondylar notch were mm mm and m respectively both the height and the area of the lateral wall of femoral intercondylar notch were significantly correlated with the size of the acl footprint on both the femoral and tibial side conclusion for clinical relevance the height and area of the lateral wall of femoral intercondylar notch can be a predictor of native acl size prior to surgery however the length of blumensaats line showed no significant correlation with native acl size springerverlag | proportional evaluation of anterior cruciate ligament footprint size and knee bony morphology purpose the purpose of this study wa to reveal the correlation in size between the native anterior cruciate ligament acl footprint and the femoral intercondylar notch and the tibia plateau and to calculate the proportion in size between the acl footprint and knee bony morphology method twentysix nonpaired human cadaver knee were used all soft tissue around the knee were resected except the acl the acl wa cut in the middle and the femoral bone wa cut at the most proximal point of the femoral notch the acl wa carefully dissected and the periphery of the acl insertion site wa outlined on both the femoral and tibial side an accurate lateral view of the femoral condyle and an axial view of the tibial plateau were photographed with a digital camera and the image were downloaded to a personal computer the size of the femoral and tibial acl footprint and the area of the lateral wall of the intercondylar notch and the tibia plateau were measured with image j software national institution of health result the size of the native femoral and tibial acl footprint were and m respectively the area of the lateral wall of the intercondylar notch and the tibia plateau were and m respectively the femoral acl footprint area and the area of the lateral wall of the femoral intercondylar notch pearsons correlation coefficient p and the tibial acl footprint area and the area of the tibia plateau pearsons correlation coefficient p both showed significant correlation the femoral acl footprint wa the size of the lateral wall of the femoral intercondylar notch and the tibial acl footprint wa the size of the tibia plateau conclusion for clinical relevance the femoral acl footprint is approximately the size of the intercondylar notch and the tibial acl footprint is approximately the size of the tibia plateau it might be possible to predict the size of the acl measuring these parameter preoperatively springerverlag berlin heidelberg |
| Graft Fixation Biomechanics | screw, fixation, interference, interference screw, graft, screw fixation, bioabsorbable, load, pin, device | biomechanical evaluation of a medial knee reconstruction with comparison of bioabsorbable interference screw construct and optimization with a cortical button current fixation technique in medial knee reconstruction predominantly utilize interference screw alone for soft tissue graft fixation the use of concurrent fixation technique a part of a hybrid fixation technique ha also been suggested to strengthen soft tissue fixation although these hybrid fixation technique have not been biomechanically validated the purpose wa to biomechanically evaluate two distal tibial superficial mcl graft fixation technique that consisted of an interference screw alone and in combination with a cortical button furthermore the aim wa to compare interference screw of different construct twentyfour porcine tibia average bone mineral density of gc range gc measured by dexa scan were divided into group of six specimen each group ia consisted of a mm polyllactide plla interference screw group ib utilized a plla interference screw in combination with a cortical button group iia consisted of a mm composite polyllactidecod llactide and biphasic calcium phosphate bcp interference screw group iib also utilized a composite interference screw in combination with a cortical button the specimen were biomechanically tested with cyclic cycle n hz and loadtofailure mmmin parameter during cyclic loading a significant increase in stiffness wa seen for the plla hybrid nmm fixation compared to the plla screwonly nmm group p failure load were n for the composite screw n for the plla screwonly n for the composite hybrid fixation and n for the plla hybrid fixation the plla screw alone wa found to provide adequate fixation for a superficial mcl reconstruction and the use of a cortical suture button combined with the plla screw resulted in a stiffer fixation during cyclic loading the current reconstruction superficial mcl graft fixation technique utilizing a plla interference screw alone serf a an adequate recreation of the native tibial superficial mcl strength in addition a hybrid fixation with a cortical button which lends additional cyclic stiffness to it fixation would be advisable for use in suboptimal fixation case springerverlag | tibial fixation comparison of semitendinosusbone composite allograft fixed with bioabsorbable screw and bonepatella tendonbone graft fixed with titanium screw tibial fixation remains the weak link of acl reconstruction over the first week postoperatively this study compared the biomechanical property of tibial fixation for a bonepatellar tendonbone bptb graft and a novel semitendinosusbone composite sbc allograft with mixed corticalcancellous bone dowel at each end seven paired fresh frozen cadaveric knee year were stripped of all soft tissue attachment and randomly assigned to receive either the bptb graft or sbc allograft graft were placed into tibial tunnel via a standard protocol and secured with either a m mm bioabsorbable sbc or titanium bptb screw graft were cycled ten time in a servo hydraulic device from n prior to pull to failure testing at a rate of mmmin with the force vector aligned with the tibial tunnel worst case scenario wilcoxon signed rank test were used to evaluate biomechanical difference between graft type tibial bone mineral density and interference screw insertion torque were statistically equivalent between graft type the mode of failure for all construct wa direct screw and graft construct pullout from the tibial tunnel significant difference were not observed between graft type for maximum load at failure strength bptb n v sb n or stiffness bptb nm nmm v sbc nmm the sbc allograft yielded significantly more displacement prior to failure than the bptb graft mm v mm increased construct displacement appeared to be due to fixation failure with some evidence of graft tissue tearing around the suture bioabsorbable screw mm fixation of the sbc allograft produced unacceptable displacement level during testing further study is recommended using a titanium interference screw or a longer bioabsorbable screw for sbc graft fixation under cyclic loading condition springerverlag | pullout strength of tibial graft fixation in anterior cruciate ligament replacement with a patellar tendon graft interference screw versus staple fixation in human knee the endoscopic single incision technique for anterior cruciate ligament acl reconstruction with a femoral halftunnel may lead to a grafttunnel mismatch and subsequent protrusion of the block from the tibial tunnel the typical tibial fixation with an interference screw is not possible in these case fixation with staple in a bony groove inferior to the tunnel outlet can be used a an alternative technique current literature doe not provide biomechanical data of either fixation technique in a human model this study wa performed to evaluate the primary biomechanical parameter of this technique compared with a standard interference screw fixation of the block fiftyfive freshfrozen relatively young mean age year human cadaver knee joint were used graft were harvested from the patellar tendon midportion with bone block of mm length and mm width a mm tibial tunnel wa drilled from the anteromedial cortex to the center of the tibial insertion of the acl three different size of interference screw mm were chosen a a standard control procedure n for tibial boneblock fixation the graft wa placed through the tunnel and the screw wa then inserted on the cancellous or the cortical surface respectively fifteen knee were treated by staple fixation a groove wa created inferior to the tunnel outlet with a chisel the bone block wa fixed in this groove with two barbed stainless steel staple tensile testing in both group wa carried out under an axial load parallel to the tibial tunnel in a zwick testing machine with a velocity of mm dislocation of the graft and stiffness were calculated at n load maximum load to failure using interference screw varied between and n load to failure using staple wa n dislocation of the graft ranged between and mm for interference screw fixation and wa mm for staple stiffness calculated at n load wa significantly higher in staple fixation with either fixation technique the recorded failure load were sufficient to withstand the graft load which are to be expected during the rehabilitation period staple fixation of the bone block outside of the tunnel resulted in a fixation strength comparable to interference screw fixation |
| Tibial Avulsion Fractures | fracture, avulsion fracture, avulsion, plateau fracture, stress fracture, eminence, plateau, tibial plateau, tibial eminence, eminence fracture | clinical outcome of arthroscopic reduction and suture for displaced acute and chronic tibial spine fracture this paper report the clinical outcome of the arthroscopic reduction and pullout suture technique in acute and chronic displaced tibial spine anterior cruciate ligament acl avulsion fracture between april and december patient received an arthroscopic reduction and pullout suturing of displaced tibial spine fracture acl avulsion fracture of tibia of case ten were acute fracture and four were chronic nonunion fracture in which all patient showed extension limitation the mean followup period wa month ranging from to month at final followup review of range of motion lachman test anterior drawer test k arthrometer lysholm knee score and hospital for special surgery hs score were evaluated compared to conventional pullout suturing several key modification to surgical technique were used in all patient radiological bony union wa detected at mean week range week after surgery all patient were able to return to their preinjury activity and sport level at final followup full range of motion wa achieved in all patient anterior draw test lachman test and k le than mm sidetoside were all negative in patient one female patient who wa year old at the time of surgery complained of no subjective instability but showed lachman grade i and mm sidetoside difference in k she also revealed difference of genu recurvatum deformity two child including the previouslymentioned yearold female patient showed leglength discrepancy of cmthe affected leg being longerat final followup the mean lysholm knee score were range and hs knee score were range arthroscopic reduction with modified pullout suturing technique in displaced tibial spine acl avulsion fracture showed excellent union rate for both acute and chronic case without instability or extension limitation at minimum twoyear followup springerverlag | high union rate following surgical treatment of proximal fifth metatarsal stress fracture purpose the primary purpose of this study wa to determine the union rate and time for surgical and nonsurgical treatment of stress fracture of the proximal fifth metatarsal m the secondary purpose wa to ass the rate of adverse bone healing event delayed union nonunion and refractures a well a the return to sport time and rate method a literature search of the embase ovid medline pubmed cinahl web of science and google scholar database until march wa conducted methodological quality wa assessed by two independent reviewer using the methodological index for nonrandomized study minor criterion the primary outcome were the union time and rate secondary outcome included the delayed union rate nonunion rate refracture rate and return to sport time and rate a simplified pooling technique wa used to analyse the different outcome ie union rate time to union adverse bone healing rate return to sport rate and return to sport time per treatment modality additionally confidence interval were calculated for the union rate adverse bone healing rate and the return to sport rate result the literature search resulted in article of which thirteen study were included a total of fracture with a pooled mean followup of month were assessed overall the methodological quality of the included article wa low the pooled bone union rate wa ci and ci for surgically and nonsurgically treated fracture respectively the pooled radiological union time wa week for surgical treatment and week for nonsurgical treatment surgical treatment resulted in a delayed union rate of ci nonunion rate of ci and refracture rate of ci nonsurgical treatment resulted in a delayed union rate of ci a nonunion rate of ci and a refracture rate of ci respectively the return to sport rate at any level wa for both treatment modality return to preinjury level of sport time wa week fracture for surgical treatment and week fracture for nonsurgical treatment conclusion surgical treatment of stress fracture of the proximal fifth metatarsal result in a higher bone union rate and a shorter union time than nonsurgical treatment additionally surgical and nonsurgical treatment both showed a high return to sport rate at any level albeit with limited clinical evidence for nonsurgical treatment due to the underreporting of data level of evidence level iv systematic review the author | stress fracture of the medial malleolus in the professional soccer player demonstrate excellent outcome when treated with open reduction internal fixation and arthroscopic spur debridement purpose despite a debilitating effect on athletic performance and an incidence of up to of all stress fracture there have been only documented case of medial malleolus stress fracture mmsf to our knowledge in the literature the largest series to date is presented in this study of professional soccer player undergoing uniform operative treatment the author attempt to justify their preferred treatment of mmsfs in the professional soccer player with an emphasis on patient satisfaction clinical and radiographic union and return to high level sport the author aim to prove an association between lower limb varus alignment and the development of mmsfs method sixteen professional soccer player of mean age year were analysed a biomechanic assessment wa performed preoperative ctmri scan were performed to ass fracture line and the presence of anteromedial tibial andor talar spur which are the likely pathognomic lesion in the development of mmsfs all patient underwent open reduction and internal fixation with three screw a well a arthroscopic debridement of impringement spur and concentrated bone marrow aspirate into the fracture site patient completed the ogilvieharris score and all patient had ct scan at month and until union result all the patient in this cohort had causative bony spur that were debrided at surgery all of the cohort achieved clinical union all patient were able to return to professional football at the same level a prior to the injury there wa complete cohort follow up and of patient were graded a excellent and a good by the ogilvieharris score we noted of our cohort demonstrated varus malalignment either genu varum or hindfoot varus conclusion the author conclude that open reduction and internal fixation of mmsfs with screw combined with arthroscopic spur debridement result in excellent clinical outcome it can be concluded that varus lower limb malalignment is a risk factor for mmsfs given the treatment controversy for these injury the result herein demonstrate that aggressive multimodal operative treatment produce excellent outcome in high demand professional footballer this study is the first to report a biomechanic association which can alert the clinician to preventative measure such a hindfoot orthoses level of evidence iv european society of sport traumatology knee surgery arthroscopy esska |
| ACLR: Return to Sport | aclr, return, return sport, rts, sport, aclrsi, psychological, preinjury, reconstruction, selfefficacy | only one patient out of five achieves symmetrical knee function month after primary anterior cruciate ligament reconstruction purpose to ass the percentage of patient achieving symmetrical knee function month after primary anterior cruciate ligament acl reconstruction aclr and to identify factor affecting it achievement in a large cohort method data were extracted from our clinic database patient who underwent primary aclr from to and were assessed with the isokinetic quadriceps and hamstring muscle strength test and singleleghop test at the month followup were included in the study demographic data information on the graft used cartilage injury and concomitant meniscal surgery were reviewed patient who reached a limb symmetry index lsi of in all three test were considered to have achieved symmetrical knee function a multivariate logistic regression analysis wa used to determine whether patient age gender time from injury to surgery preinjury tegner activity level graft type cartilage injury and the presence of medial meniscus mm or lateral meniscus lm resection or repair were factor associated with the achievement of symmetrical knee function month after primary aclr result a total of patient male with a mean age of year were included data from all three test were available for patient the proportion of patient that achieved a lsi of wa and for isokinetic quadriceps muscle strength hamstring muscle strength and the singleleghop test respectively a total of patient achieved symmetrical knee function reaching a lsi of in all three test older age year or ci p mm resection or ci p and mm repair or ci p reduced the odds whereas the use of hamstring tendon ht autograft or ci p over bonepatellar tendonbone bptb autograft increased the odds of achieving symmetrical knee function conclusion only of the patient achieved symmetrical knee function month after primary aclr age year mm resection and mm repair reduced the chance whereas the use of ht autograft over bptb autograft increased the chance of achieving symmetrical knee function month after primary aclr this study show that most of the patient are yet to regain symmetrical knee function month after primary aclr and moreover it identifies several factor affecting it achievement in a large cohort the result of this study should be used to counsel patient about their expected functional recovery and to optimize rehabilitation and maximize knee function after aclr level of evidence iii the author | fear of reinjury following primary anterior cruciate ligament reconstruction a systematic review purpose this review aim to elucidate the most commonly reported method to quantify fear of reinjury or kinesiophobia and to identify key variable that influence the degree of kinesiophobia following primary anterior cruciate ligament reconstruction aclr method a systematic search across three database pubmed ovid medline and embase wa conducted from database inception to august h the author adhered to the prisma guideline and the cochrane handbook for systematic review of intervention quality assessment of the included study wa conducted according to the methodological index for nonrandomized study minor criterion result twentysix study satisfied the inclusion criterion and resulted in total patient with a mean age of year and a mean followup time of month postsurgery the mean minor score of the included study wa out of for noncomparative study and out of for comparative study eightyeight percent of included study used variation of the tampa scale of kinesiophobia tsk to quantify kinesiophobia and used anterior cruciate ligament return to sport after injury aclrsi the result of this study show a common association between higher kinesiophobia and poor patientreported functional status measured using international knee documentation committee ikdc score activity of daily living adl quality of life qol and sportsrecreation sr subscales of knee osteoarthritis and outcome score koos and lysholm score postoperative symptom and pain catastrophizing measured using the koos pain and symptom subscales and pain catastrophizing score pc also influenced the degree of kinesiophobia following aclr patient with an increased injury to surgery time and being closer to the date of surgery postoperatively demonstrated higher level of kinesiophobia le common variable included being a female patient low preoperative and postoperative activity status and low selfefficacy conclusion the most common method used to report kinesiophobia following primary aclr were variation of the tsk scale followed by aclrsi the most commonly reported factor influencing higher kinesiophobia in this patient population include lower patientreported functional status more severe postoperative symptom such a pain increased injury to surgery time and being closer to the date of surgery postoperatively kinesiophobia following primary aclr is a critical element affecting postsurgical outcome and screening should be implemented postoperatively to potentially treat in rehabilitation and recovery level of evidence iv the author under exclusive licence to european society of sport traumatology knee surgery arthroscopy esska | age gender quadriceps strength and hop test performance are the most important factor affecting the achievement of a patientacceptable symptom state after acl reconstruction purpose to ass the percentage of patient achieving an acceptable symptom state year after primary anterior cruciate ligament reconstruction aclr and to identify factor affecting it achievement in a large cohort method patient who underwent primary aclr at capio artro clinic stockholm sweden from to were identified in our clinic registry patient who had completed the knee injury and osteoarthritis outcome score koos at the year followup were included the primary outcome wa the achievement of a patientacceptable symptom state pas for each koos subscale a multivariate logistic regression analysis wa used to determine whether patient age gender time from injury to surgery preinjury tegner activity level graft type cartilage injury the presence of medial meniscus mm or lateral meniscus lm resection or repair and the recovery of month symmetrical limb symmetry index lsi of isokinetic quadriceps or hamstring strength and singleleghop test performance were factor associated with the achievement of a pas for each koos subscale result a total of primary aclrs were included more than of the patient reported a pas on four of the five koos subscales age year and an lsi of for month isokinetic quadriceps strength increased the odds of achieving a pas across all koos subscales female gender reduced the odds of achieving a pas on the pain or ci p activity of daily living adl or ci p and sport and recreation or ci p subscales the presence of an mm repair reduced the odds of achieving a pas on the pain or ci p subscale hamstring tendon ht autograft rather than bonepatellar tendonbone bptb autograft showed increased odds or ci p whereas a cartilage injury showed reduced odds or ci p of achieving a pas on the sport and recreation subscale an lsi of for month singleleghop test performance increased the odds of achieving a pas on the adl or ci p sport and recreation or ci p and quality of life or ci p subscales conclusion more than of the patient reported an acceptable symptom state on four of the five koos subscales year after primary aclr age year and female gender were the nonmodifiable factor that consistently increased and reduced respectively the odds of achieving a pas a symmetrical month isokinetic quadriceps strength and singleleghop test performance were the modifiable factor that consistently increased the opportunity of achieving a pas year after primary aclr level of evidence iii the author |
| TKA: Computer-assisted | navigation, conventional, computerassisted, tka, alignment, conventional tka, approach, ca, computer, navigated | doe computerassisted surgery improve postoperative leg alignment and implant positioning following total knee arthroplasty a metaanalysis of randomized controlled trial purpose computerassisted surgery ha been proposed a a technique to improve implant alignment during total knee arthroplasty tka however there is still a debate over the accuracy of placing the femoral and tibial component using computerassisted system in tka the aim of this study is to establish whether computerassisted surgery lead to superior mechanical leg axis and implant positioning than conventional technique in patient with primary tka method major electronic database were systematically searched to identify relevant study without language restriction a metaanalysis of randomized controlled trial rcts or quasircts wa performed in a random effect model a subgroup analysis wa conducted by type of navigation system to explore the clinical heterogeneity between these trial the following radiographic parameter were used to compare computerassisted surgery with conventional technique mechanical leg axis femoral component coronal alignment tibial component coronal alignment femoral component sagittal alignment and tibial component sagittal alignment result for the mechanical leg axis and coronal positioning of femoral and tibial component there are statistically significant reduction in the number of patient with malalignment in the ca group if the outlier cutoff value is or in the coronal and sagittal plane respectively subgroup analysis demonstrates that ctfree navigation system provide better alignment than conventional technique in the coronal and sagittal alignment of femoral component within and if the outlier cutoff value for the tibial sagittal alignment is the outlier percentage are higher in the ctfree navigation group than in the conventional group however there wa no significant difference in the tibial sagittal alignment at conclusion computerassisted surgery doe improve mechanical leg axis and component orientation in tkas however highquality rcts are necessary to determine whether surgeon could use computerassisted technique to achieve a targeted tibial slope in tka level of evidence therapeutic study systematic review of level iii study level ii springerverlag | the immediate effect of navigation on implant accuracy in primary miniinvasive unicompartmental knee arthroplasty the success of unicompartmental knee arthroplasty uka is highly dependent on the accuracy of the component alignment objective of the present study wa to evaluate the immediate effect of imagefree computer navigation technology on implant accuracy in primary miniinvasive uka this study review patient with primary isolated arthritis of the medial compartment of the knee that underwent unicompartmental knee arthroplasty through a minimally invasive approach a cohort of the most recent consecutive ukas implanted with standard instrumentation wa followed by a cohort of the very first consecutive case after conversion to the navigated technique there wa no variability regarding implant oxford meniscal unicompartmental knee systembiomet orthopedics inc warsaw indiana usa surgeon and surgical technique except for the use of the navigation system treon plusmedtronic inc minnesota mi usa the axis alignment and accuracy of implant positioning wa measured on postoperative longleg standing radiograph and standard lateral xrays with regard to the valgus angle and the coronal and sagittal component angle in addition preoperative deformity of the mechanical leg axis tourniquet time age gender and body mass index were correlated statistical analysis were performed using the spss spss inc chicago il usa software package optimal implant alignment including all measurement in the desired angular range wa significantly p higher in the navigated cohort navigation eliminated outlier in the frontal mechanical alignment and coronal orientation of the femoral component totally and significantly p furthermore navigation narrowed the range of outlier in all other plane of component orientation there were no statistically significant difference in the mean numerical value between the cohort except for the frontal mechanical alignment p and coronal tibial alignment p the average tourniquet time wa increased by min in the navigated cohort our result indicate that navigation immediately improves accuracy of bone cut and reduces the number of outlier with implementation in uka springerverlag | improved accuracy of component alignment with the implementation of imagefree navigation in total knee arthroplasty accuracy of implant positioning and reconstruction of the mechanical leg axis are major requirement for achieving good longterm result in total knee arthroplasty tka the purpose of the present study wa to determine whether imagefree computer navigation technology ha the potential to improve the accuracy of component alignment in tka cohort of experienced surgeon immediately and constantly one hundred patient with primary arthritis of the knee underwent the unilateral total knee arthroplasty the cohort of tkas implanted with conventional instrumentation wa directly followed by the cohort of the very first computerassisted tkas all surgery were performed by two senior surgeon all patient received the zimmer nexgen total knee prosthesis zimmer inc warsaw in usa there wa no variability regarding surgeon or surgical technique except for the use of the navigation system stealthstation treon plus medtronic inc minnesota mi usa accuracy of implant positioning wa measured on postoperative longleg standing radiograph and standard lateral xrays with regard to the valgus angle and the coronal and sagittal component angle in addition preoperative deformity of the mechanical leg axis tourniquet time age and gender were correlated statistical analysis were performed using the spss spss inc chicago il usa software package independent ttests were used with significance set at p twotailed to compare difference in mean angular value and frontal mechanical alignment between the two cohort to compute the rate of optimally implanted prosthesis between the two group we used the k test the average postoperative radiological frontal mechanical alignment wa of varus range of valgu of varus sd in the conventional cohort and of varus range of varus sd in the navigated cohort including all criterion for optimal implant alignment case in the conventional cohort and case in the navigated cohort have been implanted optimally the average difference in tourniquet time wa modest with additional min in the navigated cohort compared to the conventional cohort our finding suggest that the experienced knee surgeon can improve immediately and constantly the accuracy of component orientation using an imagefree computerassisted navigation system in tka the computerassisted technology ha shown to be easy to use safe and efficient in routine knee replacement surgery we believe that navigation is a key technology for various current and future surgical alignment topic and minimalinvasive lower limb surgery springerverlag |
| Tendon-bone Healing | patellar tendon, tendon, proximal hamstring, hamstring, patellar, rupture, periosteum, healing, tendon rupture, tendonbone | enhancement of rotator cuff tendonbone healing with injectable periosteum progenitor cellsbm hydrogel in vivo purpose the fixation and incorporation of ruptured rotator cuff tendon to bone is a major concern in rotator cuff repair surgery rotator cuff repair usually fails at the tendonbone interface especially in case of large or massive tear to enhance tendonbone healing an injectable hydrogel made with periosteal progenitor cellsppcs and poly ethylene glycol diacrylate pegda tethered with bone morphogenic proteinbm wa developed to encourage extracellular matrix synthesis for tendontobone healing in rotator cuff repair method the infraspinatus tendon wa cut from the greater tuberosity and repaired through a transosseous tunnel with the injectable progenitor cellbm hydrogel applied between the tendonbone interface the injectable hydrogel wa prepared from poly ethylene glycol diacrylate pegda containing of the photoinitiator bm tethered with polyethylene glycol peg wa blended to the hydrogel rabbit periosteal progenitor cell ppcs isolated from periosteum were mixed with hydrogel and injected on the tendonbone interface ultraviolet radiation nm wa applied for s to photopolymerize the injection and solidify the hydrogel the rabbit were killed at and week the morphological characteristic of the healing tendontobone interface were evaluated by histological and immunohistochemical method the biomechanical test wa done to determine healing attachment strength result at both the and week killing histological analysis of the tendonbone interface showed an increasing fibrocartilage and bone layer formed in the tendonbone interface in pegda group at week fibrocartilagelike tissue wa observed in a focal area at week further matrix deposition occurred with fibrocartilage formation in the tendonbone junction and bone formation appeared near host bone immunohistochemistry revealed the presence of aggrecan and type ii collagen biomechanical testing revealed a higher maximum pullout load at all time point with a statistically significant difference at and week postoperatively conclusion pegda hydrogel wa approved a an adequate matrix for the encapsulation of cell and signal factor and a an effective local delivery method to the tendonbone interface through injection and photopolymerization the ppcsbmhydrogel provides a powerful inductive ability between the tendon and the bone and enhances tendonbone healing through the neoformation of fibrocartilage springerverlag | rotator cuff repair with periosteum for enhancing tendonbone healing a biomechanical and histological study in rabbit during rotator cuff repair surgery fixation and incorporation of ruptured rotator cuff tendon into the bone is a major concern the repair usually fails at the tendonbone interface especially in case where the tear is massive the periosteum contains multipotent stem cell that have the potential to differentiate into osteogenic and chondrogenic tissue which may restore the original structure at the tendonbone interface fibrocartilage in this study we investigated the effect of periosteum on the healing of the infraspinatus tendon and bone using a clinically relevant rabbit model of rotator cuff tear we used skeletally mature new zealand white rabbit in the study the infraspinatus tendon at right limb wa detached from greater tuberosity and a periosteal flap taken from the proximal tibia wa sutured onto the torn end of tendon the contralateral limb which wa used a a control received the same treatment without a periosteum the rabbit were sacrificed at and week and the tendonbone interface wa put to histological exam and the biomechanical testing to ass strength of tendonbone interface histological analysis of the tendonbone interface revealed that the periosteum formed a fibrous layer over the interface between tendon and bone at week fibrotic tissue showed progressive integration over the interface between cuff tendon and bone at week progressive formation of fibrovascular tissue and fibrocartilage wa observed between tendon and bone at week extensive formation of fibrocartilage and bone wa noted in the interface the significant increase of failure load with time indicated a progressive increase in the tendonbone incorporation strength at week after operation the attachment strength of the limb with the periosteum treated wa higher than that of the control limb however this difference wa not statistically significant at and week a statistically significant increase wa noted in the attachment strength of the limb treated with the periosteum most specimen failed at the tendonbone interface in the treatment of a torn rotator cuff in rabbit model improved healing process with greater attachment strength could be achieved by suturing the periosteum between the end of tendon and the bone trough histological examination revealed that the cambium layer of the periosteum could serve a a potent interface layer and become progressively mature and organized during the healing process resulting in fibrocartilage formation and the subsequent integration of the disrupted tendon into the bone biomechanical testing revealed a progressive increase in the attachment strength with time indicating the progressive tendonbone incorporation when performing rotator cuff repair in a large or massive tear a periosteal flap can be sutured onto the torn end of tendon to enhance tendonbone healing springerverlag | the effect of bone marrow or periosteum on tendontobone tunnel healing in a rabbit model the purpose of this study wa to investigate whether a grafting technique using either periosteum or bone marrow a an adjunct would reconstitute more favorable tendon anchorage morphology with improved tensile strength in a bone tunnel model we hypothesized that autogenous bone marrow aspirate can enhance the tendonbone attachment a well a a freshly harvested periosteum because both tissue contain pluripotent cell thirtysix skeletally mature new zealand white rabbit were utilized for the tendon graft healing in a bone tunnel model the extensor digitorum longus tendon wa detached from it femoral insertion and transplanted through a bone tunnel into the proximal tibia three group were compared for the group p periosteum a periosteumwrapped tendon wa fixed into the tunnel through the proximal tibial metaphysis for the group bm bone marrow instead of periosteum augmentation fresh bone marrow wa injected into the tendon graft that would sit inside the tunnel for the group c control the limb underwent a similar operation with neither the periosteum enveloping nor bone marrow injecting the tendon at and week after surgery two rabbit were used for light and electron microscopic examination and ten rabbit were used for biomechanical test in each group the interface tissue between bone and tendon wa thicker and le organized in group c compared to group p and bm at week ultrastructurally the interface tissue wa loosely organized in group c compared to others bone ingrowth into tendon wa more obvious in group p and bm compared to group c the proliferation of cartilage island wa observed within bone tunnel of both group p and bm but a welldefined fibrocartilage zone wa noted only in group bm at the interface at week biomechanical finding at week the average failure load of group p wa significantly higher than the others p at same time point in term of stiffness while group p wa significantly higher than the other group p group bm wa also significantly higher than that of group c p at week in term of failure load there wa a statistical significant difference only between group bm and c p at the same time point stiffness value were not statistically different among the three group based on the histological and biomechanical finding the present study demonstrated that periosteum had a positive effect when compared to bone marrow and control group on the tendontobone healing at an early time point week and bone marrow wa also effective at week time point compared to the control group in an extraarticular bone tunnel in rabbit the presence of pluripotent cell in both the bone marrow and the periosteum may be the possible mechanism for enhanced healing periosteum had a positive effect at an early time point week bone marrow wa more effective at week therefore it is possible that a combination of wrapping periosteum and injecting bone marrow to the tendon graft would have a synergistic effect early and strong to prove this hypothesis future study which would combine both method are needed springerverlag |
| TKA: Blood Loss | blood, blood loss, tourniquet, transfusion, loss, blood transfusion, txa, tranexamic, tranexamic acid, haemoglobin | the effect of tranexamic acid on blood loss and use of blood product in total knee arthroplasty a metaanalysis purpose study have shown that tranexamic acid txa reduces blood loss and transfusion need in patient undergoing total knee arthroplasty tka however no single study ha been large enough to definitively determine whether the drug is safe and effective we report a systematic review and metaanalysis of randomised controlled trial evaluating the efficacy and safety of txa in reducing blood loss and transfusion in tka method a comprehensive literature search wa done in cochrane library medline embase and cnki two reviewer independently identified the eligible study assessed their methodological quality and extracted data the data were evaluated using the generic evaluation tool designed by the cochrane bone joint and muscle trauma group the relevant data were analyzed using revman result fifteen randomized controlled trial involving patient were included the use of txa reduced total blood loss by a mean of ml confidence interval ci to intraoperative blood loss by a mean of ml ci and postoperative blood loss by a mean of ml ci to txa led to a significant reduction in the proportion of patient requiring blood transfusion risk difference there were no significant difference in deepvein thrombosis dvt pulmonary embolism or other complication among the study group conclusion metaanalysis indicates that txa may reduce postoperative total blood loss and transfusion in patient undergoing tka txa led to a significant reduction in the proportion of patient requiring blood transfusion level of evidence therapeutic study systematic review of level i study with inconsistent result level ii springerverlag | intravenous versus topical tranexamic acid administration in primary total knee arthroplasty a metaanalysis purpose this metaanalysis wa designed to compare the effectiveness and safety of intravenous iv versus topical administration of tranexamic acid txa in patient undergoing primary total knee arthroplasty tka by evaluating the need for allogenic blood transfusion incidence of postoperative complication volume of postoperative blood loss and change in haemoglobin level method study were included in this metaanalysis to check whether they assessed the allogenic blood transfusion rate postoperative complication including pulmonary thromboembolism pte or deep vein thrombosis dvt volume of postoperative blood loss via drainage estimated blood loss total blood loss and change in haemoglobin level before and after surgery in primary tka with txa administered through both the iv and topical route result ten study were included in this metaanalysis the proportion of patient requiring allogenic blood transfusion or ci n and the proportion of patient who developed postoperative complication including pte or dvt or ci to n did not significantly differ between the two group there wa ml le blood loss via drainage ci to ml n ml greater estimated blood loss ci to ml n and ml greater total blood loss ci to ml n in the topical txa group a compared to the iv txa group the two group were also similar in term of the change in haemoglobin level gdl ci to gdl n conclusion in primary tka there are no significant difference in the transfusion requirement postoperative complication blood loss and change in haemoglobin level between the iv and topical administration of txa in addition result from subgroup analysis evaluating the effect of the time of txa administration through the iv route suggested that double iv dose of txa is more effective than single dose in term of the transfusion requirement and blood loss via drainage the current metaanalysis indicates that iv administration of mgkg of txa min before inflation of the tourniquet followed by mgkg of txa min before deflation of the tourniquet is effective and safe the topical administration of g of txa mixed with ml of normal saline after wound closure could be an alternative option in patient at greater risk of thromboembolic complication level of evidence metaanalysis level iii european society of sport traumatology knee surgery arthroscopy esska | the effect of tourniquet use in total knee arthroplasty a randomized controlled trial purpose tourniquet are still widely used in total knee arthroplasty tka although they may be associated with several adverse effect an observerblinded randomized controlled trial wa performed to evaluate the effect of tourniquet use in tka method fifty participant who underwent staged bilateral tka were recruited for this study the firstside tka wa randomly allocated to either longduration tourniquet use or shortduration tourniquet use followed by a onth washout period and crossover to the other tourniquet strategy for the oppositeside tka blood loss wa monitored perioperatively the operating time allogeneic blood transfusion rate thigh pain knee pain limb swelling clinical outcome a measured by the likerttype western ontario and mcmaster university womac score straight leg raising and knee active range of motion rom were also recorded result the longduration tourniquet group exhibited reduced total blood loss ml confidence interval ci to p and intraoperative blood loss ml ci to p compared with the shortduration tourniquet group however there were greater postoperative blood loss ml ci to p and hidden blood loss ml ci to p in the longduration tourniquet group the shortduration tourniquet group showed better outcome for thigh and knee pain limb swelling womac score at week followup straight leg raising and knee rom similar allogeneic blood transfusion rate were observed for both group conclusion total and intraoperative blood loss were reduced with the longduration tourniquet use whereas the shortduration tourniquet use would reduce postoperative and hidden blood loss without increasing the allogeneic blood transfusion rate in addition shortduration tourniquet use would result in faster recovery and le pain during the early rehabilitation period following tka level of evidence i european society of sport traumatology knee surgery arthroscopy esska |
| PJI | infection, septic, septic arthritis, pji, twostage, antibiotic, arthritis, joint infection, vancomycin, periprosthetic | alphadefensin a a diagnostic tool in revision total knee arthroplasty with unexpected positive intraoperative culture and unexpected culture negative intraoperative culture purpose unexpectedpositiveintraoperativecultures upic are common in presumed aseptic revisiontotalkneearthroplasties rtka however the clinical significance is not entirely clear in contrast in some presumably septic rtka identification of an underlying pathogen wa not possible socalled unexpectednegativeintraoperativecultures unic the purpose of this study wa to evaluate the potential use of synovial alphadefensin ad level in these patient method synovial ad level from rtkas were evaluated retrospectively from our prospectively maintained institutional periprostetic joint infection pji biobank and database the international consensus meeting icm criterion wa used to define the study group sample from upics with a minimum of one positive intraoperative culture icm n and unics icm n were compared to septic culturepositive sample icm and aseptic culturenegative icm moreover adlateralflowassay adlf and an enzymelinkedimmunosorbentassay elisa in detecting the presence of ad in native and centrifuged synovial fluid specimen wa performed concentration of ad determined by elisa and adlf method a well a microbiological and histopathological result serum and synovial parameter along with demographic factor were analysed result ad wa positive in sample from the septic culturepositive group and in sample in the unic group all upic sample showed a negative ad result positive ad sample were highly associated with culture positive and histopathological result p no highvirulent microorganism were present in the upic group compared to infectedgroup high virulent microorganism showed a positive ad result in of the case methicillin resistant staphylococcus epidermis mrse infection had significantly higher ad level than with methicillin susceptible s epidermdis msse p elisa and adlf test were positive with centrifuged and native synovial fluid conclusion ad showed a solid diagnostic performance in infected and noninfected revision and it provided an additional value in the diagnosis of upic and unic associated to rtkas pathogen virulence a well a antibiotic resistance pattern may have an effect on ad level centrifugation of synovial fluid had no influence on adlf result the author under exclusive licence to european society of sport traumatology knee surgery arthroscopy esska | recommendation for periprosthetic joint infection pji prevention the european knee associate ekainternational committee american association of hip and knee surgeon aahksarthroplasty society in asia asia survey of member purpose periprosthetic joint infection pjis represent a devastating consequence of total joint arthroplasty the european knee associate eka the american association of hip and knee surgeon aahks international committee and the arthroplasty society in asia asia board member were interested in quantifying difference in arthroplasty surgeon use of various pji prevention measure to provide clinical recommendation to reduce pji incidence method a prospective microsoft form online survey wa distributed among eka aahks international committee and asia member and their affiliated arthroplasty surgeon the survey consisted of single and multiple response question focused on pji prevention strategy at three perioperative period preoperatively intraoperatively and postoperatively result three hundred and ninetyfour arthroplasty surgeon from different continent completed the survey preoperative a pji risk stratification routinely set threshold eg bmi hgbc to be met to qualify for surgery only review past medical history use machine learning to personalize pji risk b bmi limit no limit bmi bmi bmi c nutritional status do not screen among those who screen their patient albumin is the single most used marker d hyperglycemiadiabetes check this comorbidity use hgbc a single best screening test e mrsa nasal colonization do not test test all patient test selectively intraoperative a antibiotic prophylaxis in highrisk patient use single antibiotic for h use double antibiotic for h use singledouble antibiotic for day postoperatively b skincleansing at home chlorhexidine sponge clipper c intraoperative skin disinfection single chlorhexidine double chlorhexidinepovidoneiodin single povidoneiodine d tranexamic acid txa to reduce bleedingssi yes double iv dose single iv dose intraarticular injection e surgical suction drain do not use drain use a drain h f intraarticular lavage use only saline use dilute povidoneiodine g antibiotic local delivery to prevent pji use antibioticadded cement postoperative a routine monitoring of pji serologic marker only in symptomatic patient do not in all patient b serologic marker to rule inout pji crp sedrate wbc c synovial fluid test to rule inout pji culturesensitivity wbc count crp conclusion this survey demonstrated that notable difference still exist in the application of pji preventive measure across different geographic area optimizing the patient preoperatively and applying multimodal intraoperative strategy represent newer clinically relevant step in the effort to reduce the burden of pji more uniform guideline still need to be produced from international scientific society in order facilitate a more comprehensive approach to this devastating complication level of evidence iv graphical abstract figure not available see fulltext this is a u government work and not under copyright protection in the u foreign copyright protection may apply | microorganism responsible for periprosthetic knee infection in england and wale purpose this study aimed to delineate epidemiology of infecting microorganism genus in firsttime revision knee arthroplasty for indication of periprosthetic joint infection in england and wale using linked registry data method from the national joint registry database for england and wale a consecutive series of primary knee arthroplasty performed between april and january that went on to have a revision for periprosthetic infection were identified n each case wa then linked to microbiology data held by public health england in order to identify infecting microorganism at time of revision surgery established from intraoperative culture following data linkage culture result at time of revision surgery were identified in a group of patient the demographic characteristic of five microorganism group were compared pure staphylococcus single genus pure streptococcus single genus other grampositive infection single genus gramnegative infection single genus and mixed genus infection result staphylococcus specie wa the most common organism genus isolated after revision of a primary implant for infection and present in of case overall of patient with a singlegenus infection and of patient with mixed genus infection a pure staphylococcal infection wa present in of all case a singlegenus infection wa responsible for infection in of case and mixed genus were responsible in of case a significant difference wa observed for mean age at primary procedure in the cohort of patient where there wa an isolated pure streptococcal infection year when compared to gramnegative infection year no other significant difference were observed between microorganism group in term of bmi gender asa grade indication for primary procedure and primary implant characteristic conclusion staphylococci were the most commonly isolated organism specie responsible for periprosthetic infection of primary arthroplasty in england and wale this information can be used by surgeon to benchmark and audit their own practice against national publicly available data furthermore this study ha shown that even when using the largest national database available there is a substantial volume of missing data antimicrobial resistance represents a growing clinical problem with significant health and social cost in order to counteract this threat this study would advocate the consolidation of national microbial data in order to guide effective strategy towards targeting and combating the threat of antimicrobial resistance level of evidence iv european society of sport traumatology knee surgery arthroscopy esska |
| Knee Phenotyping | varus, alignment, deformity, valgus, phenotype, hka, varus knee, varus deformity, angle, coronal | phenotyping of hipkneeankle angle in young nonosteoarthritic knee provides better understanding of native alignment variability purpose there is a lack of knowledge about the native coronal knee alignment in d the currently used classification system neutral valgus and varus oversimplifies the coronal knee alignment the purpose of this study wa therefore to investigate the coronal knee alignment in nonosteoarthritic knee using dreconstructed ct image and to introduce a classification system for the overall knee alignment based on phenotype method the hospital registry wa searched for patient younger than year and older than who received a ct according to the imperial knee protocol patient with prosthesis osteoarthritis fracture or injury of the collateral ligament were excluded finally nonosteoarthritic knee of patient remained male and female mean age standard deviation sd year the overall lower limb alignment wa defined a the hipkneeankle angle hka which is formed by line connecting the center of the femoral head the knee and the talus the angle wa measured using a commercially planning software kneeplan d symbios yverdon le bains switzerland descriptive statistic such a mean range and measure of variance eg standard deviation are presented based on these result the currently used classification system wa evaluated and a new system based on phenotype wa introduced these phenotype consist of a phenotypespecific mean value a hka value and cover a range of from this mean eg the mean value represent increment of the angle starting from the overall mean value mean hka increment and and the distribution of these limb phenotype wa assessed result the overall mean hka wa varus and value ranged from varus to valgus the mean hka value for male and female were and respectively which implied a significant gender difference r the most common limb phenotype in male wa neu hka followed by var hka and val hka the most common limb phenotype in female wa neu hka followed by val hka and var hka conclusion the measurement using dreconstructed ct image confirmed the great variability of the overall lower limb alignment in nonosteoarthritic knee however the currently used classification system neutral varus valgus oversimplifies the coronal alignment and therefore the introduced classification system based on limb phenotype should be used this will help to better understand individual coronal knee alignment level of evidence level iii retrospective cohort study european society of sport traumatology knee surgery arthroscopy esska | phenotyping the knee in young nonosteoarthritic knee show a wide distribution of femoral and tibial coronal alignment purpose there is a lack of knowledge about the joint line orientation of the femur and tibia in nonosteoarthritic knee the primary purpose of the present study wa to evaluate the orientation of the joint line in native nonosteoarthritic knee using dreconstructed ct scan the secondary purpose wa to identify knee phenotype to combine the information of the femoral and tibial alignment method a total of nonosteoarthritic knee of patient male to female ratio mean age standard deviation year year were retrospectively included from our registry all patient received ct of the knee according to the imperial knee protocol the orientation of the femoral and tibial joint line wa measured in relation to their mechanical axis femoral mechanical angle fma and tibial mechanical angle tma using a commercially planning software kneeplan d symbios yverdon le bains switzerland the value of fma and tma were compared between male and female descriptive statistic such a mean range and measure of variance eg standard deviation were presented based on these result phenotype were introduced for the femur and tibia these phenotype based on fma and tma value consist of a mean value and cover a range of from this mean increment the distribution of femoral and tibial phenotype and their combination knee phenotype were calculated for the total group and for both gender result the overall mean fma standard deviation sd wa and value ranged from varus to valgus the overall mean tma sd wa with a range of varus to valgus fma and tma showed significant gender difference p female showed more valgus alignment than male the most common femoral phenotype wa neutral in both gender the most common tibial phenotype wa neutral in the male knee and valgus in the female knee in male the most frequent combination knee phenotype wa a neutral phenotype in the femur and a neutral phenotype in the tibia in female it wa a neutral femoral phenotype and a valgus tibial phenotype conclusion dreconstructed ct scan confirmed the great variability of the joint line orientation in nonosteoarthritic knee the introduced femoral and tibial phenotype enable the evaluation of the femoral and tibial alignment together knee phenotype the variability of knee phenotype found in this young nonosteoarthritic population clearly show the need for a more individualized approach in tka level of evidence iii european society of sport traumatology knee surgery arthroscopy esska | seven phenotype of varus osteoarthritic knee can be identified in the coronal plane purpose recommendation for resecting distal femur and proximal tibia in mechanical and anatomical alignment technique are standardized kinematic alignment propagates individualizing resection plane whether significant variation exists to warrant departure from standardized resection plane ha not been shown thus far in a large cohort of knee and with a wide range of varus deformity the null hypothesis of this study wa that there wa no phenotypic variation in varus osteoarthritic knee the aim of this paper wa to determine whether distinct phenotype could be identified based on variation in coronal femoral and tibial morphology which could aid in surgical planning and categorizing varus knee for future study method fullleg weightbearing radiograph were analyzed preoperative of contralateral arthritic knee measurement made were of hka hipkneeankle angle vca valgus correction angle mldfa lateral mechanical distal femoral angle aldfa lateral anatomical distal femoral angle mpta medial proximal tibial angle mnsa medial neck shaft angle tama angle between tibial mechanical and anatomical ax and tpdr percentage length of tibia proximal to extraarticular deformity result seven distinct type were identified covering knee reducible to broad phenotype were type neutral knee showing value close to reported normal knee mean vca mldfa aldfa were type intraarticular varus with medial intraarticular bone loss mean mldfa mpta vca of were type extraarticular varus with extraarticular deformity ead type had proximal tibial ead type b had tibial diaphyseal ead type c had femoral ead mean vca hka and severe medial bone loss mean mldfa mpta were type valgoid type with feature of valgus knee type a had medial femoral bowing mean vca type b had significant distal femoral valgus mean mldfa aldfa conclusion the null hypothesis that there wa no phenotypic variation in varus osteoarthritic knee wa rejected a considerable variation wa found in coronal morphology of femur and tibia four broad phenotypic group could be identified plane of the knee joint articular surface wa quite variable this ha relevance to planning and performance of corrective osteotomy unicompartmental and total knee arthroplasty level of evidence iii retrospective cohort study european society of sport traumatology knee surgery arthroscopy esska |
| Intra-articular Biologic Injections | injection, prp, cell, msc, intraarticular, plasma, stem cell, intraarticular injection, plateletrich plasma, plateletrich | bone marrow aspirate concentrate injection provide similar result versus viscosupplementation up to month of followup in patient with symptomatic knee osteoarthritis a randomized controlled trial purpose the purpose of this doubleblind randomized controlled trial rct wa to compare clinical improvement and radiographic finding up to year of followup of a single intraarticular injection of bone marrow aspirate concentrate bmac versus hyaluronic acid ha for the treatment of knee osteoarthritis oa the hypothesis wa that bmac injection could lead to better clinical and radiographic result compared to viscosupplementation method patient with bilateral knee oa were randomized to one intraarticular injection of tibialderived bmac in one knee and one ha injection in the contralateral knee sixty patient were enrolled and were studied up to the final followup men woman mean age year for a total of knee patient were evaluated before the injection and at and month with the ikdc subjective score va for pain and the koos score minimal clinically important difference mcid patient treatment judgement and adverse event were documented a well a bilateral xrays rosenberg view before and after treatment result no severe adverse event nor difference were reported in term of mild adverse event v p n and treatment failure v p n in bmac and ha group respectively the ikdc subjective score improved from baseline to all followup for bmac p while it improved up to month p and then decreased at month p for ha compared to ha bmac showed a higher improvement for va pain at v p and month v p the analysis based on oa severity confirmed this difference only in kellgrenlawrence knee while comparable result were observed in moderatesevere oa radiographic evaluation did not show knee oa deterioration for both treatment group without intergroup difference conclusion bmac did not demonstrate a clinically significant superiority at shortterm compared to viscosupplementation reporting overall comparable result in term of clinical score failure adverse event radiographic evaluation mcid achievement and patient treatment judgment however while ha result decreased over time bmac presented more durable result in mild oa knee level of evidence level i the author under exclusive licence to european society of sport traumatology knee surgery arthroscopy esska | intraarticular injection of expanded mesenchymal stem cell with and without addition of plateletrich plasma are safe and effective for knee osteoarthritis purpose to compare the effectiveness and safety of intraarticular injection of autologous expanded mesenchymal stromal stem cell alone msc or in combination with plateletrich plasma msc prp in patient with knee osteoarthritis method eighteen patient year with radiographic symptomatic knee osteoarthritis dejour grade iiiv were randomized to receive intraarticular injection of msc n or msc prp n injection were performed week after bone marrow aspiration ml which wa obtained from both posterior iliac crest result the knee injury and osteoarthritis outcome score koos improved significantly throughout the month for both group p no statistically significant difference between group were found in koos subscales and global score improvement at month endpoint n the msc group showed significant improvement in the pain function and daily living activity and sport and recreational activity subscales p similarly the msc prp group showed significant improvement in the pain function and daily living activity and quality of life subscales p the average number of fibroblast colony forming unit cfuf wa for msc group and for msc prp group minimal adverse effect were seen in both group adverse event in patient conclusion intraarticular injection of expanded msc alone or in combination with prp are safe and have a beneficial effect on symptom in patient with symptomatic knee osteoarthritis adding prp to the msc injection did not provide additional benefit these result are encouraging and support the recommendation of this minimally invasive procedure in patient with knee osteoarthritis without requiring hospitalization the cfuf result may be used a reference for future research level of evidence prospective cohort study level ii european society of sport traumatology knee surgery arthroscopy esska | intraarticular injection of plateletrich plasma decrease pain and improve functional outcome than sham saline in patient with knee osteoarthritis purpose to compare the longterm clinical efficacy provided by intraarticular injection of either pure plateletrich plasma pprp or sham saline to treat knee osteoarthritis koa method this prospective parallelgroup doubleblind multicenter shamcontrolled randomized clinical trial recruited participant with koa from orthopedic department at nine public hospital five tertiary medical center four secondary medical unit starting january with followup completed on february participant were randomly allocated to intervention in a ratio data were analyzed from march to july three session every week of pprp or sham saline injected by physician the primary outcome wa the western ontario and mcmaster university arthritis index womac at month of followup secondary outcome included the international knee documentation committee ikdc subjective score visual analogue scale va score intraarticular biochemical marker concentration cartilage volume and adverse event laboratory of each hospital analyzed the content and quality of pprp result participant woman with koa who received three session of pprp n mean age year or sham saline n mean age year injection completed the trial the mean platelet concentration in prp is fold confidence interval greater than that of whole blood both group showed significant improvement in ikdc womac and va score at month of followup however only the pprp group showed a sustained improvement in clinical outcome measurement at month p there were statistically significant difference between the pprp and sham saline group in all clinical outcome measurement at each followup time point p the benefit of pprp wa clinically better in term of womacpain womacphysical function and womactotal at and month of followup no clinically significant difference between treatment were documented in term of womacstiffness at any followup a clinically significant difference favoring pprp group against saline in term of ikdc and va score wa documented at and month of followup at month after injection tnfa and ib level in synovial fluid were lower in the pprp group p tibiofemoral cartilage volume decreased by a mean value of m in the pprp group and m in the saline group over month and the difference between the group wa statistically significant intergroup difference m ci to m p conclusion in this randomized clinical trial of patient with koa pprp wa superior to sham saline in treating koa pprp wa effective for achieving at least month of symptom relief and slowing the progress of koa with both pprp and saline being comparable in safety profile the author under exclusive licence to european society of sport traumatology knee surgery arthroscopy esska |
| Synovial Tumors & Intra-articular Masses | cyst, ganglion, fat pad, fat, pad, synovial, lipoma, pigmented villonodular, villonodular, pigmented | pressure change in the kager fat pad at the extreme of ankle motion suggest a potential role in achilles tendinopathy introduction the kager fat pad is one of the largest soft tissue structure local to the ankle joint yet it is poorly understood it ha been hypothesised to have a role in achilles tendinopathy this study aimed to investigate the pressure area in the kager fat pad adjacent to the achilles tendon and to ass the anatomy and deformation of the kager fat pad in cadaver method twelve fresh frozen cadaveric ankle mean age year range were mounted in a customized testing rig enabling plantar flexion and dorsiflexion of the ankle with the achilles tendon loaded a needle tipped pressure sensor wa inserted in two area of the kager fat pad under ultrasound guidance retrocalcaneal bursa and at cm proximal from achilles insertion pressure reading were recorded at different flexion angle following testing the specimen were dissected to expose the kager fat pad and retrieve it for analysis mri image were also taken from three healthy volunteer and the kager fat pad deformation examined result mean pressure significantly increased in all specimen at terminal ankle plantar and dorsi flexion in both region p the kager fat pad wa consistently adherent to the achilles at it posterior aspect for a mean length of cm sd of kfp length the most distal part of the kager fat pad wa the exception and it detached from the achilles to give way to the retroalcaneal bursa for a mean length of cm sd of kfp length the bursal space is partially occupied by a constant wedge extension of kager fat pad the mean volume of the whole kager fat pad wa ml sd video and mri demonstrated that the kager fat pad undergoes significant deformation during plantar flexion a it is displaced superiorly by the achilles with the wedge being forced into the retrocalcaneal bursal space conclusion the kager fat pad doe not remain static during ankle range of motion but deforms and it pressure also change this observation support the theory that it act a a shockabsorber to the achilles tendon and pathological change to the fat pad may be clinically important in the development of achilles tendinopathy european society of sport traumatology knee surgery arthroscopy esska | distinct extraarticular invasion pattern of diffuse pigmented villonodular synovitistenosynovial giant cell tumor in the knee joint purpose pigmented villonodular synovitis pvnstenosynovial giant cell tumor tgct is a benign proliferative lesion of the synovium the bursa and the tendon sheath little is known about the anatomical distribution pattern of diffuse extraarticular pvnstgct around the knee joint in this retrospective study anatomical distribution of pvnstgct using magnetic resonance imaging mri and arthroscopy wa analyzed method this study wa designed a a retrospective observational crosssectional study based on mri and arthroscopy twentyfour pvnstgct patient knee who underwent arthroscopic or posterior open surgery between and were enrolled of these eight intraarticular and diffuse extraarticular pvnstgct of the knee were classified the anatomical location of the pvnstgct mass were determined with a newly devised mapping scheme analysis wa performed on the prevalence of each compartment and agreement rate between each compartment result the point prevalence of intraarticular posterior compartment wa higher in diffuse extraarticular pvnstgct group compared with intraarticular pvnstgct group the point prevalence of diffuse pvnstgct wa most prevalent in the extraarticular posterolateral compartment out of diffuse extraarticular pvnstgct patient and second most common in the below to joint capsule compartment out of the agreement rate wa the highest between intraarticular posterolateral and extraarticular posterolateral compartment conclusion extraarticular invasion of diffuse pvnstgct occurred in specific pattern in the knee joint extraarticular lesion were always accompanied by lesion in intraarticular compartment in particular lesion in the intraarticular posterior compartment were observed in all of the diffuse extraarticular pvnstgct patient the point prevalence of diffuse extraarticular pvnstgct for each compartment wa the highest out of in extraarticular posterolateral compartment in contrast invasion to the extraarticular posteromedial side wa le frequent out of than to the extraarticular posterolateral side knowing where the lesion frequently occur may provide important information for deciding the timing method and extent of surgery level of evidence level iv european society of sport traumatology knee surgery arthroscopy esska | the infrapatellar fat pad is a dynamic and mobile structure which deforms during knee motion and ha proximal extension which wrap around the patella purpose the infrapatellar fat pad ifp is a common cause of knee pain and loss of knee flexion and extension however it anatomy and behavior are not consistently defined method thirtysix unpaired fresh frozen knee median age year range were dissected and ifp attachment and volume measured the rectus femoris wa elevated suprapatellar pouch opened and video recorded looking inferiorly along the femoral shaft at the ifp a the knee wa flexed the patellar retinacula were incised and the patella reflected distally the attachment of the ligamentum mucosum lmuc to the intercondylar notch wa released from the anterior cruciate ligament acl both meniscus and to the tibia via meniscotibial ligament ifp strand projecting along both side of the patella were elevated and the ifp dissected from the inferior patellar pole magnetic resonance imaging mri of one knee at ten flexion angle wa performed and the ifp patella tibia and femur segmented result in all specimen the ifp attached to the inferior patellar pole femoral intercondylar notch via the lmuc proximal patellar tendon intermeniscal ligament both meniscus and the anterior tibia via the meniscotibial ligament in specimen the ifp attached to the anterior acl fiber via the lmuc and in specimen it attached directly to the central anterior tibia proximal ifp extension were identified alongside the patella in all specimen and visible on mri medially of specimen mean length mm laterally mean length mm mean ifp volume wa ml the lmuc attached near the base of the middle ifp lobe acting a a tether drawing it superiorly during knee extension the medial lobe consistently had a pedicle superomedially positioned between the patella and medial trochlea mri scan demonstrated how the space between the anterior tibia and patellar tendon the anterior interval narrowed during knee flexion displacing the ifp superiorly and posteriorly a it conformed to the trochlear and intercondylar notch surface conclusion proximal ifp extension are a novel description the ifp is a dynamic structure displacing significantly during knee motion which is therefore vulnerable to interference from trauma or repetitive overload given that this trauma is often surgical it may be appropriate that surgeon learn to minimize injury to the fat pad at surgery european society of sport traumatology knee surgery arthroscopy esska |
| Perioperative Pain Management | analgesia, analgesic, bupivacaine, mg, morphine, nerve block, consumption, block, postoperative pain, local | additional benefit of local infiltration of analgesia to femoral nerve block in total knee arthroplasty doubleblind randomized control study purpose multimodal analgesia ha become an important concept in current pain management following total knee arthroplasty tka however controversy remains over what is the most accepted combination in this study the additional benefit of local infiltration of analgesia to femoral nerve block were evaluated method forty patient were randomly allocated into a combined local infiltration of analgesia and femoral nerve block or femoral nerve block alone group in the former analgesic drug consisting of ropivacaine and dexamethasone were injected into the periarticular tissue while the same amount of saline wa injected into the femoral nerve block group the primary outcome measure wa the total amount of fentanyl consumption by the patientcontrolled analgesia pump during the h postoperative period result a combination of local infiltration of analgesia and femoral nerve block had le total fentanyl consumption and a larger knee rom at postoperative day than femoral nerve block alone p creactive protein level in the combined treatment group were significantly lower than the femoral nerve block group at postoperative day p there wa no difference between the two group postoperatively on the visual analogue scale for pain at rest or while walking quadriceps strength timed up and go test circumference of thigh knee society score and western ontario and mcmaster university osteoarthritis index conclusion the addition of local infiltration of analgesia to femoral nerve block promoted postoperative pain relief and the recovery of knee rom in the early postoperative period this combination is an effective method for postoperative pain management after tka level of evidence randomized controlled trial level i european society of sport traumatology knee surgery arthroscopy esska | a comparison of intraarticular morphine and bupivacaine for pain control and outpatient status after an arthroscopic knee surgery under a low dose of spinal anaesthesia effective pain control is important after an outpatient arthroscopic knee surgery to permit early discharge and improve outcome the aim of this study wa to compare intraarticular morphine and bupivacaine with placebo for postoperative pain control and outpatient status after a knee arthroscopic surgery under a low dose of spinal anaesthesia after obtaining the ethic committee approval and written informed consent from adult outpatient undergoing knee arthroscopy patient were enroled in this prospective randomized doubleblinded placebocontrolled clinical study all patient received spinal anaesthesia with ml of hyperbaric bupivacaine patient were randomly divided into three group a morphine group m n bupivacaine group b n and placebo group c n after the surgical procedure patient received one of the following solution intraarticularly in a doubleblinded randomized manner mg morphine in ml saline ml bupivacaine or ml saline postoperative pain wa assessed using a cm visual analogue scale va patient characteristic hemodynamic value sensory and motor block va value rescue analgesic discharge time and patient satisfaction were recorded there were no significant difference in patient characteristic surgery and tourniquet time hemodynamic value and sensory and motor block the va value at and min were similar among the three group the va value at rest and during move were higher in group c than in group m and b at min and h p there wa no difference in va value between the group m and b rescue analgesic used and discharge time were significantly different in the placebo group when compared to group m and b p side effect were similar among the group patient satisfaction score were high in the group m and b administration of mg morphine and ml of bupivacaine intraarticularly provides better pain relief and shorter discharge time without increasing the side effect than placebo for an outpatient arthroscopic knee surgery performed under a low dose of spinal anaesthesia springerverlag | pre and postoperative intraarticular analgesia for arthroscopic surgery of the knee and arthroscopyassisted anterior cruciate ligament reconstruction a doubleblind randomized prospective study we tested the effectiveness of different intraarticular analgesic and of preemptive intraarticular analgesia for arthroscopyassisted anterior cruciate ligament reconstruction aclr and for operative knee arthroscopy eightytwo patient underwent operative knee arthroscopy under selective subarachnoid anaesthesia group a and patient underwent arthroscopyassisted aclr under general anaesthesia group b patient were randomly assigned to intraarticular analgesic treatment a follows group a morphine mg preoperative morphine mg morphine mg preoperative morphine mg bupivacaine ml bupivacaine ml morphine mg saline solution ml group b morphine mg morphine mg preoperative morphine mg bupivacaine ml bupivacaine ml morphine mg saline solution ml all opioids were diluted in ml of saline solution after postoperative administration the tourniquet wa left in place for min after preoperative administration the intraarticular surgical procedure wa delayed for about min in the postoperative period we recorded total consumption of ketoprofen given iv on demand a rescue analgesic treatment pain score before surgery and at t rd th th and th h occurrence of local anaesthetic or opioid sideeffects group a operative knee arthroscopy all morphine group and the bupivacaine group did not require ketoprofen postoperatively p v both group and pain score did not differ significantly among group the percentage of patient reporting higher pain score than before surgery wa larger in control group and in bupivacaine group respectively and lower in morphine group respectively group b aclr total consumption of ketoprofen wa lowest in group and p v all other treatment and v control group the percentage of patient who did not require any rescue analgesic wa in group in group in group and in all other group noside effect occurred in any patient intraarticular analgesia is safe and effective for arthroscopic knee surgery morphine provides a better pain control both in operative knee arthroscopy patient and in aclr a mg dose is adequate for operative knee arthroscopy but not for aclr where higher dosage are required mg preemptive intraarticular morphine provides better analgesia than postoperative administration |
| PFA | resurfacing, patellar resurfacing, pfa, patellar, patellofemoral, patellofemoral arthroplasty, patella, inlay, isolated patellofemoral, patellar thickness | no difference between resurfaced and nonresurfaced patella with a modern prosthesis design a prospective randomized study of total knee arthroplasty purpose despite numerous wellconducted study and metaanalyses the management of the patella during total knee arthroplasty tka remains controversial the aim of our study wa to compare the clinical and radiological outcome between patient with and without patellar resurfacing and to determine the influence of resurfacing on patellar tracking with a patellafriendly prosthesis method a singlecentered prospective randomized controlled study wa performed between april and november two hundred and fortyfive consecutive patient knee scheduled for tka were randomized for patellar resurfacing or patella nonresurfacing all patient received the same total knee prosthesis and were evaluated clinically and radiologically including the international knee society score ks knee and function forgotten joint score fjs anterior knee pain akp pain when climbing stair patellar tilt and patellar translation result two hundred and twentynine knee were available for clinical evaluation and knee for radiographic analysis the revision rate for patellofemoral cause wa case with no difference between the group p there wa no difference in survival rate between patellar resurfacing and nonresurfacing after month p there were no difference in ks functional component p ks knee component p fjs p and akp p at a mean followup of month there wa twice a much stair pain for the nonresurfacing group versus p there wa patellar tilt in of resurfaced knee n versus in nonresurfaced knee n p however there wa more patellar translation in the nonresurfaced group versus p there were no specific complication attributed to the patellar resurfacing procedure there were four secondary patellar resurfacing procedure in the nonresurfaced group after a mean of month postoperatively conclusion there is no superiority of patellar resurfacing or nonresurfacing in term of clinical or radiological outcome at midterm secondary patellar resurfacing is rare there is not enough evidence to recommend systematic patellar resurfacing with a patellafriendly prosthesis level of evidence european society of sport traumatology knee surgery arthroscopy esska | a matchedpair comparison of inlay and onlay trochlear design for patellofemoral arthroplasty no difference in clinical outcome but le progression of osteoarthritis with inlay design purpose to compare clinical and radiographic result after isolated patellofemoral arthroplasty pfa using either a secondgeneration inlay or onlay trochlear design the hypothesis wa that an inlay design will produce better clinical result and le progression of tibiofemoral osteoarthritis oa compared to an onlay design method fifteen consecutive patient undergoing isolated pfa with an onlay design trochlear component journey pfj smith nephew were matched with patient after isolated pfa with an inlay design trochlear component hemicap wave arthrosurface matching criterion were age gender body mass index and followup period an independent observer evaluated patient prospectively whereas data were compared retrospectively clinical outcome wa assessed using womac lysholm score and pain va kellgrenlawrence grading wa used to ass progression of tibiofemoral oa result conversion to total knee arthroplasty wa necessary in one patient within each group leaving patient per group for final evaluation the mean followup wa month in the inlay group and month in the onlay group n both group displayed significant improvement of all clinical score p no significant difference were found between the two group with regard to the clinical outcome and reoperation rate no significant progression of tibiofemoral oa wa observed in the inlay group whereas of the onlay group showed progression of medial andor lateral tibiofemoral oa p conclusion isolated pfa using either a secondgeneration inlay or onlay trochlear component significantly improves functional outcome score and pain the theoretical advantage of an inlay design did not result in better clinical outcome score however progression of tibiofemoral oa wa significantly le common in patient with an inlay trochlear component this implant design may therefore improve longterm result and survival rate after isolated pfa level of evidence iii european society of sport traumatology knee surgery arthroscopy esska | patellofemoral arthroplasty with onlay prosthesis lead to higher rate of osteoarthritis progression than inlay design implant a systematic review purpose the aim of this study wa to report the clinical and functional outcome complication rate implant survivorship and the progression of tibiofemoral osteoarthritis oa after new inlay or onlay patellofemoral arthroplasty pfa for isolated patellofemoral oa comparison of different implant type and model where it wa possible also represented one of the objective method a systematic literature search following prisma guideline wa conducted on pubmed scopus embase and cochrane database to identify possible relevant study published from the inception of these database until randomized control trial rcts case series case control study and cohort study written in english or german and published in peerreviewed journal after were included not original study case report simulation study systematic review or study that included patient who underwent tka or unicompartmental arthroplasty uka of the medial or lateral compartment of the knee were excluded additionally only article that assessed functional andor clinical outcome patientreported outcome prom radiographic progression of oa complication rate implant survival rate pain a well a conversion to tka rate in patient treated with pfa using inlay or onlay trochlea design were included for quality assessment the methodological index for nonrandomized study minor for noncomparative and comparative clinical intervention study wa used result the literature search identified article of them met all the inclusion criterion following the selection process median minor for noncomparative study value wa range and for comparative study range in term of clinical and functional outcome no difference between onlay and inlay pfa ha been described both design yielded satisfactory result at short medium and longterm followup both design improved pain postoperatively and no difference between them in term of postoperative va ha been noted although the onlay group presented a higher preoperative va when comparing the inlay to onlay trochlea design the inlay group displayed a lower progression of oa rate conclusion there is no difference in functional or clinical outcome after pfa between the new inlay and the onlay design with both presenting an improvement in most of the score that were used a higher rate of oa progression wa observed in the onlay design group level of evidence iii the author |
| OLT | talus, osteochondral, osteochondral lesion, lesion talus, olt, aofas, microfracture, talar, ankle, lesion | satisfactory longterm clinical outcome after bone marrow stimulation of osteochondral lesion of the talus purpose the purpose of the present study wa to evaluate the clinical and radiological outcome of arthroscopic bone marrow stimulation bm for the treatment of osteochondral lesion of the talus olts at longterm followup method a literature search wa conducted from the earliest record until march to identify study published using the pubmed embase ovid and cochrane library database clinical study reporting on arthroscopic bm for olts at a minimum of year followup were included the review wa performed according to the prisma guideline two author independently conducted the article selection and conducted the quality assessment using the methodological index for nonrandomized study minor the primary outcome wa defined a clinical outcome consisting of pain score and patientreported outcome measure secondary outcome concerned the return to sport rate reoperation rate complication rate and the rate of progression of degenerative change within the tibiotalar joint a a measure of ankle osteoarthritis associated confidence interval ci were calculated based on the primary and secondary outcome measure result six study with a total of ankle patient were included at a mean pooled followup of year the mean minor score of the included study wa out of point range indicating a low to moderate quality the mean postoperative pooled american orthopaedic foot and ankle society aofas score wa ci ci participated in sport at any level at final followup return to preinjury level of sport wa not reported reoperations were performed in ci of ankle and complication related to the bm procedure were observed in ci of ankle progression of degenerative change wa observed in ci of ankle conclusion longterm clinical outcome following arthroscopic bm can be considered satisfactory even though one in three patient show progression of degenerative change from a radiological perspective these finding indicate that olts treated with bm may be at risk of progressing towards endstage ankle osteoarthritis over time in light of the incremental cartilage damage cascade the finding of this study can aid clinician and patient with the shared decisionmaking process when considering the longterm outcome of bm level of evidence level iv the author | clinical outcome after arthroscopic microfracture for osteochondral lesion of the talus are better in patient with decreased postoperative subchondral bone marrow edema purpose magnetic resonance imaging mri finding of subchondral bone marrow edema sbme in osteochondral lesion of the talus olt after arthroscopic microfracture are associated with poor clinical outcome however the relationship between sbme volume change and clinical outcome ha not been analyzed it wa hypothesized that clinical outcome correlated with sbme volume change and extent of cartilage regeneration in patient with olt method patient who underwent arthroscopic microfracture for olt were followed up for more than year sbme volume change wa measured by comparing preoperative and year followup mri clinical outcome were assessed using the visual analogue scale va and the american orthopedic foot and ankle society anklehindfoot scale aofas at the year and final followup to compare clinical outcome patient were categorized into two group decreased sbme dsbme group case without sbme on either mri or with a decreased sbme volume between the mri and increased sbme isbme group case with new sbme on postoperative mri or with an increased sbme volume between the mri additionally the effect of age sex body mass index symptom duration olt size olt location containmentuncontainment preoperative subchondral cyst pre and postoperative sbme volume and mri observation of cartilage repair tissue score on clinical outcome were analyzed result the dsbme group included patient whereas the isbme group included the mean age wa year and mean followup period wa month preoperative sbme volume wa significantly higher in the dsbme group while the isbme group had higher volume at the final followup in both group the va and aofas score significantly improved at the final followup p the va score were significantly lower in the dsbme group at the year and final followup p while the aofas score were significantly higher p other factor including cartilage regeneration did not affect clinical outcome conclusion sbme volume change correlated with clinical outcome after arthroscopic microfracture for olt clinical outcome were worse in patient with new postoperative sbme and increased postoperative sbme volume in patient with an unsatisfactory clinical course that show decreased sbme via postoperative mri an extended followup in a conservative manner could be considered level of evidence level iii european society of sport traumatology knee surgery arthroscopy esska | osteochondral transplantation of autologous graft for the treatment of osteochondral lesion of talus to year followup purpose bone marrow stimulation procedure microfracturesdrilling are considered the gold standard for the primary treatment of osteochondral talar lesion in the literature there is lack of evidence about the appropriate treatment in case of failure of these procedure a technique of osteochondral autologous transplantation of talar graft wa used it wa hypothesized that this is a successful method with good result and low complication rate additionally a technique of anterior ankle approach with temporary removal of a bone block from the distal tibia that give adequate access to posterior talar dome lesion is demonstrated method between and patient male female with olt for which arthroscopic treatment with curettage and drilling or microfracture had failed underwent osteochondral transplantation with an osteochondral graft harvested from the ipsilateral talar articular facet a medial malleolar osteotomy or a distal tibial wedge osteotomy wa used to access the talar dome defect result the median followup time wa year range m thirtyfour lesion were located in the central talar dome in the coronal plane while and lesion were located in the lateral and medial aspect of talar dome in saggital plane respectively the overall improvement between the preoperative and postoperative aofas and va fa score wa point p and point p respectively clinical result were considered a good in patient and fair in three patient all the transplanted graft were observed to incorporate fully into the recipient bed no complication occurred at the site of the malleolus osteotomy or tibial osteotomy and the donor site at the talus conclusion the midterm result suggest that the technique of osteochondral transplantation of autologous talar graft for osteochondral lesion of talus after failure of primary treatment with bone marrow stimulation can be safely and successfully used it demonstrates excellent postoperative score including improvement of pain and function this procedure is combined with removal of a tibial bone block and it subsequent replacement and doe not yield complication experienced with other procedure level of evidence retrospective case series level iv european society of sport traumatology knee surgery arthroscopy esska |
